# A Mycorrhizal Bacteria Strain Isolated From *Polyporus umbellatus* Exhibits Broad-Spectrum Antifungal Activity

**DOI:** 10.3389/fpls.2022.954160

**Published:** 2022-07-18

**Authors:** Pengjie Han, Tianrui Liu, Yuan Zheng, Ruiqi Song, Tiegui Nan, Xiaolong Yang, Luqi Huang, Yuan Yuan

**Affiliations:** ^1^School of Pharmaceutical Sciences, Peking University, Beijing, China; ^2^State Key Laboratory of Dao-di Herbs, National Resource Center for Chinese Materia Medica, China Academy of Chinese Medical Sciences, Beijing, China; ^3^School of Pharmaceutical Sciences, South-Central University for Nationalities, Wuhan, China

**Keywords:** PGPB, *Pseudomonas* spp., biocontrol agents, biofertilizer, *Fusarium* spp., DAPG

## Abstract

The microbes in the rhizosphere (or mycorrhizosphere) could promote plant growth, however, it is unclear whether mycorrhizosphere microbes could fight multiple fungal pathogens. In this study, twenty-one bacterial strains distributed in 6 genera, including 5 *Pseudomonas* strains, were isolated from mycorrhizal samples of *Polyporus umbellatus* that rely on other fungi during their life cycles. Further screening and pot experiments showed that the *Pseudomonas* strain ZL8 not only inhibited the growth of phytopathogenic fungi, but also promoted the growth of *Salvia miltiorrhiza* through inhibiting its wilting. In addition, strain ZL8 was found to have the ability to dissolve phosphate, produce IAA and siderophore. Nineteen compounds were identified from the fermentation broth of strain ZL8, of which 2,4-diacetylphloroglucinol (DAPG) had a significant inhibitory effect on phytopathogenic fungi with a minimum inhibitory concentration of 3.12–25 μg/mL. Molecular docking predicted that DAPG could bind to myosin I at two unique sites, which may be responsible to the inhibition of fungal growth. The evaluation results showed that strain ZL8 can be used to develop a dual-purpose biocontrol agents and biofertilizer. These results also provide new insights into the discovery and utilization of new resources for biocontrol agents and biolfertilizers.

## Introduction

In recent years, the yield and the quality of many crops and medicinal plants, as well as vegetables and fruits have decreased because of plant diseases caused by soil-borne pathogens (Daria et al., 2020), including fungi. Fungal pathogens cause a range of serious plant diseases, such as Fusarium wilt, and are responsible for the most of the diseases in agricultural ecosystems, including some of the most devastating plant disease epidemics in history (Lee et al., 2002). There are two main ways to deal with the effects of these diseases: One is to develop disease-resistant plants, and the other is to use chemical fungicide to control the spread of pathogens (Kamal and Brian, 2006). However, these two methods have the disadvantages of long cycle and drug resistance of pathogens. A practical and sustainable strategy for dealing with phytopathogenic fungal diseases is therefore the application of biological control agents (BCAs). A proximate, practicable and sustainable strategy against phytopathogens is to replace the chemical control with the biocontrol. Biocontrol is the application of microbial antagonists (such as biocontrol agents; BCAs) to suppress plant diseases (Kamal and Brian, 2006; Saira et al., 2019).

Biological control agents (BCAs) have gained increasing attention for the control of plant pathogens to achieve agricultural sustainability without requiring the overuse of hazardous fungicide. Indeed, several bacteria have been isolated from plants and fungi, most of which belong to the genera *Bacillus* and *Pseudomonas* and exhibit satisfactory biological activity and marked biocontrol potential (Axel et al., 2012; Meyer et al., 2016; Mohamad et al., 2018). *Pseudomonas* is a diverse genus known for its environmental ubiquity and ability to produce a wide range of bioactive metabolites that are suitable as biocontrol agents (Weller, 1988). Moreover *Pseudomonas* is considered one of the most characterized biocontrol plant growth promoting bacteria (Singh et al., 2016). The well-characterized antibiotics produced by *Pseudomonas* include phenazine derivatives, quinolones, pyoluteorin, pyrrolnitrin, 2,4-diacetylphloroglucinol (DAPG), and pederin (Gross and Loper, 2009).

*Pseudomonas* are aerobic, motile, rod-shaped Gamma proteobacteria, which predominantly inhabit soil and aquatic environments (Inmaculada et al., 2015). In their natural habitats, they form mutualistic and antagonistic relationships with other prokaryotes, plants, fungi, and perform many ecological functions generally beneficial to agriculture (Dieter and Geneviève, 2005).

Among them, *Pseudomonas* is involved in bioremediation, plant growth promotion, and biocontrol (D’aes et al., 2010; de Alencar et al., 2017; Mauchline and Malone, 2017). DAPG is a low molecular weight, nitrogen-free, and non-volatile phenolic polyketone derived from phloroglucinol (Shanahan et al., 1992; Weller, 2007; Troppens et al., 2013). DAPG-producing *Pseudomonas* spp. is predominantly found in the rhizosphere of major dicotyledonous and monocotyledonous crops, such as banana, cotton, cucumber, maize, pea, tobacco, tomato, and wheat (de Souza et al., 2003; Saravanan and Muthusamy, 2006; Sumant et al., 2015; Raksha et al., 2019), and has been used as biocontrol agents when applied to these crops. The biocontrol agent is for root diseases such as the take-all of wheat (Fenton et al., 1992), potato soft rot (Cronin et al., 1997), and tomato crown and root rot (Duffy and Défago, 1997).

Another important feature of the bacteria is the direct promotion of plant growth. Plant growth-promoting bacteria (PGPB) live near plants and play a key role in the transformation of inorganic compounds, making nitrogen, phosphorus and potassium available for plant growth (Olanrewaju et al., 2017). Furthermore, these PGPB have beneficial effects on plant growth and yield by producing plant growth regulators such as indole-3-acetic acid (IAA), and gibberellic acid (Ali et al., 2019).

Due to their environmental friendliness and inability to induce resistance in pathogenic microorganisms, there is growing interest in developing new biocontrol agents from medicinal resources. *Polyporus umbellatus*, belonging to Polyporaceae in the class of Basidiomycetes has been used as medicine fungus in China for more than 2500 years (Xing et al., 2015). It is well known that the growth of *P. umbellatus* sclerotia depends on a symbiotic relationship with the forest pathogenic fungus *Armillaria gallica* (Kikuchi and Yamaji, 2010; Xing et al., 2013; Liu et al., 2015). In the progress of symbiosis establishment, the rhizomorph of *A. gallica* adheres and invades sclerotia of *P. umbellatus* and the *P. umbellatus* sclerotia launch effective defense responses to fend off *A. gallica* invasion (Liu et al., 2015). *A. gallica* is well known as a contributor to carbon cycling via woody tissue breakdown (Alveshere et al., 2020) and is used in *P. umbellatus* sclerotia cultivation in China. One potential source for biological control agents is the sclerotium of *P. umbellatus*, which has antiviral and antibacterial activity in the treatment of animal and human diseases.

*Salvia miltiorrhiza* is widely used in China and other Asia countries to treat cardiovascular diseases. More than 200 individual compounds have been isolated and characterized from *S. miltiorrhiza*, exhibiting various pharmacological activities targeting different pathways for the treatment of cardiovascular diseases in various animal and cell models (Wang et al., 2017). But in the process of cultivation of *S. miltiorrhiza*, the wilt disease is very serious. Wilt disease of *S. miltiorrhiza* leads to a reduction in the biomass of plant shoots and roots and a decrease in active components (Yang et al., 2013).

Biofertilization and biocontrol are distinct characteristics of heterogeneous PGPB that can be used to develop formulations to control pathogens, increase yield and food production by reducing reliance on the chemical fertilizers and fungicide (Sessitsch et al., 2010; Bhattacharyya and Jha, 2012). Therefore, this paper isolates and identifies bacteria from *P. umbellatus* sclerotia by independent and dependent culture methods, and analyzes the effects of the isolated bacteria on the growth of plant pathogens, as well as on the growth and disease resistance of *S. miltiorrhiza*. The genomes of the isolated bacteria are sequenced and analyzed biosynthesis of secondary metabolites, and their secondary metabolites with antifungal activity were isolated and identified. A future objective is to develop a dual-use inoculum with biofertilizer and BCA potential for commercial use in sustainable agriculture.

## Materials and Methods

### Collection of Sample and Isolation of Microorganisms

Sclerotia samples of *P. umbellatus* were collected from Ningshan, Shannxi Province, China (33^°^309″N, 108^°^828″E). The surface of sclerotia sample was sterilized with 70% ethanol, 2% hypochlorite (w/v) solution, and sterile water, and dilutions were spread on Luria-Bertani (LB) and incubated at 30°C for 5 days. Surface sterilization was considered effective when no microbial growth was observed in the medium.

One gram of surface-sterilized sclerotia samples were homogenate with 9 mL of sterile water. One mL of the suspension obtained was used to prepare serial dilutions. The dilutions 10^–3^ to 10^–7^ were selected for bacterial isolation. An aliquot of 100 μL from each of the dilutions was spread, respectively, on yeast mannitol agar (YMA), LB agar, and nutrient agar (NA) plates. All plates were incubated aerobically at 30^°^C for 2–5 days. All morphologically distinct colonies were picked and purified on LB agar plates by repeated subculturing.

### Diversity of Bacterial Community

Total genomic DNA was extracted from the sclerotium samples using the CTAB method. The 16S rRNA genes were amplified using the specific primers 515F (5′-GTGCCAGCMGCCGCGGTAA-3′) and 806R (5′-GGACTACHVGGGTWTCTAAT-3′) (Caporaso et al., 2011). Polymerase chain reaction (PCR) reactions were carried out using Phusion^®^ High-Fidelity PCR Master Mix (New England Biolabs, Ipswich, MA, United States). PCR products were purified with the Qiagen Gel Extraction Kit (Qiagen, Hilden, Germany). Sequencing libraries were generated using the TruSeq^®^ DNA PCR-Free Sample Preparation Kit (Illumina, San Diego, CA, United States) following the manufacturer’s recommendations. The library quality was assessed on a Qubit^®^ 2.0 Fluorometer (Thermo Fisher Scientific, Waltham, MA, United States) and Agilent Bioanalyzer 2100 system (Agilent Technologies, Santa Clara, CA, United States). The library was sequenced on an Illumina HiSeq 2500 platform, and 250-bp paired-end reads were generated.

FASTQ files from each sample were processed using Cutadapt version V1.9.1 software^1^ (Langille et al., 2013) for preliminary quality control of raw data. The clean sequencing reads were clustered into operational taxonomic units (OTUs) with 97% identity using UPARSE version 7.0.1001 software^2^ (Haas et al., 2011). According to the algorithm principle, sequences with the highest frequencies were selected as the representative sequences of OTUs.

QIIME2 software (Hall and Beiko, 2018) and the SSU rRNA database (Wang et al., 2007) of SILVA 132^3^ (Edgar, 2013) were used to perform species annotation analysis of the OUT sequences (set threshold 0.8–1) to obtain taxonomic information and statistics of the community composition of each sample at each classification level (kingdom, phylum, class, order, family, genus, and species). MUSCLE version 3.8.31 software (Quast et al., 2013)^4^ was used to perform rapid multiple sequence alignment to obtain the phylogenetic relationships of all OTU sequences.

The 16S sequence datasets reported in this paper have been deposited in the Genome Sequence Archive in National Genomics Data Center, China National Center for Bioinformation/Beijing Institute of Genomics, Chinese Academy of Sciences (GSA: CRA004358) that are publicly accessible at https://ngdc.cncb.ac.cn/gsa/browse/CRA004358.

### Phylogenetic Analysis

Genomic DNA of the isolate was extracted according to the method of Welington et al. (Araujo et al., 2002). The 16S rRNA gene was amplified by PCR using a pair of universal primers, 27F (5′-CAGAGTTTGATCCTGGCT-3′) and 1492R (5′-AGGAGGTGATCCAGCCGCA-3′), as previous described (Baik et al., 2008; Liu et al., 2022). The DNA gyrase subunit B (*gyrB*) gene was amplified with primers up-1 (5′-GAAGT CATCATGACCGTTCTGCAYGCNGGNGGNAARTTYGA-3′) and up-2r (5′-AGCAGGATACGGATGTGCGAGCCRTCNACR TCNGCRTCNGTCAT-3′) (Yamamoto and Harayama, 1995; Duan et al., 2021). The PCR mixture (50 μL) contained 2 μL of DNA template, 25 μL of 2 × M5 HiPer Taq Mix (Mei5bio, Beijing, China), 20 μL of ddH_2_O, and 2 μL of each primer (10 μM). 16S rRNA and *gyrB* genes were amplified in a thermal cycler (Applied Biosystems, United States) according to the following protocol: initial denaturation at 94°C for 10 min, followed by 35 cycles of denaturation at 94°C for 30 s, annealing at 55°C for 30 s, 1 min extension at 72°C and 10 min extension at 72°C.

The PCR product was purified and sequencing was performed by the Sanger method (RuiBiotech Co., Ltd, Beijing, China). Then, the almost-complete 16S rRNA and *gyrB* gene sequences were compiled and calculate the genetic distance with program CLUSTAL X (Kim et al., 2014). The sequences obtained were identified using the EzBioCloud platform^5^ (Yoon et al., 2017). The phylogeny of 16S rRNA and *gyrB* sequences was reconstructed by the neighbor-joining (NJ) with MEGA 7.0 software package (Tamura et al., 2011).

### Draft Genome Sequencing, Assembly, and Annotation

*Pseudomonas agarici* strain ZL8 genome was sequenced using a Pacbio sequel II and DNBSEQ platform (BGI, Shenzhen, China). Four SMRT cells Zero-Mode Waveguide sequencing arrays were used by the PacBio platform to generate the subreads set. PacBio subreads (< 1 kb in length) were removed. The program Canu is used for self-calibration. Draft genomic units are uncontroversial sets of fragments assembled using Canu, a high-quality set of corrected circular consensus sub-reads. To improve the accuracy of genome sequences, single-base correction was performed using GATK.^6^ The antiSMASH 6.0 pipeline (Blin et al., 2021) with relaxed assay strictness identified regions for the biosynthesis of secondary metabolites from the *P. agarici* strain ZL8.

The whole genome sequence data reported in this paper have been deposited in the Genome Warehouse in National Genomics Data Center (NGDC) (Chen et al., 2021; CNCB-NGDC Members and Partners, 2022), Beijing Institute of Genomics, Chinese Academy of Sciences/China National Center for Bioinformation, under accession number GWHBJCV00000000 that is publicly accessible at https://ngdc.cncb.ac.cn/gwh.

### Analysis of Morphological, Physiological, and Biochemical Taxonomic

Morphological, physiological, and biochemical characterizations, such as growth in different bacteriological media, temperature, and the Gram responses were performed according to the method of Li et al. (2020). Biochemical features were performed using the MIDI (Sherlock) and GENIII MicroPlates (Biolog) systems. MIDI were used to check the availability of 28 fatty acids sources and GENIII MicroPlates (Biolog) were used to check the availability of 71 carbon sources as described in the manufacturer’s instructions (Duan et al., 2021).

The tricalcium phosphate solubilizing ability of strain ZL8 was assessed using Pikovskaya (PVK) medium (Li et al., 2018). Strains ZL8 was screened for the ability of nitrogen fixation in nitrogen-free medium (Ashby) (Sen and Sen, 1965; Li et al., 2018). Strain ZL8 was tested with Salkowski’s reagent, which is commonly used to detect indoles (Li et al., 2018). The siderophore produced by strain ZL8 was determined by a chromoazurine S (CAS) assay (Himpsl and Mobley, 2019).

### Evaluation of Plant Growth-Promoting Abilities

Pot experiments were used to evaluate the plant growth promoting ability of strain ZL8. *S. miltiorrhiza* seeds were surface sterilized in 75% ethanol for 1 min and then in 10% hydrogen peroxide for 10 min; then, the seeds were rinsed 10 times with sterile water and germinated in an autoclaved commercial vermiculite-soil mixture for 3 days. The seedlings with uniform growth were transferred to plastic pots (9 cm in diameter, and 10 cm in depth) containing an autoclaved commercial vermiculite-soil mix. *S. miltiorrhiza* seedlings were inoculated with 20 mL of prepared bacterial suspension (1 × 10^8^CFU⋅mL^–1^) culture as bacterial treatments or with the same volume of sterile water as control. The 30-day-old plants were harvested, and fresh weight, plant and root length of *S. miltiorrhiza* seedlings was measured.

### Evaluation of Antifungal Activity *in vitro*

The *in vitro* antagonistic activity of strain ZL8 against the 9 fungal phytopathogens were tested in a double culture assay (Figure 3) as described by Sakthivel and Gnanamanickam (Sakthivel et al., 1986). A fungal disc (6 mm in diameter or length) was placed in the center of a potato dextrose agar (PDA) plate. Bacterial isolates were streaked 2 cm from the agar plug. Plates inoculated with fungal discs and sterile water were used as controls. Plates were incubated in the dark at 25°C; cultures containing *A. gallica* rhizomorphs were grown for 15 d, while those containing pathogen mycelium were grown for 7 d. Using the equation, results were expressed as the percent inhibition of fungal growth in the presence and absence of strain ZL8 (Montealegre et al., 2003). All experiments were performed in triplicate.

**FIGURE 1:**
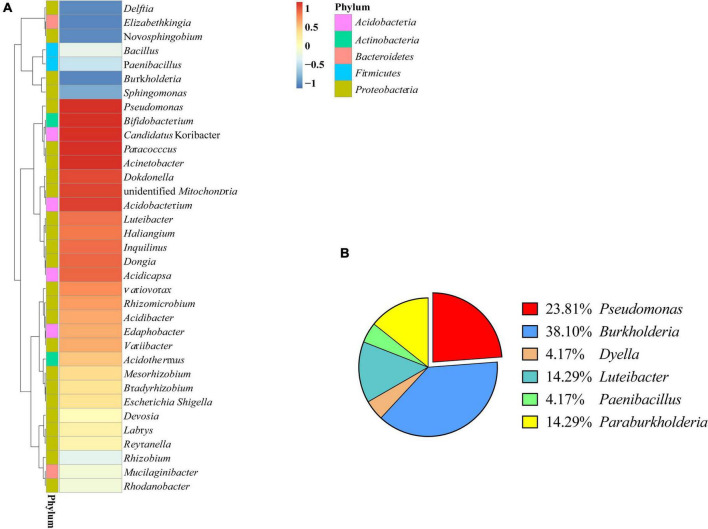
Bacterial diversity of *Polyporus umbellatus* sclerotia. **(A)**: Bacterial abundance in *P. umbellatus* sclerotia. **(B)**: Proportion of six genera bacteria isolated from *P. umbellatus* sclerotia.

**FIGURE 2:**
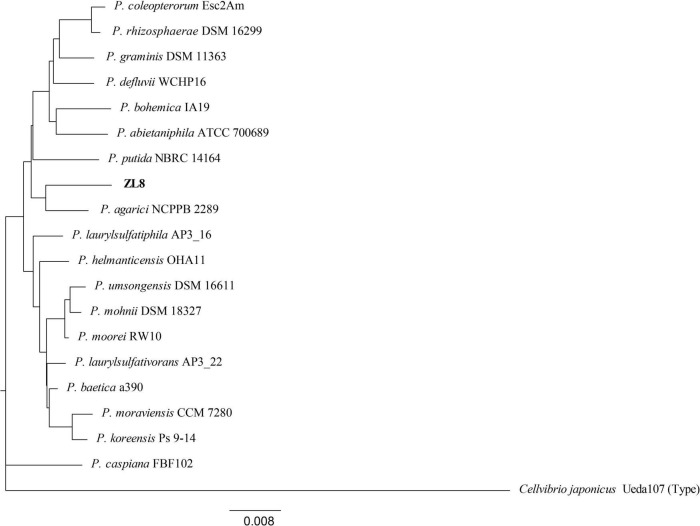
Phylogenetic tree of the strain ZL8 and its close relatives based on 16S rRNA gene sequencing. Dendrograms were generated by the neighbor-joining method. *Cellvibrio japonicus* Ueda 107T was used as outgroup. The bar indicates sequence divergence. Percentage bootstrap values of more than 50% (from 1000 replicates) are indicated at the nodes.

**FIGURE 3:**
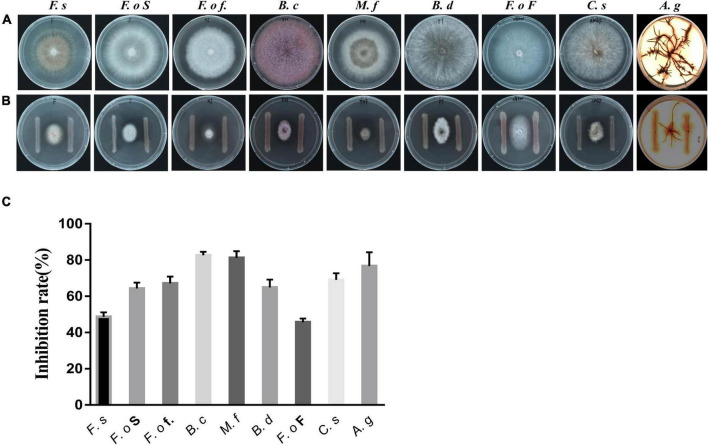
Effect of the isolated bacterial strain on the growth of fungi. **(A)**: Fungi treated with *Pseudomonas* strain ZL8; **(B)**: untreated fungi used as the control group; **(C)**: strain ZL8 inhibition rate for fungi. *F.s*: *Fusarium solani* (pathogen of *Salvia miltiorrhiza* root rot), *F.o* S: *F. oxysporum* (pathogen of *S. miltiorrhiza* wilt), *F.o* f.: *F. oxysporum* f. sp. Cubense (pathogen of panama disease), B.c: Botrytis cinerea (pathogen of tomato gray mold), M.f: Monilinia fructicola (pathogen of peach brown rot), B.d: *Botryosphaeria dothidea*, *F.o* F: *Fusarium oxysporum f. sp. niveum* (pathogen of Fusarium wilt), C.s: *Cochliobolus sativus* (pathogen of weat common rot), A.g: *Armillaria gallica.*


Inhibition(%)=[1-(fungalgrowth/controlgrowth)]×100

### Evaluation of Controlling the Wilt Disease of *Salvia miltiorrhiza* Seedlings

There were four control treatments in this experiment namely pathogenic fungi (*Fusarium oxysporum*, *F.o*), biocontrol (*F.o* + ZL8), control (ZL8) and sterile water (CK). The inoculation test was carried out when the *S. miltiorrhiza* seedlings reached the 2-leaf stage. The spore suspension of *F. oxysporum* (5 × 10^6^ CFU⋅mL^–1^, 20 mL) was added to the pot. After 7 days, the suspension of ZL8 strain (1 × 10^8^ CFU⋅mL^–1^, 20 mL) was added to the pot of biocontrol group, and then 20 mL of ZL8 strain suspension was added every 7 d for a total of 3 times. Meanwhile, the other groups added an equal volume of sterile water. Each group was repeated 15 times. 30 days after sowing, the plants were rated for root rot severity using a scale of 0 to 4: 0, healthy; 1, stem or root yellowing 1–10%; 2, stem or root yellowing 11–50%; 3, stems or roots yellowing 50–100%; 4, plant death (Leeman et al., 1996; Khabbaz and Abbasi, 2014).

At the time of rating, the total fresh mass was determined. All experiments were repeated at least twice. Biocontrol efficiency (B) were calculated using the following formula: *B*(%) = (*A*−*C*)/*A*×100. In the formula, A is the disease index of the seedlings group added only with strain ZL8, and C is disease index of the biocontrol agent group added with *F.o* and strain ZL8.

### Characterization of Antifungal Metabolites in Fermentation of the Strain ZL8

Strains stored at -80°C were streaked on LB agar plates and grown in an incubator at 28°C for 18 h. A single colony was picked and placed in five 500 mL Erlenmeyer flasks containing 100 mL of LB broth, incubated overnight at 28°C with shaking at 200 rpm to prepare the first broth. Then 500 mL of this first broth was transferred to a sterile 15 L fermenter containing 4.5 L LB broth at 30 °C, 1 vvm aeration for 12 h, shaking at 200–300 rpm. Then 3 L of second broth was transferred to a 50 L sterile fermenter containing 27 L of LB broth, and fermented for 72 h under the same culture conditions. A total of 150 L of bacterial fermentation broth was obtained.

The bacteria precipitate and the supernatant of the fermentation broth were separated by a continuous flow centrifuge, then the supernatant was extracted three times with ethyl acetate (EtOAc), and the organic solvent was removed under reduced pressure to obtain 21.0 g of a crude residue. The precipitate was sonicated three times with methanol (MeOH) and the organic solvent was evaporated to dryness under vacuum to give a crude residue 28.0 g. The combined crude extracts were subjected to silica gel (200–300 mesh) vacuum liquid chromatography using a gradient of petroleum ether/EtOAc to obtain five fractions (A–F). Fraction A was loaded in a silica gel (200–300 mesh) column and eluted with a gradient of petroleum ether/CH_2_Cl_2_ to yield eight fractions (A_1_–A_8_).

Fraction A1 (8.5 g) was purified after sequential chromatographies on silica gel (petroleum ether/CH_2_Cl_2_) to yield compounds **2** (19.8 mg) and **19** (12.0 mg). Fraction A_3_ (6.4 g) was further chromatographed in a Sephadex LH-20 column using acetone as mobile phase, followed by repeat cromatography on silica gel columns eluting with petroleum ether/CH_2_Cl_2_ to yield compounds **4** (8.0 mg), **5** (3.0 mg), **6** (4.0 mg), and **17** (6.0 mg). Fraction B was loaded onto a silica gel (200–300 mesh) column and eluted with a gradient of petroleum ether/EtOAc to yield five fractions (B_1_-B_5_). Compounds **7** (8.0 mg), **9** (4.2 mg), **10** (3.1 mg), **11** (3.8 mg), **12** (4.0 mg), and **13** (68.8 mg) from fraction B_1_ (8.6 g) after silica gel chromatography with petroleum ether/acetone as mobile phase and recrystallized. The constituents of fraction B_2_ (3.7 g) were separated after silica gel with petroleum ether/EtOAc as mobile phase and a subsequent washing in a Sephadex LH-20 column (CH_2_Cl_2_/MeOH, 1:1, v/v). It yielded compounds **8** (7.0 mg), **14** (4.6 mg), **15** (3.3 mg), and **18** (4.0 mg). Compounds **1** (4.1 mg), **2** (2.8 mg), and **16** (3.0 mg) were obtained from fraction B_3_ (2.8 g) in silica gel with petroleum ether/acetone as mobile phase, followed by a Sephadex LH-20 column (CH_2_Cl_2_/MeOH, 1:1), and semi-preparative HPLC with MeOH/H_2_O as mobile phase.

Compound **1**. White solid, 4.1 mg. ^1^H NMR (600 MHz, CD_3_OD): δ 5.79 (1H, s), 2.62 (6H, s). ^13^C NMR (150 MHz, CDCl_3_): δ 205.4, 172.8, 170.4, 105.1, 95.8, 33.2. HR-ESI-MS: m/z ([M + H]^+^) calcd for C_10_H_10_O_5_ 211.0606, found 211.0595.

Compound **2**. White solid, 2.8 mg. ^1^H NMR (600 MHz, CD_3_COCD_3_): δ 8.51 (3H, s), 5.96 (3H, s). ^13^C NMR (150 MHz, C_3_D_6_O): δ 159.3, 95.5. HR-ESI-MS: m/z ([M + H]^+^) calcd for C_6_H_6_O_3_ 127.0395, found 127.0378.

Compound **3**. Yellow powder, 5.0 mg. ^1^H NMR (600 MHz, CD_3_OD): δ 7.86 (2H, d, *J* = 8.7 Hz), 6.82 (2H, d, *J* = 8.7 Hz), 4.31 (2H, dd, *J* = 4.5, 9.8 Hz), 1.36 (3H, t, *J* = 7.1 Hz). ^13^C NMR (150MHz, CD_3_OD): δ 168.3, 163.4, 132.7 (X2), 122.3, 116.2 (X2), 61.7, 14.7. HR-ESI-MS: m/z ([M + H]^+^) calcd for C_9_H_10_O_3_ 167.0708, found 167.0713.

Compound **4**. White powder, 8.0 mg. ^1^H NMR (400 MHz, CD_3_OD): δ 6.97 (2H, d, *J* = 8.0 Hz), 6.68 (2H, d, *J* = 8.0 Hz), 3.64 (1H, t, *J* = 7.1 Hz), 2.66 (1H, t, *J* = 7.1 Hz). ^13^C NMR (100 MHz, CD_3_OD): δ 156.5, 130.9 (X2), 130.8 (X2), 116.1, 64.5, 39.2. HR-ESI-MS: m/z ([M + H]^+^) calcd for C_8_H_10_O_2_ 139.0759, found 139.9866.

Compound **5**. Colorless crystal, 3.0 mg. ^1^H NMR (600 MHz, CD_3_OD): δ 7.88 (2H, d, *J* = 8.6 Hz), 6.81 (2H, d, *J* = 8.6 Hz). ^13^C NMR (150 MHz, CD_3_OD): δ 163.3 (X2), 133.0 (X2), 116.0(X2).

Compound **6**. Colorless oil, 4.0 mg. ^1^H NMR (600 MHz, CD_3_OD): δ 6.61 (1H, d, *J* = 8.6 Hz), 6.59 (1H, d, *J* = 2.8 Hz), 6.54 (1H, dd, *J* = 8.6, 2.8 Hz), 3.67 (3H, s), 3.54 (2H, s). ^13^C NMR (150 MHz, CD_3_OD): δ 174.6, 151.0, 149.6, 123.2, 118.6, 116.6, 115.6, 52.4, 36.5.

Compound **7**. White powder, 8.0 mg. ^1^H NMR (600 MHz, CD_3_OD): δ 6.20 (1H, d, *J* = 2.5Hz), 6.15 (1H, d, *J* = 2.5 Hz), 3.89 (3H, s), 2.44 (3H, s). ^13^C NMR (150 MHz, CD_3_OD): δ 173.4, 166.2, 163.8, 144.5, 112.4, 105.7, 101.7, 52.1, 24.2. HR-ESI-MS: m/z ([M + H]^+^) calcd for C_9_H_10_O_4_ 183.0657, found 183.0648.

Compound **8**. Colorless crystal, 7.0 mg. ^1^H NMR (600 MHz, CD_3_OD): δ 7.08 (2H, d, *J* = 8.2 Hz), 6.74 (2H, m), 3.48 (2H, s); ^13^C NMR (150 MHz, CD_3_OD): δ 176.3, 157.3, 131.3 (X2), 126.7, 116.2 (X2), 41.0. HR-ESI-MS: m/z ([M - H]^+^) calcd for C_8_H_8_O_3_ 151.0395, found 151.0358.

Compound **9**. White powder, 4.2 mg. ^1^H NMR (600 MHz, CD_3_OD): δ 7.04 (2H, d, *J* = 8.5 Hz), 6.70 (2H, d, *J* = 8.5 Hz), 4.34 (1H, t, *J* = 9.0 Hz), 4.04 (1H, m), 3.55 (1H, m), 3.35 (1H, m), 3.06 (2H, m), 2.09 (1H, m), 1.80 (2H, m), 1.21 (1H, m). ^13^C NMR (150 MHz, CD_3_OD): δ 170.8, 166.9, 157.7, 132.1, 127.6, 116.2, 60.1, 57.9, 45.9, 37.7, 29.4, 22.7. HR-ESI-MS: m/z ([M + H]^+^) calcd for C_14_H_16_N_2_O_3_ 261.1239, found 261.1230.

Compound **10**. White acicular crystal, 3.1 mg. ^1^H NMR (600 MHz, C_5_D_5_N): δ 8.39 (2H, d, *J* = 7.9 Hz), 7.36 (2H, t, *J* = 16.6 Hz), 7.03 (2H, d, *J* = 8.3 Hz), 6.78 (2H, t, *J* = 15.0 Hz). ^13^C NMR (150 MHz, C_5_D_5_N): δ 171.4, 152.3, 134.0, 132.3, 116.8, 115.5, 111.8.

Compound **11**. Colorless acicular crystal, 2.8 mg. ^1^H NMR (600 MHz, CDCl_3_): δ 7.30 (5H, m), 3.66 (2H, s). ^13^C NMR (150 MHz, CDCl_3_): δ 178.2, 133.6, 129.6, 128.8, 127.5, 41.4. HR-ESI-MS: m/z ([M - H]^+^) calcd for C_8_H_8_O_2_ 135.0446, found 135.0412.

Compound **12**. Yellow solid, 4.0 mg. ^1^H NMR (600 MHz, CDCl_3_): δ 11.91 (1H, br s, OH), 7.43 (5H, m), 3.75 (2H, s). ^13^C NMR (150 MHz, CDCl_3_): δ 178.3, 133.1, 129.2, 128.5, 127.2, 40.9.

Compound **13**. Pale yellow crystal, 68.8 mg. ^1^H NMR (600 MHz, CD_3_OD): δ 7.81 (1H, dd, *J* = 8.1 Hz), 7.22 (1H, m), 6.72 (1H, dd, *J* = 8.1 Hz), 6.56 (1H, m). ^13^C NMR (150 MHz, CD_3_OD): δ 171.8, 152.9, 135.2, 132.8, 117.9, 116.8, 111.9. HR-ESI-MS: m/z ([M + H]^+^) calcd for C_7_H_7_NO_2_ 138.0555, found 138.0561.

Compound **14**. Pale yellow solid, 4.6 mg. ^1^H NMR (600 MHz, CD_3_OD) δ_*H*_: 7.52 (1H, d, *J* = 7.8 Hz), 7.31 (1H, d, *J* = 8.1 Hz), 7.07 (1H, m), 7.04 (1H, s), 7.00 (1H, m), 3.05 (2H, t, *J* = 8.8 Hz), 2.67 (2H, t, *J* = 8.8 Hz). ^13^C NMR (150 MHz, CD_3_OD) δ_*C*_: 177.7, 138.4, 128.7, 123.0, 122.5, 119.7, 119.4, 115.3, 112.4, 36.4, 22.1.

Compound **15**. White crystal, 3.3 mg. ^1^H NMR (600 MHz, CDCl_3_) δ_*H*_: 4.66 (2H, s), 4.31 (1H, s), 2.49 (6H, s), 2.38 (3H, s). ^13^C NMR (150 MHz, CDCl_3_) δ_*C*_: 149.5, 147.5, 147.4, 146.6, 60.8, 21.3, 21.2, 19.2. HR-ESI-MS: m/z ([M + H]^+^) calcd for C_8_H_12_N_2_O_2_ 153.1028, found 153.0526.

Compound **16**. White solid, 3.0 mg. ^1^H NMR (600 MHz, CD_3_OD): δ 7.95 (1H, s), 6.50 (1H, s), 4.41 (2H, s). ^13^C NMR (150 MHz, CD_3_OD): δ 177.0, 170.6, 147.5, 141.1, 110.9, 61.3.

Compound **17**. White solid, 6.0 mg. ^1^H NMR (600 MHz, D_2_O): δ 7.58 (1H, s), 6.98 (1H, d, *J* = 3.4 Hz), 6.53 (1H, m), 3.33 (3H, s). ^13^C NMR (150 MHz, D_2_O): δ 166.7, 149.0, 145.1, 115.0, 111.6, 48.8. HR-ESI-MS: m/z ([M + H]^+^) calcd for C_6_H_6_O_3_ 127.0395, found 127.9787.

Compound **18**. White solid, 4.0 mg. ^1^H NMR (600 MHz, CD_3_OD): δ 7.91 (1H, s), 6.46 (1H, s), 4.37 (2H, s). ^13^C NMR (100 MHz, CD_3_OD): δ 177.0, 170.4, 147.5, 141.0, 110.8, 61.2. HR-ESI-MS: m/z ([M–H]^+^) calcd for C_6_H_6_O_4_ 141.0188, found 141.0145.

Compound **19**. Pale yellow oil, 12.0 mg. ^1^H NMR (600 MHz, CDCl_3_): δ 2.42 (1H, dd, *J* = 16.0 Hz), 2.29 (1H, dd, *J* = 16.0 Hz), 1.32 (10H, m), 1.11 (2H, m), 0.88 (3H, t, *J* = 7.3 Hz), 0.74 (1H, m), -0.12 (1H, q, *J* = 5.0 Hz). ^13^C NMR (150 MHz, CDCl_3_): δ 180.3, 33.9, 32.1, 30.0, 29.4, 29.0, 22.8, 15.7, 14.3, 11.3, 11.0. HR-ESI-MS: m/z ([M–H]^+^) calcd for C_11_H_20_O_2_ 183.1385, found 183.1362.

### Antimicrobial Assays

The final fermentation broth, the EtOAc and MeOH extracts, and isolated metabolites were dissolved in DMSO to a concentration of 10 mg/mL. They were tested against the phytopathogenic fungi (Supplementary Table 3) by the filter paper method (Zhang et al., 2009). Ketoconazole and DMSO were used as positive and negative controls, respectively. Test fungi were activated in PDA medium. Next, a single pathogenic fungal colony was inoculated into a fresh PDA culture plate, and sterilized filter paper was placed on the plate. Finally, test sample was placed on the filter paper, then plates were incubated at 28°C for 2–3 d, and the size of the zone of inhibition was recorded.

The extracts and isolated compounds were also tested for resistance to the pathogens shown in Supplementary Table 4 by using the filter paper method. In this case, bacteria are activated in LB medium. Then, 200 μL of the bacterial broth was inoculated into warm (45 °C) LB agar medium on an inverted Petri dish. After cooling, the sterilized filter paper was placed on the culture plate. The test samples were then placed on the filter paper and the plate was incubated overnight at 37°C. Finally, the size of the inhibition zone was recorded. Ciprofloxacin and DMSO were used as positive and negative controls, respectively.

The minimum inhibitory concentration (MIC) of each compound against fungi was determined using a two-fold dilution method. The pathogenic fungi stored at 4°C were inoculated in PDA for activation. Next, each fungal plate was divided into equal-sized pieces using the block method and 2–3 pieces of fungus-containing medium were inoculated into 100 mL of potato dextrose broth. The cultures were incubated on a shaker at 28°C and 160 rpm for 2–3 d. After incubation, 1 mL of the fungal culture was diluted in 100 mL of potato dextrose broth, followed by adding 198 μL of diluted fungal suspension to the first row of the 96-well plate and 100 μL to the remaining rows. Next, 2 μL of the extract or solution of the isolated compound were added to the first row of wells and diluted 2-fold. The final concentrations of the samples were 100, 50, 25, 12.50, 6.25, 3.12, 1.56, and 0.78 μg/mL. The 96-well plate was incubated at 28°C for 48 h, 60 h, and 72 h, and the MIC values were recorded.

### Molecular Docking of TRI101 and 2, 4-Diacetylphloroglucinol

Molecular docking was performed using AutoDock Vina to predict binding modes of the compounds and obtain the binding affinity of DAPG for sites I and II of TRI101 (Zhou et al., 2020). The crystal structure of *F. graminearum* TRI101 (PDB ID: 6ui4) was downloaded from the RCSB Protein Data Bank (PDB), while the structure of DAPG was generated by Chem3D. TRI101 was prepared for docking with polar hydrogens added and Kollman charge was used, while the structure of DAPG was prepared by using Gasteiger charges. Both proteins and ligands are converted to pdbqt format and contain atomic coordinates, partial charges and solvation parameters. The energy range is set to 4, and the exhaustiveness is set to 8.

### Assessment of Antagonists for Their Commercial Value

The system established by Zheng et al. (2011) was used to evaluate the potential of the ZL8 as biocontrol and plant growth-promoting agents. Briefly, three categories were used using a point-based system: Category A provides scores for *in vitro* antagonistic activity against fungal pathogens based on the percent zone of inhibition (< 70% for 1 point, < 80% for 2 points and ≥ 80% for 3 points). The category B assigned 1 point for the presence of each of a biocontrol trait (antibiotics and siderophore), while the category C assigned 1 point for exhibiting plant growth promotion traits (phosphate solubilization, production of IAA, Nitrogen fixation). For the biocontrol strain, the points obtained are summarized and their commercial value was assessed (Ali et al., 2019).

## Results

### Bacterial Diversity Associated With *Polyporus umbellatus* Sclerotia

The bacteria associated with sclerotia of *P. umbellatus* were characterized by 16S rRNA gene sequencing. The 35 most abundant genera belonged to 5 bacterial phyla, of which were 3 Acidobacteria, 3 Actinobacteria, 2 Bacteroidetes, 5 Firmicutes, and the rest belonged to the Proteobacteria. A total of 13 genera had relative abundances greater than 1%, including *Bacillus*, *Pseudomonas*, *Burkholderia*, *Rhizobium*, and *Bradyrhizobium*. In *P. umbellatus* sclerotia, the dominant genera were *Acinetobacter* and *Pseudomonas*, with relative abundance of 4.32% and 2.13%, respectively (Figure 1A).

### Bacterial Strains Isolated From *Polyporus umbellatus* Sclerotia and Phylogenetic Analysis

The strains isolated from *P. umbellatus* sclerotium include six genera: *Burkholderia*, *Pseudomonas*, *Dyella*, *Luteibacter*, *Paebibacillus*, and *Paraburkholderia* (Figure 1B). Among them, *Pseudomonas* (23.81%) and *Burkholderia* (38.10%) were the bacteria with comparative advantage. Using BLAST, we identified five strains of *Pseudomonas*. To accurately determine the taxonomic status of strain ZL8, 16S rRNA and *gyrB* genes were sequenced. A similarity search was performed on 16S rRNA gene sequence of strain ZL8 in EZBioCloud, and it was found that strain ZL8 is likely belong to the genus *Pseudomonas*, and is the most closely related to *Pseudomonas agarici* (99.41%) and *Pseudomonas asplenii* (99.11%). A phylogenetic tree of strain ZL8 and its relatives based on 16S rRNA (Figure 2) and *gyrB* genes (Supplementary Figure 1) was constructed using MEGAX neighbor-joining method, which indicated that ZL8 isolate clustered with *Pseudomonas agarici*.

### Morphological and Biochemical Characteristics and Cellular Fatty Acids

Strain ZL8 is a rod-shaped Gram-negative bacterium. Its colonies are round and white with regular and translucent edges on TSA medium at 28°C (Supplementary Figures 5, 6). The characteristic C16:0 fatty acid of the genus *Pseudomonas* was present in this strain (Supplementary Table 1), similar to other *Pseudomonas* major fatty acid profiles (e.g., C10:0 3-OH, C12:0 2-OH, and C12:0 3-OH) (Lick et al., 2020). Biolog GENIII test provides phenotypic characteristics (Supplementary Table 2) also confirmed that strain ZL8 belongs to *Pseudomonas*.

### Antifungal Activity of *Pseudomonas* spp.

Five strains of *Pseudomonas* spp. were isolated and purified from the sclerotia of *P. umbellatus*. Antagonistic activity assays showed that *Pseudomonas* strain ZL8 inhibited the growth of *A. gallica* rhizomorphs and the mycelium of other pathogens (Figure 3). After strain ZL8 was co-cultured with *A. gallica*, the growth of the latter was significantly inhibited. Specifically, the rhizome length of *A. gallica* was 21.70% of the control without bacteria, thus showing a 78.30% reduction in length. The growth of other pathogens was also significantly inhibited, with the inhibition rate of all pathogens exceeded 44% (Figure 3 and Supplementary Table 5), indicating that strain ZL8 exhibited a significant inhibitory effect on plant pathogens.

The fungal growth inhibition observed in the co-culture experiments could be due to nutrient competition or the production of antifungal compounds (Thissera et al., 2020). To understand the mechanism of inhibition, strain ZL8 was grown under submerged fermentation and then the broth was filtered. Bacterial fermentation supernatants were collected on 1, 3, 4, and 6 d for co-cultivation of *A. gallica* rhizomorphs. After 4 d of fermentation, the bacterial broth had the most significant inhibitory effect on the growth of *A. gallica* rhizomorphs. *A. gallica* rhizomorph length in the treatment group was significantly reduced to 18.40% of that recorded in the unfermented control group. In addition, the number of rhizome branches and rhizome diameter of *A. gallica* in the control group were significantly larger than those in the treatment group (Supplementary Figure 3). These results are consistent with the co- cultures of bacteria and *A. gallica*, suggesting that this bacterium can produce metabolites that inhibit fungal growth.

### Effect of ZL8 Bacteria on the Growth of *Salvia miltiorrhiza*

**FIGURE 4:**
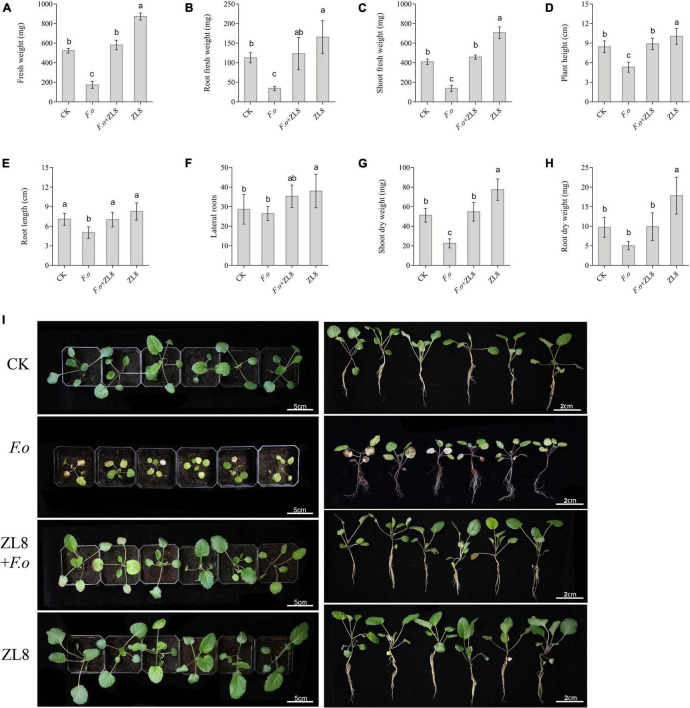
Effects of ZL8 inoculation on seedling growth of *Salvia miltiorrhiza*. **(A)**: Fresh weight, **(B)**: root fresh weight, **(C)**: shoot fresh weight, **(D)**: plant hight, **(E)**: root length, **(F)**: lateral root, **(G)**: shoot dry weight, **(H)**: root dry weight, and **(I)**: Phenotype of *S. miltiorrhiza* with different treatment groups. Seedlings were taken image and biomass were measured after 30 days inoculate to bacterials suspension and double-sterile distilled water, respectively. Values are means and bars indicate SDs (*n* = 6). Columns with different letters indicate significant difference at *P* < 0.05 (Duncan test).

The results of the antifungal experiment between strain ZL8 and phytopathogenic fungi showed that strain ZL8 had obvious inhibitory effect on the pathogenic fungi *in vitro*. The pot experiments were further carried out to evaluate the antifungal effect of the strain ZL8 on plants. The phenotype results of *S. miltiorrhiza* seedlings treated with different conditions showed that the disease symptoms in the *F.o* group were obvious (Figure 4I), and almost all plants had withered and fallen leaves, and the disease index was 58.33%. However, strain ZL8 and *F.o* group had only a few plant disease (Figure 4I), and the disease index was 20%, that is, the control efficiency of strain ZL8 against *S. miltiorrhiza* wilt was 65.71%.

The biomass of *S. miltiorrhiza* seedlings showed that the fresh weight, root fresh weight, aerial part fresh weight, plant height and dry weight of *S. miltiorrhiza* in the pathogen-treated group were lower than those in the control group (Figures 4A–H), and significantly decreased 66.83%, 69.45%, 66.11%, 37.04%, and 56.05%, respectively. The biomass of *S. miltiorrhiza* in the group treated with strain ZL8 and *F.o* increased significantly compared with the group treated with only *F.o*, and recovered to the same level as the control group. The results of biomass were consistent with the phenotypic results, indicating that the strain ZL8 had the effect of inhibiting the withering of *S. miltiorrhiza*.

Meanwhile, the phenotype and biomass of *S. miltiorrhiza* showed that only the ZL8-treated group grew faster and the biomass significantly increased compared with the control group (Figure 4). This result indicated that strain ZL8 also had the effect of promoting the growth of *S. miltiorrhiza*. Then, the appropriate inoculation amount of strain ZL8 was determined through experiments, and it was found that the ZL8 broth diluted 100 times was the best concentration to promote the growth of *S. miltiorrhiza* (Supplementary Figure 8).

In order to further study the reason why strain ZL8 promotes the growth of *S. miltiorrhiza*, experiments were carried out on the ability of strain ZL 8 to dissolve phosphorus, fix nitrogen, and produce siderophore and IAA. The results showed that strain ZL8 could produce about 36.5 μg/mL IAA from LB cultures. Strain ZL8 also produces siderophore, which is consistent with the gene cluster analysis of secondary metabolites in the genome. Strain ZL8 was unable to grow on the nitrogen-free Ashby medium, but strain ZL8 was able to dissolve phosphate (Supplementary Figure 4).

### Genes Associated With Plant-Interaction Life Style in *Pseudomonas agarici* Strain ZL8

Based on the genome sequence of *P. agarici* strain ZL8 (7.21 Mb), the biosynthetic gene clusters was further analyzed. The antiSMASH 6.0 pipeline with relaxed assay strictness identified 13 regions for the biosynthesis of secondary metabolites from the *P. agarici* strain ZL8 (Supplementary Figure 2). It contains gene clusters for DAPG biosynthesis with 100% similarity to the reference gene cluster from *Pseudomonas* genomospecies (Supplementary Figure 2). It also contains gene clusters for putisolvin (100% of genes showing similarity), arylpolyene (APE Vf, 45% similarity), L-2-amino-4-methoxy-trans-3-butenoic acid (40% similarity), pyoverdin (29% similarity), lankacidin C (13% similarity), malleobactin A-D (7% similarity).

### Separation and Identification of Compounds From ZL8 Fermentation Products

**FIGURE 5:**
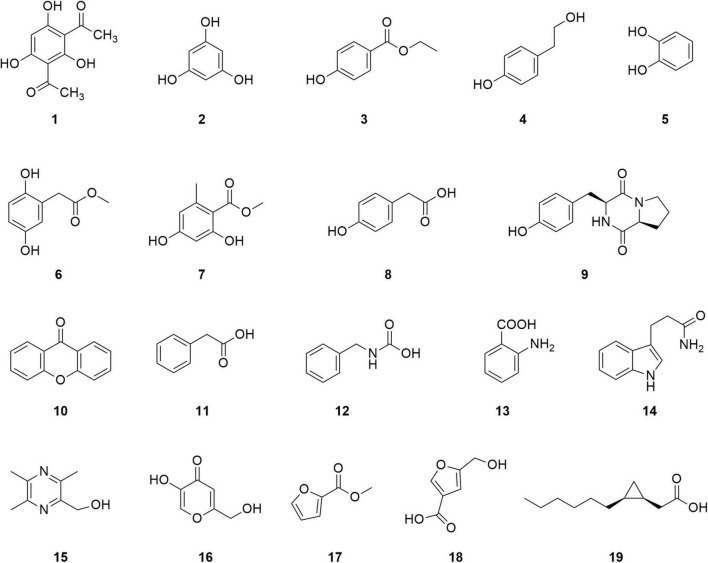
Structure of compounds **1–19**.

*P. agarici* strain ZL8 was cultured under submerged fermentation, and secondary metabolites were extracted and isolated from the fermentation broth. A total of 19 compounds were characterized (Figure 5), 14 of which had benzene ring structures; nine of these benzene ring-containing compounds were found to contain hydroxyl groups and, therefore, were considered to be phenolic.

The compounds are identified as follows: DAPG (**1**), a characteristic compound of a plant growth-promoting *Pseudomonas* with antifungal activity (Raksha et al., 2019); phloroglucinol (**2**), the precursor of DAPG; ethyl p-hydroxybenzoate (**3**); p-hydroxyphenethyl alcohol (**4**); catechol (**5**); 2, 5-dihydroxyphenylacetic acid methyl (**6**); methyl orsellinate (**7**); p-hydroxyphenylacetic acid (**8**); and **9** as cyclo (L-Pro-L-Tyr). All nine compounds were found to contain phenylhydroxyl structures. Four other compounds that also contained a benzene ring but no phenolic hydroxyl groups were identified as follows: compound xanthone (**10**), phenylacetic acid (**11**), N-benzyl carbamate (**12**), and anthranilic acid (**13**). Finally, five compounds without a benzene ring structure were identified: 2-hydroxymethyl-3,5,6-trimethylpyrazine (**15**), 5-hydroxy-2-hydroxymethyl-4H-pyran-4-one (**16**), methyl-2-furoate (**17**), 5-(hydroxymethyl) furan-3-carboxylic acid (**18**), and cis-2-(2-hexylcyclopropyl)-acetic acid (**19**). Among them, compounds **17** and **18** contain furan rings. The structural characterization of these products by MS and NMR analyses is provided in Supplementary Figures 9–60.

### Compound 1 Specifically Inhibits *Fusarium* spp.

Based on the filter paper method, all compounds were screened for their resistance to the test (pathogenic) fungi (*Verticillium dahliae* Kleb, *Alternaria mali*, *Rhizoctonia solani*, *Sclerotinia sclerotiorum*, *Gibberella saubinetii*, *Cordyceps militaris*, *Corynespora cassiicola*, *Fusarium. oxysporum* Dahl-1, *Fusarium* sp. Dahl-2, and *Fusarium. solani* Dahl-3) and bacteria (*Micrococcus lysodeikticus*, *Bacillus subtilis*, *Bacillus cereus*, *Staphylococcus aureus*, methicillin-resistant *Staphylococcus aureus*, *Salmonella paratyphi*, *Salmonella typhimurium*, *Pseudomonas aeruginosa*, and *Escherichia coli*). The EtOAc and water fractions of the compounds exhibited significant antifungal activity (Table 1); however, these fractions had no inhibitory effect against nine bacteria (Supplementary Table 7). These results suggest that the metabolites produced by *P. agarici* strain ZL8 have specific inhibitory effects on fungi.

**TABLE 1:** Antifungal activity of extracts from the fermentation broth of the bacteria isolated from *Polyporus umbellatus*.

Pathogenic fungi	Water fraction (cm)	Ethyl acetate fraction (cm)	Methanol fraction (cm)	Ketoconazole (cm)
A	1.50 ± 0.14	1.60 ± 0.29	0.00	1.8 ± 0.14
B	1.00 ± 0.14	1.10 ± 0.22	0.50 ± 0.08	2.0 ± 0.14
C	0.80 ± 0.22	0.50 ± 0.14	0.00	2.3 ± 0.22
D	1.80 ± 0.08	1.30 ± 0.22	0.30 ± 0.08	2.6 ± 0.29
E	1.80 ± 0.14	1.80 ± 0.22	0.20 ± 0.00	2.4 ± 0.22
F	0.50 ± 0.14	0.90 ± 0.29	0.50 ± 0.08	2.5 ± 0.29
G	1.80 ± 0.22	1.50 ± 0.14	0.80 ± 0.14	2.0 ± 0.14
H	1.00 ± 0.16	1.00 ± 0.22	1.20 ± 0.08	1.8 ± 0.16
I	1.00 ± 0.14	1.10 ± 0.22	0.00	1.5 ± 0.00
J	1.00 ± 0.08	0.80 ± 0.14	0.50 ± 0.08	1.20 ± 0.22

*The anti-pathogenic fungal activities were displayed by the inhibition zone. A: Verticillium dahliae Kleb; B: Alternaria mali; C: Rhizoctonia solani; D: Sclerotinia sclerotiorum; E: Gibberella saubinetii; F: Cordyceps militaris; G: Corynespora cassiicola; H: Fusarium oxysporum Dahl-1; I: Fusarium sp. Dahl-2; J: F. solani Dahl-3.*

Analysis of the antifungal activity of each isolated compound showed that compound **1** was inhibitory against a variety of phytopathogenic fungi (Table 2 and Supplementary Table 6), with MIC values between 3.12 and 12.5 μg/mL. Compound **1** exhibited the strongest antifungal effect against *Fusarium* (MIC 6.25–25 μg/mL). The MIC for *F. solani* Dahl-3, *F. oxysporum* Dahl-1, and *Fusarium* sp. Dahl-2 were 12.5%, 25%, and 25% of ketoconazole, respectively (Table 2). This indicated that *Fusarium* was 4–8 times more sensitive to compound **1** than the ketoconazole-positive control group. Compound **1** is expected to specifically inhibit the growth of *Fusarium* spp.

**TABLE 2:** Inhibitory effects of diacetylphloroglucinol (DAPG) against fungal pathogens.

Pathogenic fungi	DAPG	CK	Pathogenic fungi	DAPG	CK
*V. dahliae* Kleb	12.5	<0.78	*Fusarium* sp. Dahl-2	6.25	25
*A. mali*	>100	0.78	*F. solani* Dahl-3	12.5	>100
*R.solani*	6.25	3.12	*F. solani*	25	100
*S. sclerotiorum*	6.25	<0.78	*F. solani*	6.25	12.5
*G. saubinetii*	6.25	6.25	*F. proliferatum*	12.5	100
*C. militaris*	3.12	3.12	*F. oxysporum* Schlecht	12.5	50
*C. cassiicola*	>100	<0.78	*F. oxysporum* f. sp. vesinfectum (Atk) Snyder & Hansen.	>100	>100
			*F. oxysporum* Dahl-1	25	100

*CK: ketoconazole. Minimum inhibitory concentration (MIC) was 100 μg/mL; the fungus grew at a concentration of 100 μg/mL.*

To test this hypothesis, the inhibitory activity of compound **1** against five other *Fusarium* spp. was tested. Compound **1** exhibited satisfactory antifungal activity against four strains of *Fusarium* spp., with MIC value 4–8-fold lower than ketoconazole (Table 2), supporting the special inhibitory activity of DAPG against *Fusarium* spp.

### Assessment of ZL8 for Commercial Value as a Biofertilizer and/or a Biopesticide

*Pseudomonas agarici* strain ZL8 were rated for their antagonistic, biocontrol and plant growth-promoting potentials (Table 3). The data showed that the biocontrol strain ZL8 achieved 29 points out of a total of 32 points. The rating system is based on *in vitro* assessment of biocontrol capabilities and is directly related to the performance of strains in plant growth chamber studies. Therefore, strain ZL8 was rated as a strain with commercial potential based on Zhang’s rating system.

**TABLE 3:** Assessed potentials of ZL8 as biocontrol and PGPR agents.

Strains	Antagonism against 9 fungal pathogens^a^									Biocontrol potential^b^		PGP potential^c^			Assessed commercial value
	*F.o*	*F.m*	*R.s*	*C.g*	*C.f*	*A.n*	*A.f*	*A.a*	*A.g*	Antibiotics	Sid.	P	IAA	N	Total = 32
ZL8	2	3	3	3	3	3	3	3	2	1	1	1	1	0	29

*^a^Indicates sizes of inhibition zones of ZL8 antagonists toward 9 fungal pathogens 1 point represents 0–1 mm wide zone; 2 point represents 1–3 mm wide zone; 3 point represents >3 mm wide zone.*

*^b^Indicates activities of antifungal metabolites. 1 point represents antagonist showing antibiotics and siderophore production (sid.). 1 point represents that ZL8 possesses such trait while 0 point represents no ability.*

*^c^Indicates activities of plant growth-promoting potential including solubilizing phosphate (P), production of IAA, fixing nitrogen (N); P, Phosphate solubilization; IAA, Production of indole-3-acetic acid; N, Nitrogen fixation. 1 point represents ZL8 showing the said activity; 0 point represents ZL8 showing no these abilities.*

## Discussion

Many bacteria found around plant roots (rhizosphere and/or mycorrhizosphere) have the ability to promote plant growth and are therefore known as plant growth-promoting bacteria (PGPB) (Sessitsch et al., 2010; Liu et al., 2022). Few studies have reported the ability of PGPB to promote plant growth and its antagonistic potential against a variety of fungal pathogens. Therefore, this study aimed to explore the potential of antifungal agents to control a broad host-range fungal pathogens while exerting stimulatory effects on plant growth.

Biocontrol agents with broad-spectrum antimicrobial activity are more promising under field conditions than biocontrol agents with antagonistic activity against only one or two pathogens (Zhang et al., 2017; Saira et al., 2019). Biocontrol agents were found from the sclerotia of the fungus *Polyporus umbellatus*, which are partially or completely dependent on mycorrhizal fungi for their life cycle (Liu et al., 2015; Lu et al., 2019) and are presumed to have a library of antifungal agents. In this study, 21 bacteria in 6 genera were isolated, and a bacterium of the genus *Pseudomonas* exhibits strong antagonistic activity against phytopathogenic fungi.

*Pseudomonas* is an important non-pathogenic biocontrol agent that has been extensively studied and found to elicit antagonistic activity against several pathogens, including *F. oxysporum* f. sp. *Cucumerinum* (Chin-A-Woeng et al., 1998; Mcspadden et al., 2000), *Colletotrichum lagenarium*, *Phytophthora capsici*, *Pythium aphanidermatum*, *Pythium ultimum*, *Sclerotinia sclerotiorum*, *F. oxysporum* f. sp. *cucumerinum*, *Corticium sasakii*, *Rhizoctonia solani* (Liu et al., 2007), and *Gaeumannomyces graminis* var. *tritici* (Chin-A-Woeng et al., 1998). In this study, *P. agarici* strain ZL8 has strong inhibitory activity against the growth of 9 plant pathogens, and the inhibition rate of *in vitro* co-culture screening tests reached 45.69–82.62%. Strain ZL8 also has a control effect on *S. miltiorrhiza* wilt, and the control efficiency of pot experiments is 65.71%.

*Pseudomonas agarici* strain ZL8 has broad-spectrum antifungal activity and potential as a biocontrol bacteria. The strain ZL8 was identified as *Pseudomonas agarici* with a genome of 7.21M. Among the 13 regions identified by secondary metabolite biosynthesis, DAPG and putisolvin biosynthesis with 100% similarity to the reference gene cluster from other *Pseudomonas* genome (Supplementary Figure 2). Putisolvin, including putisolvin I and putisolvin II, have the ability to reduce surface tension, inhibit biofilm formation and break down biofilms of *Pseudomonas* species including *P. aeruginosa* (Irene et al., 2004; Dubern et al., 2008). These two gene clusters for DAPG and arylpolyene biosynthesis (45% similarity, Supplementary Figure 2) have been identified as contributing to the biocontrol activity of *Pseudomonas bijieensis* (Dutta et al., 2020).

Phloroglucinols is a phenolic compounds produced by *Pseudomonas* spp., plants, and algae (de Souza et al., 2003). More than 60 phloroglucinol derivatives have been described and reported with antiviral, antibacterial, antifungal, antihelminthic, phytotoxic, antitumor, and plant growth regulating activities (de Souza et al., 2003). DAPG, a derivative of phloroglucinol, has antifungal activity and can inhibit soil-borne diseases (Meyer et al., 2016). DAPG-producing *Pseudomonas* generally exhibited higher plant protective activity than biocontrol *Pseudomonas* that did not produce DAPG (Rezzonico et al., 2007). In this study, we found that strain ZL8 contained a DAPG biosynthesis gene cluster, and isolated DAPG from the fermentation broth of strain ZL8, and we also found that DAPG has broad-spectrum antifungal activity and was the most potent inhibitory compound against *Fusarium* spp. 4–8-fold higher than the positive control group.

To further analyze the mechanism by which DAPG inhibits the growth of *Fusarium*, molecular docking was performed using DAPG with sites I and II of protein crystal structures of *Fusarium* TRI101 (PDB ID: 6ui4) (Supplementary Figure 7B) and TRI101 is related to the growth and pathogenicity of *Fusarium* (Zhou et al., 2020). The lowest binding energies obtained for sites I and II were 5.9 and 5.8 kcal/mol, respectively, indicating that DAPG has a higher binding affinity for site I than for site II. DAPG is mainly surrounded by hydrophobic bonds formed by internal pocket residues, including Ile67, Tyr68, Ser77, Pro80, Phe81, Gly136, Glu139, Ala140, Arg143, Ser287, Ile290, and Ala291. DAPG formed hydrogen bonds with polar residues in the cluster inside the site I pocket, such as Phe8, Arg82, and Tyr87, with bond distances of 2.7 Å, 2.5 Å, and 2.4 Å, respectively. In addition, molecular docking studies showed structural evidence of the drug similarity of DAPG and its binding mode to site I.

*Fusarium* belong to the familiar group of filamentous fungi in agriculture and forests. This group includes many species that cause devastating diseases in major crops, such as wheat, maize, rice, and barley, causing severe yield losses and mycotoxin contamination in infected grains (Zhou et al., 2020). Myosins consists of a superfamily of ATP-driven molecular motors involved in a variety of cellular processes, including muscle contraction, vesicular trafficking, cytokinesis, organelle movement, and sensory transduction. Myosins are grouped into 35 classes based on sequence homology, of which myosin II is the conventional myosin responsible for muscle contraction (Ross et al., 2008; Hartman and Spudich, 2012). Furthermore, only a single class I myosin exists in all *Fusarium* species (Zhou et al., 2020). In this study, DAPG has a unique inhibitory effect on *Fusarium*, and molecular docking results show that DAPG binds to two class I myosin sites, providing a basis for further research on the antifungal mechanism of DAPG.

Pyoverdine is a complex mixed-type siderophore containing hydroxamate and catecholate groups (Schalk et al., 2011; Bouizgarne, 2013) synthesized by strains of *P. putida*, *P. syringae*, and *P. aeruginosa* (Bouizgarne, 2013). The strain ZL8 genome contains the for Pyoverdine biosynthetic gene cluster with a similarity of 29% (Supplementary Figure 2) compare with *Pseudomonas protegens* (Stintzi et al., 1999). In the chrome azurol S (CAS) assay, strain ZL8 was found to have the ability to produce siderophore. In addition, strain ZL8 has the ability to produce IAA and dissolve phosphate. In general, plant growth-promoting bacteria have the ability to produce siderophores, as well as the ability to produce growth IAA, phosphate solubilization and nitrogen fixation (Karimi et al., 2012; Ali et al., 2019). This indicates that strain ZL8 has the potential to become a plant growth-promoting bacteria.

Previously reported commercial value assessment strategy (Zheng et al., 2011) have been used to evaluate some fungi (Zheng et al., 2011) and bacteria (Karimi et al., 2012; Ali et al., 2019) as biocontrol agents and biofertilizers. The biocontrol agent potential and biofertilizer ability of strain ZL8 were evaluated using this system and scored 29 points (out of 32 points), indicating that strain ZL8 has commercial value as a biocontrol agent and biofertilizer.

## Conclusion

In the current study, various fungi-associated (mycorrhizal-like) bacteria, including *Pseudomonas agaricus* ZL8, were isolated from the sclerotia of *P. umbellatus*. Strain ZL8 has a significant inhibitory effect on phytopathogenic fungi, and has an inhibitory effect on *S. miltiorrhiza* wilt indicating that strain ZL8 can be used as a biological control of phytopathogenic fungi. At the same time, it was also found that strain ZL8 has the effect of promoting plant growth, significantly increasing the biomass and growth rate of plants, and its mechanism of promoting plant growth is to solubilize phosphate, produce siderophore and IAA. Furthermore, the antifungal mechanism of strain ZL8 is the production of DAPG, which has broad-spectrum resistance to plant pathogens. This study provides support for the development of *P. agaricus* ZL8 for biocontrol agents and bio fertilizers, and also provides new insights into the discovery and utilization of new resources for biocontrol agents and biolfertilizers.

## Data Availability Statement

The whole genome sequence data in this study can be found in the National Genomics Data 595 Center (NGDC) with accession number: GWHBJCV00000000 (https://ngdc.cncb.ac.cn/gwh/Assembly/25481/show). The 16S sequence datasets for this study can be found in the NGDC with GSA number: CRA004358 (https://ngdc.cncb.ac.cn/gsa/browse/CRA004358).

## Author Contributions

YY and LH designed the experiments. PH, XY, YZ, and RS performed the experiments. PH and TL analyzed the data and wrote the original draft manuscript. TN collected the phytopathogenic fungi strains. YY revised the manuscript. All authors have read and agreed to the published version of the manuscript.

## Conflict of Interest

The authors declare that the research was conducted in the absence of any commercial or financial relationships that could be construed as a potential conflict of interest.

## Publisher’s Note

All claims expressed in this article are solely those of the authors and do not necessarily represent those of their affiliated organizations, or those of the publisher, the editors and the reviewers. Any product that may be evaluated in this article, or claim that may be made by its manufacturer, is not guaranteed or endorsed by the publisher.

## Footnotes

1
http://cutadapt.readthedocs.io/en/stable/

2
http://www.drive5.com/uparse/

3
http://www.arb-silva.de/

4
http://www.drive5.com/muscle/

5
https://www.ezbiocloud.net/

6
https://www.broadinstitute.org/gatk/
