# Supplementary material for: A Mycorrhizal Bacteria Strain Isolated From Polyporus umbellatus Exhibits Broad-Spectrum Antifungal Activity
Source: Front Plant Sci. 2022 Jul 18;13:954160. doi: 10.3389/fpls.2022.954160 (PMC9340266; doi:10.3389/fpls.2022.954160)
Supplement: Supplementary file 1 [file Data_Sheet_1.PDF]

Supplementary Material

1 Supplementary Figures and Tables

1.1 Supplementary Tables

Supplementary Table 1. Cellular fatty composition of *Pseudomonas* ZL8.

Fatty acids	Percent (%)	Comment1	Comment2
$C_{10:0}$ 3OH	11.78		
$C_{12:0}$	4.74		
$C_{12:0}$ 2OH	16.35		
$C_{12:0}$ 3OH	11.42		
$C_{14:0}$	0.38		
$C_{16:0}$	9.58		
$C_{18:0}$	0.48		
Sum In Feature 7	0.39		
Summed Feature 3	10.15	$C_{16:1}$ ω7c/$C_{16:1}$ ω6c	$C_{16:1}$ ω6c/$C_{16:1}$ ω7c
Summed Feature 8	4.17	$C_{18:1}$ ω7c	$C_{18:1}$ ω6c

The strain were cultured on tryptone soy agar (TSA) and incubated at 28 ˚C for 48 h. Summed feature represents a set of more than one CFA that could not be resolved: summed feature 3 contained $C_{16:1}$ ω7c/16:1 ω6c and/or $C_{16:1}$ ω6c/$C_{16:1}$ ω7c and summed feature 8 contained $C_{18:1}$ ω7c and/or $C_{18:1}$ ω6c.

Supplementary Table 2. The result of Biolog GENIII test for phenotypic characteristics.

BIOLOG	result	BIOLOG	result	BIOLOG	result	BIOLOG	result
**Negative Control**	-	α-D-Glucose	+	Gelatin	-	p-Hydroxy-Phenylacetic Acid	-
Dextrin	-	D-Mannose	-	Glycyl-L-Proline	-	Methyl Pyruvate	-
D-Maltose	-	D-Fructose	-	L-Alanine	-	D-Lactic Acid Methyl Ester	-
D-Trehalose	-	D-Galactose	+	L-Arginine	-	L-Lactic Acid	+
D-Cellobiose	-	3-Methyl Glucose	-	L-Aspartic Acid	W	Citric Acid	+
Gentiobiose	-	D-Fucose	-	L-Glutamic Acid	W	α-Keto-Glutaric Acid	-
Sucrose	-	L-Fucose	-	L-Histidine	+	D-Malic Acid	-
D-Turanose	-	L-Rhamnose	-	L-Pyroglutamic Acid	-	L-Malic Acid	+
Stachyose	-	Inosine	-	L-Serine	-	Bromo-Succinic Acid	-
**Positive Control**	+	1% Sodium Lactate	+	Lincomycin	+	Nalidixic Acid	-
pH 6	+	Fusidic Acid	+	Guanidine HCl	+	Lithium Chloride	-
pH 5	W	D-Serine	-	Niaproof 4	+	Potassium Tellurite	+
D-Raffinose	-	D-Sorbitol	-	Pectin	-	Tween 40	-

α-D-Lactose	-	D-Mannitol	-	D-Galacturonic Acid	-	γ-Amino-Butryric Acid	W
D-Melibiose	-	D-Arabitol	-	L-Galactonic Acid Lactone	-	α-Hydroxy-Butyric Acid	-
β-Methyl-DGlucoside	-	myo-Inositol	-	D-Gluconic Acid	+	β-Hydroxy-D,L-butyric Acid	-
D-Salicin	-	Glycerol	-	D-Glucuronic Acid	-	α-Keto-Butyric Acid	-
N-Acetyl-DGlucosamine	-	D-Glucose-6-PO4	-	Glucuronamide	W	Acetoacetic Acid	-
N-Acetyl-β-DMannosamine	-	D-Fructose-6-PO4	W	Mucic Acid	+	Propionic Acid	-
N-Acetyl-DGalactosamine	-	D-Aspartic Acid	-	Quinic Acid	+	Acetic Acid	+
N-Acetyl Neuraminic Acid	-	D-Serine	-	D-Saccharic Acid	+	Formic Acid	-
1% NaCl	+	Troleandomycin	+	Vancomycin	+	Aztreonam	-
4% NaCl	-	Rifamycin SV	+	Tetrazolium Violet	+	Sodium Butyrate	-
8% NaCl	-	Minocycline	-	Tetrazolium Blue	+	Sodium Bromate	-
pH (optimum)	9	Temperature (optimum)	28 ℃				

+, posive; -, negtive; w: weak.

Supplementary Table 3. Information of fungal pathogens studied.

Strain	Scientific name	Host and disease	disease
A	*Verticillium dahliae* Kleb	Potato	Verticillium wilt of potato
B	*Alternaria mali*	Cabbage	Black leaf spot disease
C	*Rhizoctonia solani*	Rice	Rice sheath blight
D	*Sclerotinia sclerotiorum*	Oil seed rape	Sclerotinia of oil seed rape (Sclerotinia sclerotiorum)
E	*Gibberella saubinetii*	Wheat	Wheat Scab
F	*Cordyceps militaris*	Insect larvae	Cordyceps militaris
G	*Corynespora cassiicola*	Cucumber	Brown spot
H	*Fusarium oxysporum* Dahl-1	*Salvia miltiorrhiza* Bunge	
I	*Fusarium* sp. Dahl-2	*Salvia miltiorrhiza* Bunge	Pathogenic fungi of Salvia miltiorrhiza Bunge
J	*Fusarium solani* Dahl-3	*Salvia miltiorrhiza* Bunge	
K	*Fusarium solani*	*Dendrobium moniliforme* (L.) Sw.	
L	*Fusarium tricinctum*	*Verbena officinalis* L.	

M	*Fusarium proliferatum*	*Campsis grandiflora* (Thunb.) Schum.	
N	*Fusarium oxysporum* Schlecht	Potato	Potato Fusarium wilt
O	*Fusarium oxysporum* f. sp. vesinfectum (Atk) Snyder & Hansen	Cotton	Cotton wilt
	Botrytis cinerea	Tomato	Tomato gray mold
	Monilinia fructicola	Peach	Peach brown rot
	Botryosphaeria dothidea		Leaf spot
	Cochliobolus sativus	Weat	Weat Common Rot

Supplementary Table 4. Information of the pathogenic bacteria studied.

Strain	scientific name
1	*Micrococcus lysodeikticus*
2	*Bacillus subtilis*
3	*Bacillus cereus*
4	*Staphyloccocus aureus*
5	*Methicillin-resistant Staphylococcus aureus*
6	*Salmonella paratyphi*
7	*Salmonella typhimurium*
8	*Pseudomonas aeruginosa*
9	*Escherichia coli*

Supplementary Table 5. Rate inhibition of fungi growth by ZL8 and the fermentation broth.

Strain	Inhibition rate			average
Armillaria gallica	78.39%	71.60%	74.57%	74.85%
Fusarium solani	47.52%	45.77%	51.90%	48.40%
Fusarium oxysporum	64.11%	61.24%	69.86%	65.07%
Fusarium oxysporum f. sp.	72.25%	62.50%	68.50%	67.75%
Botrytis cinerea	83.51%	82.24%	85.41%	83.72%
Monilinia fructicola	84.56%	85.29%	76.47%	82.11%
Botryosphaeria dothidea	67.31%	64.10%	58.97%	63.46%
Fusarium oxysporum f. sp.	48.38%	44.64%	43.14%	45.39%
Cochliobolus sativus	63.89%	73.61%	70.83%	69.44%
A. gallica (Fermentation broth of 4 d)	95.31%	85.26%	93.28%	91.28%

Supplementary Table 6. Inhibitory effects of compound 1-19 against fungal pathogens.

	A	B	C	D	E	F	G	H	I	J	K	L	M	N	O
**1**	12.5	>100	6.25	6.25	6.25	3.12	>100	25	6.25	12.5	25	6.25	12.5	12.5	>100
**2**	>100	100	>100	>100	>100	>100	>100	>100	>100	>100					
**3**	>100	>100	>100	>100	>100	>100	>100	>100	>100	>100					
**4**	>100	>100	>100	>100	>100	>100	>100	>100	>100	>100					
**5**	>100	>100	>100	>100	>100	>100	>100	>100	>100	>100					
**6**	>100	>100	>100	>100	>100	>100	>100	>100	>100	>100					
**7**	100	>100	100	100	50	100	>100	>100	>100	>100					
**8**	>100	>100	>100	>100	100	100	>100	>100	100	>100					
**9**	>100	>100	>100	>100	>100	>100	>100	>100	>100	>100					
**10**	>100	>100	>100	>100	>100	>100	>100	>100	>100	>100					
**11**	>100	>100	>100	>100	>100	>100	>100	>100	>100	>100					
**12**	>100	>100	>100	>100	>100	>100	>100	>100	>100	>100					
**13**	>100	>100	>100	>100	>100	50	>100	100	>100	>100					
**14**	>100	>100	100	>100	>100	25	>100	50	100	>100	>100	100	100	>100	>100
**15**	>100	>100	>100	>100	>100	>100	>100	>100	>100	>100					
**16**	>100	>100	>100	>100	>100	>100	>100	>100	>100	>100					
**17**	>100	>100	>100	>100	>100	>100	>100	>100	>100	>100					
**18**	>100	>100	>100	>100	100	>100	>100	>100	>100	>100					
**19**	50	>100	100	50	25	50	>100	100	100	>100					
**ct**	<0.78	0.78	3.12	<0.78	6.25	3.12	<0.78	100	25	>100	100	12.5	100	50	>100

Minimum inhibitory concentration (MIC) > 100 μg/mL; the fungus continued to grow at a concentration of 100 μg/mL. ct: ketoconazole; A: *Verticillium dahliae* Kleb; B: *Alternaria mali*; C: *Rhizoctonia solani*; D: *Sclerotinia sclerotiorum*; E: *Gibberella saubinetii*, F: *Cordyceps militaris*, G: *Corynespora cassiicola*, H: *Fusarium oxysporum* Dahl-1, I: *Fusarium* sp. Dahl-2, J: *Fusarium solani* Dahl-3. K: *F. solani*, L: *F. tricinctum*, M: *F. proliferatum*, N: *F. oxysporum* Schlecht, O: *F. oxysporum* f. sp. vesinfectum (Atk) Snyder & Hansen

Supplementary Table 7. Antibacterial activity of extracts from the fermentation broth of bacteria isolated from *Polyporus umbellatus*.

Pathogenic bacteria	Water fraction (cm）	Ethyl acetate fraction (cm）	Methanol fraction (cm）	Ciprofloxacin (cm)
1	0	0	0	1.6 ±0.08
2	0	0	0	1.6 ±0.12
3	0	0	0	1.6 ±0.09
4	0	0	0	1.5 ±0.05
5	0	0	0	1.5 ±0.09
6	0	0	0	1.6 ±0.17
7	0	0	0	1.5 ±0.09
8	0	0	0	1.5 ±0.05
9	0	0	0	1.5 ±0.05

Pathogenic bacteria (1-9): *Micrococcus lysodeikticus*, *Bacillus subtilis*, *Bacillus cereus*, *Staphylococcus aureus*, methicillin-resistant *Staphylococcus aureus*, *Salmonella paratyphi*, *Salmonella typhimurium*, *Pseudomonas aeruginosa*, *Escherichia coli*

1.2 Supplementary Figures

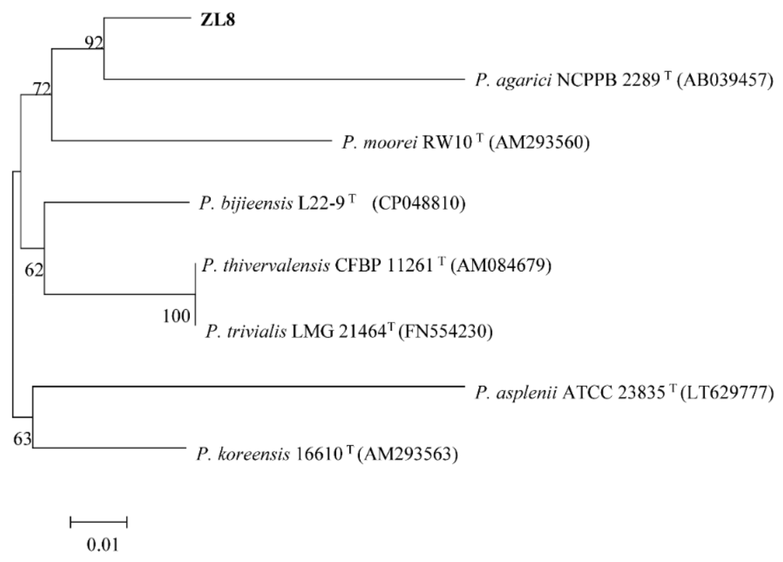

Supplementary Figure 1. Phylogenetic tree of ZL8 and close relatives based on concatenated sequences of the *gyrB* genes. Dendrograms were generated by the neighbour-joining method. The bar indicates sequence divergence. Percentage bootstrap values of more than 50% (from 1000 replicates) are indicated at the nodes.

Select genomic region:

Overview | 1.1 | 1.2 | 1.3 | 1.4 | 1.5 | 1.6 | 1.7 | 1.8 | 1.9 | 1.10 | 1.11 | 1.12 | 1.13

Identified secondary metabolite regions using strictness 'relaxed'

Chromosome1 (Bacteria)

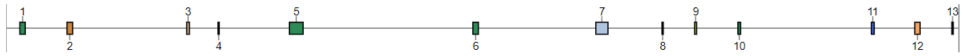

Region	Type	From	To	Most similar known cluster		Similarity
Region 1.1	NRPS-like	106,928	149,269	L-2-amino-4-methoxy-trans-3-butenoic acid	NRP	40%
Region 1.2	arylpolyene	473,349	516,953	APE Vf	Other	45%
Region 1.3	hserlactone	1,404,109	1,424,654			
Region 1.4	RiPP-like	1,647,210	1,656,047			
Region 1.5	NRPS	2,201,209	2,308,471	pyoverdin	NRP	29%
Region 1.6	NRPS	3,627,588	3,670,782	malleobactin A / malleobactin B / malleobactin C / malleobactin D	NRP:NRP siderophore	7%
Region 1.7	siderophore , NRPS	4,583,087	4,677,366	putisolvin	NRP	100%
Region 1.8	RiPP-like	5,097,484	5,107,133			
Region 1.9	NAGGN	5,351,250	5,365,931			
Region 1.10	CDPS	5,687,682	5,708,431			
Region 1.11	redox-cofactor	6,724,199	6,746,364	lankacidin C	NRP + Polyketide	13%
Region 1.12	T3PKS	7,062,105	7,103,130	2,4-diacetylphloroglucinol	Polyketide	100%
Region 1.13	RiPP-like	7,349,956	7,360,762			

Supplementary Figure 2. Compact view of gene clusters for biosynthesis of secondary metabolites. Compact view of gene clusters for biosynthesis of secondary metabolites identified by antiSMASH version 6.01 (https://antismash.secondarymetabolites.org/) using relaxed detection strictness from whole genome sequences of *Pseudomonas agarici* strains ZL8.

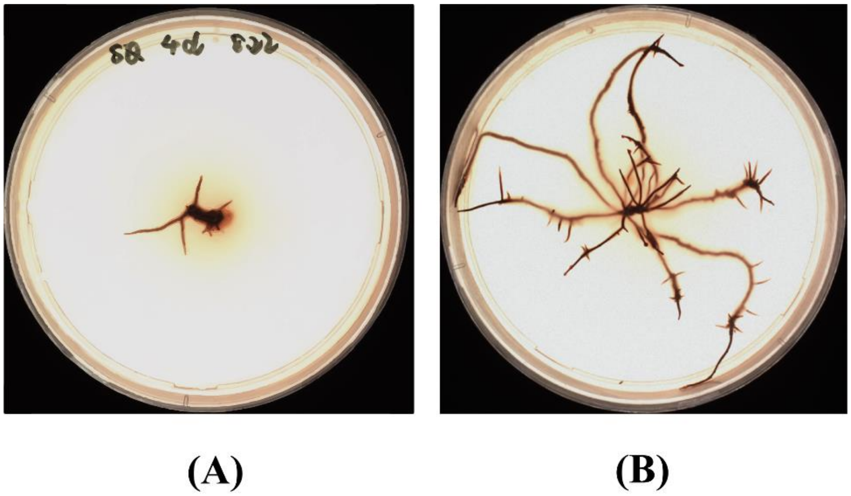

Supplementary Figure 3. The effect of ZL8 fermentation broths on the growth of *Armillaria gallica*. (A): *A. gallica* treated with 4 d-fermentation broths for 15 d; **(B)**: untreated *A. gallica* as control group.

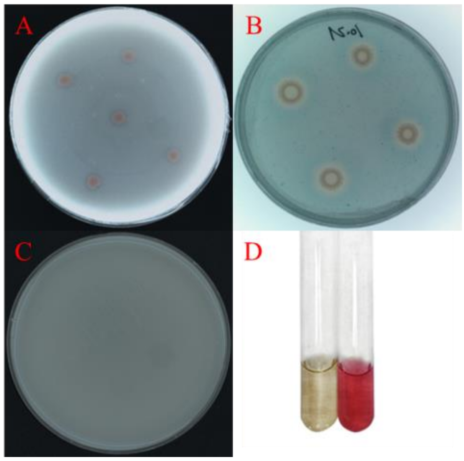

Supplementary Figure 4. The ability of strain ZL 8 to solubilize phosphate, fix nitrogen, and produce siderophore and IAA. A: solubilize phosphate by ZL8; B: ZL8 produce siderophore. C: Strain ZL8 did not grow on the nitrogen-free Ashby medium. D: strain ZL8 could produce IAA about 36.5 μg/mL IAA (y=0.0065x+0.0959, R^2=0.9995)

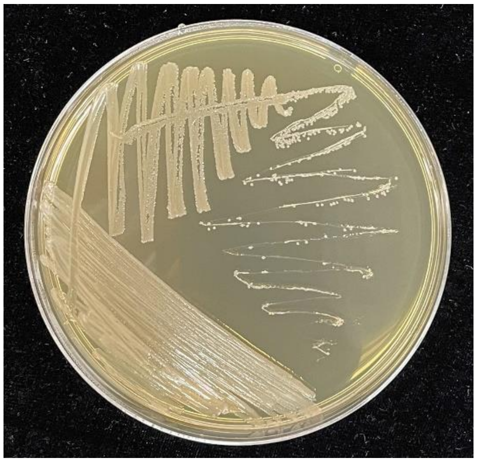

Supplementary Figure 5. Colony morphology of *Pseudomonas* ZL8.

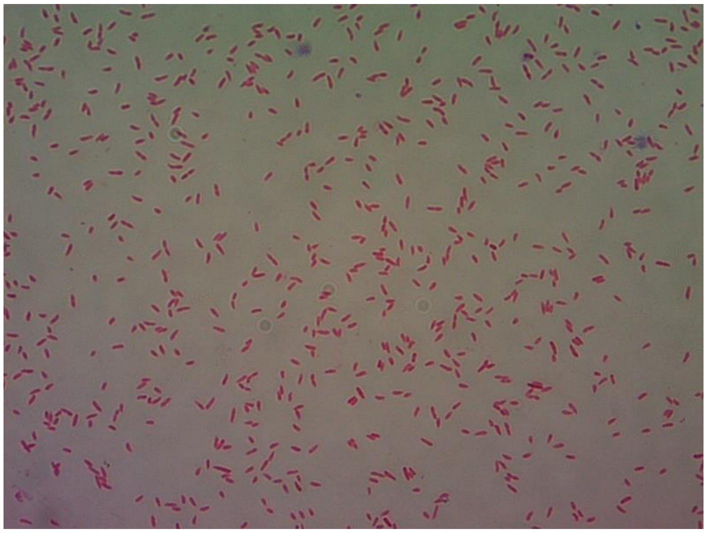

Supplementary Figure 6. Gram stain showing Gram negative.

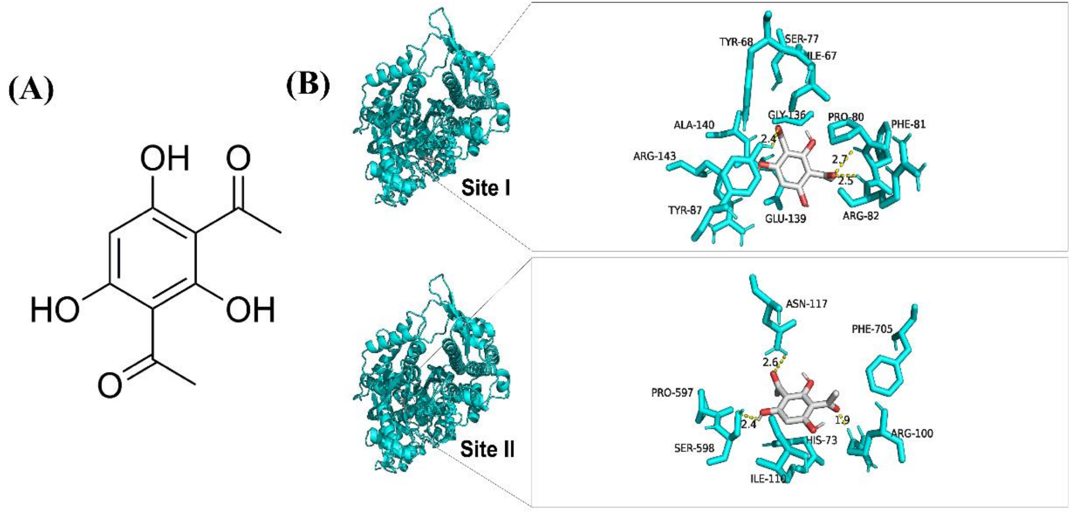

Supplementary Figure 7. Structure, antifungal activity, and protein-binding site prediction of compound 1. (A): Structure of compound **1**(DAPG). **(B):** Molecular docking of DAPG to sites I and II of TRI101-binding regions. Residues involved in H-bonds (dotted line); hydrophobic interactions are shown as sticks.

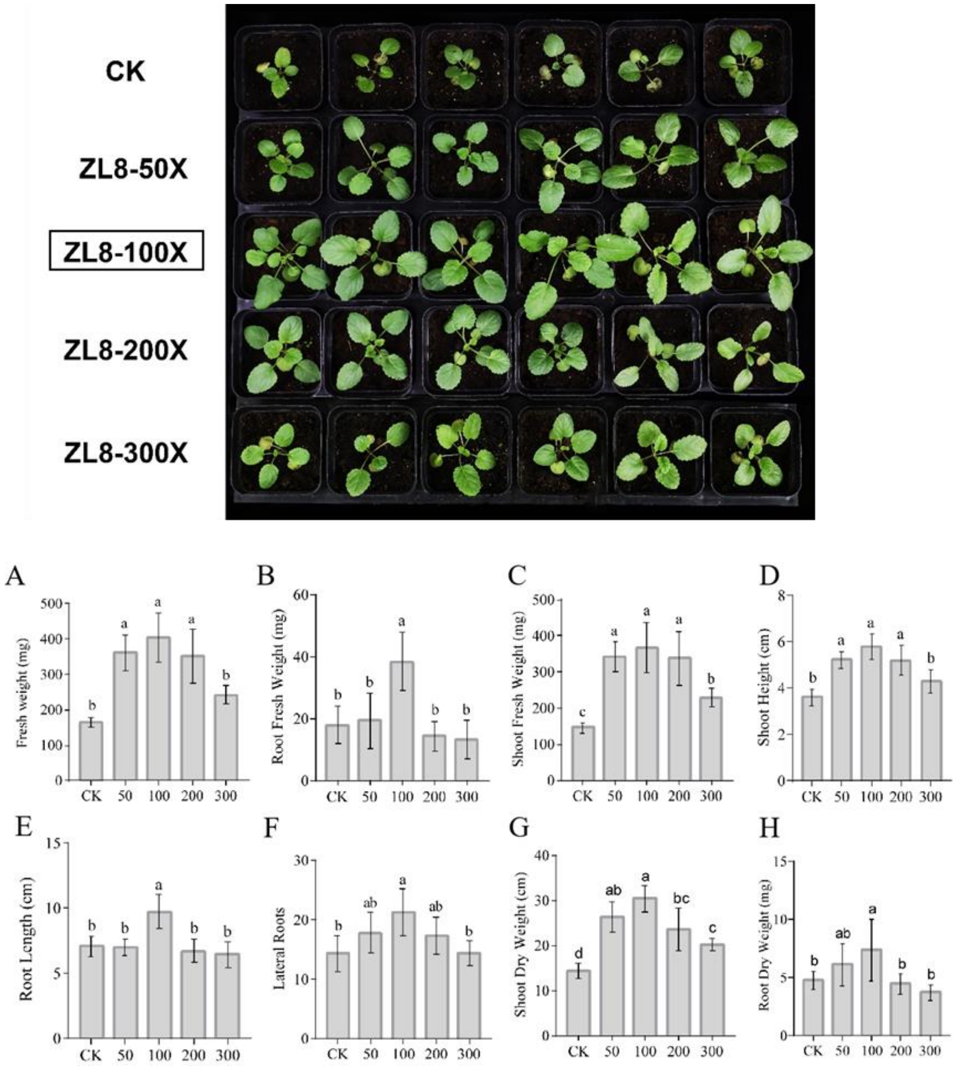

Supplementary Figure 8. ***Salvia miltiorrhiza* with different contribution of ZL8 inoculation groups.** A: fresh weight, B: root fresh weight, C: shoot fresh weight, D: shoot hight, E: root length, F: latcral root, G: shoot dry weight, and H: root dry weight. Seedlings were taken image and biomass were measured after 30 days inoculate to bacterials suspension and double-sterile distilled water, respectively. Values are means and bars indicate SDs (n = 6). Columns with different letters indicate significant difference at P <0.05 (Duncan test).

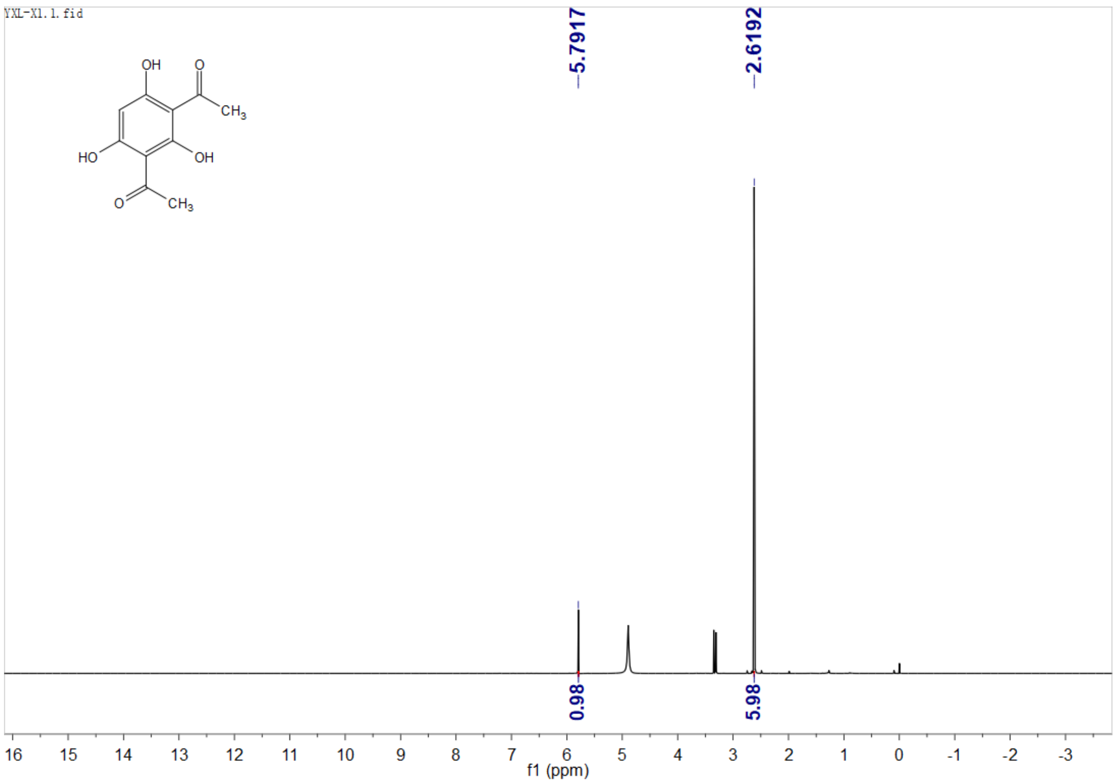

Supplementary Figure 9. ^{1}H NMR data for compound **1** (600 MHz, CD_3OD)

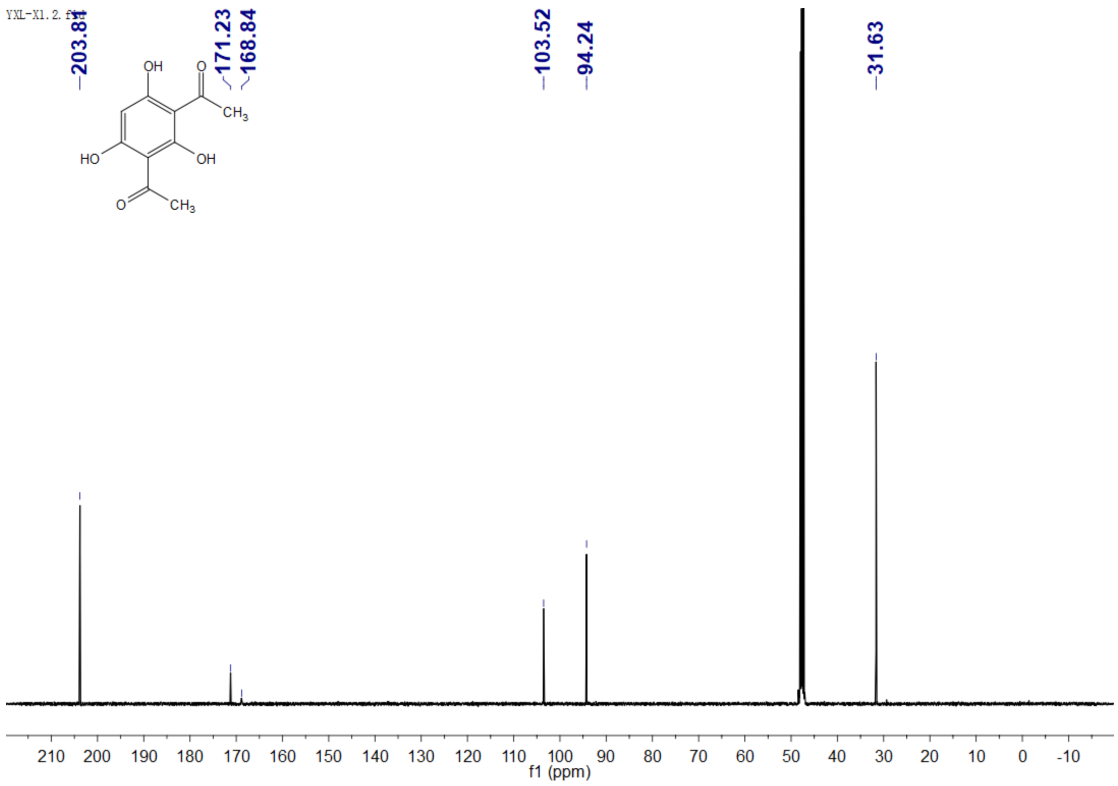

Supplementary Figure 10. ^{13}C NMR data for compound **1** (150 MHz, $CDCl_3$)

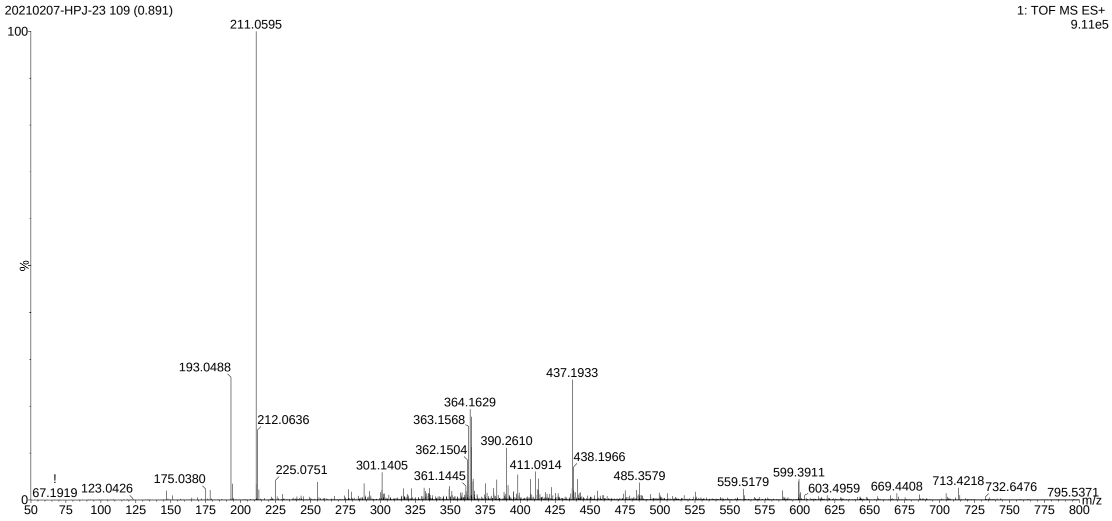

Supplementary Figure 11. HRMS data for compound **1**

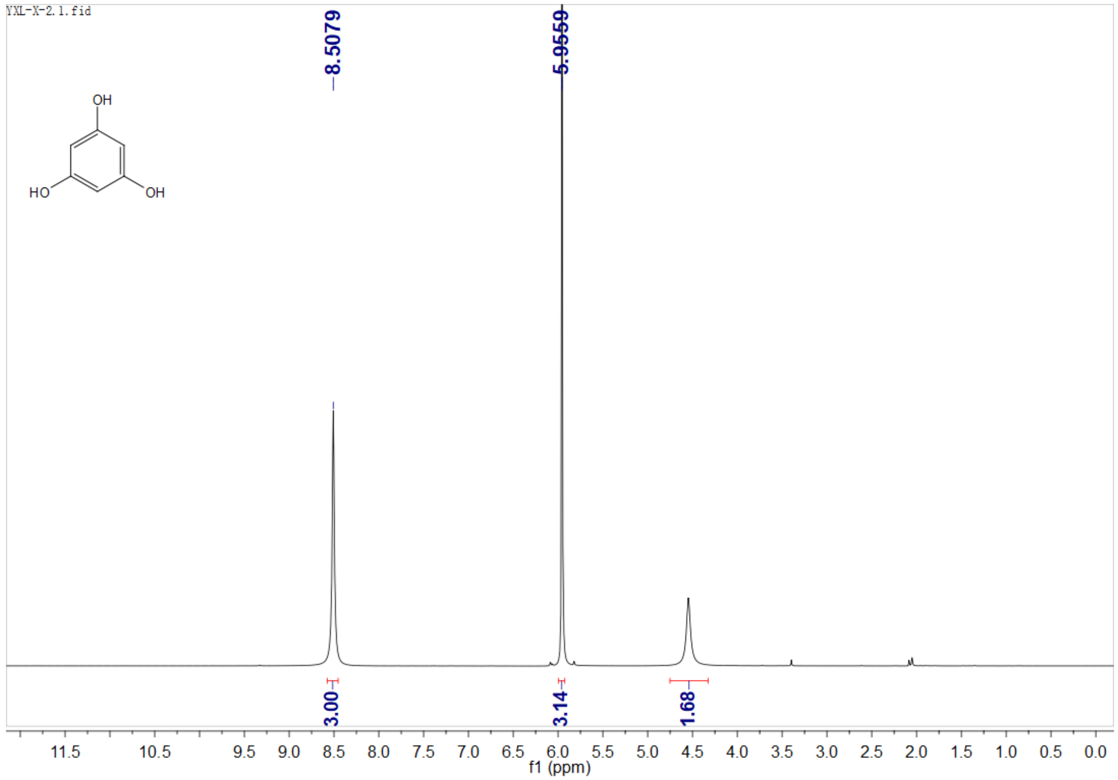

Supplementary Figure 12. ^{1}H NMR data for compound **2** (600 MHz, CD_3COCD_3)

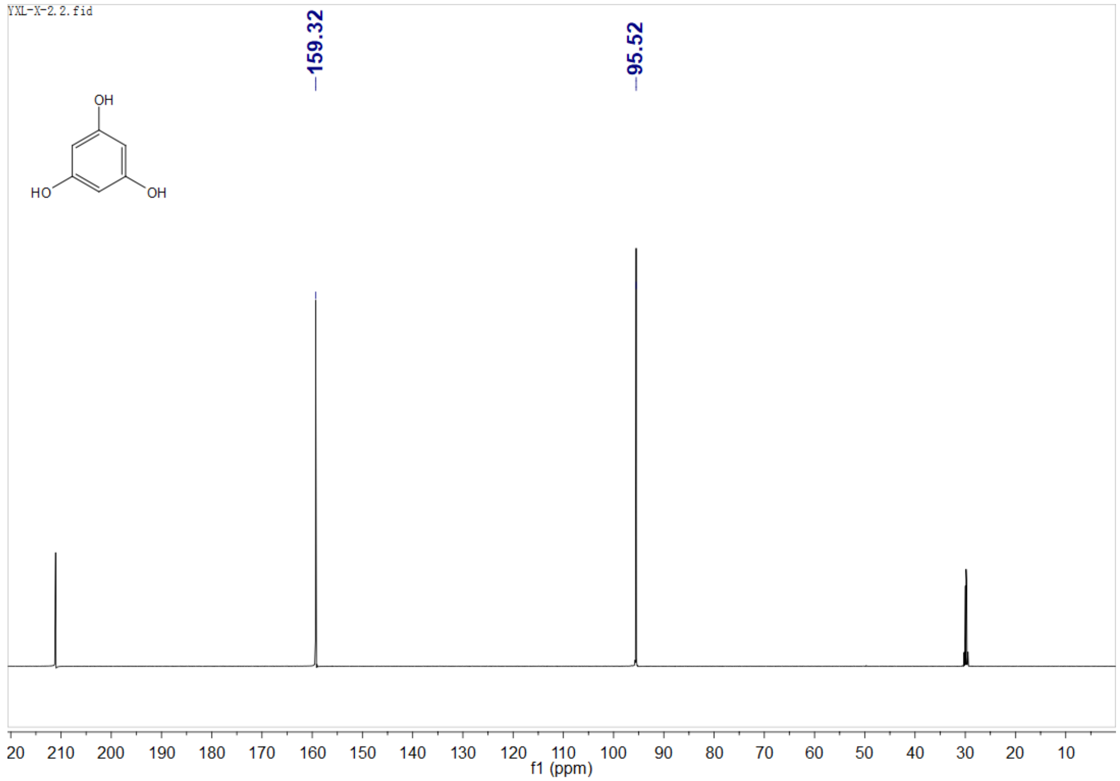

Supplementary Figure 13. ^{13}C NMR data for compound **2** (150 MHz, CD_3COCD_3)

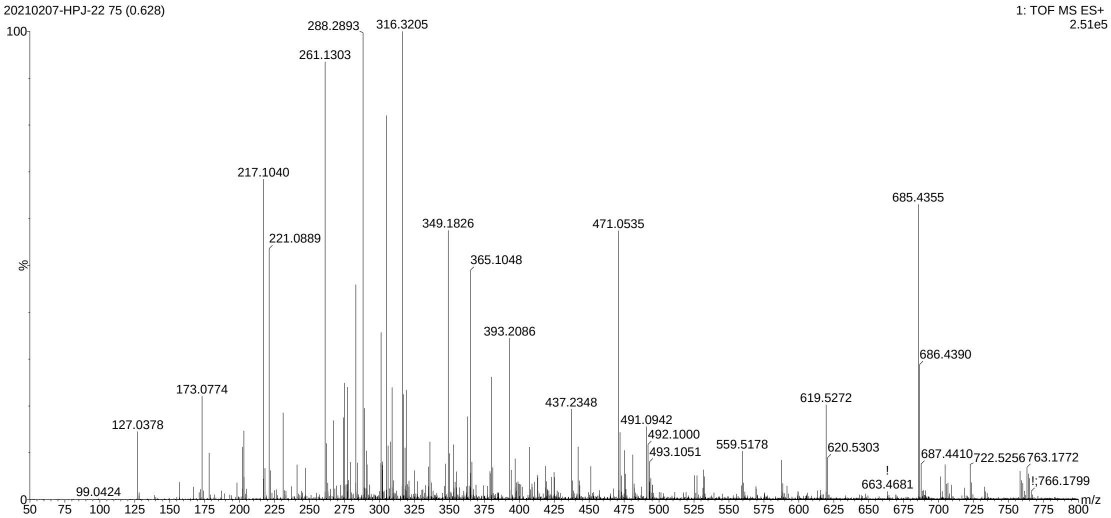

Supplementary Figure 14. HRMS data for compound **2**

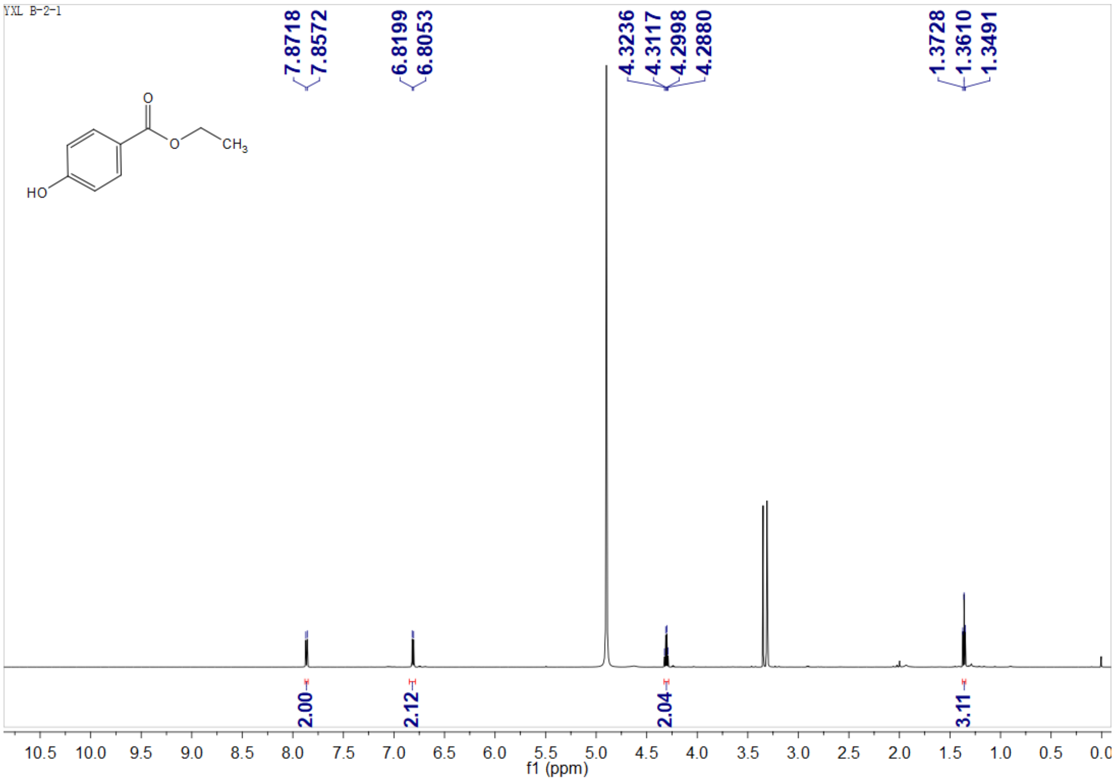

Supplementary Figure 15. ^{1}H NMR data for compound **3** (600 MHz, CD_3OD)

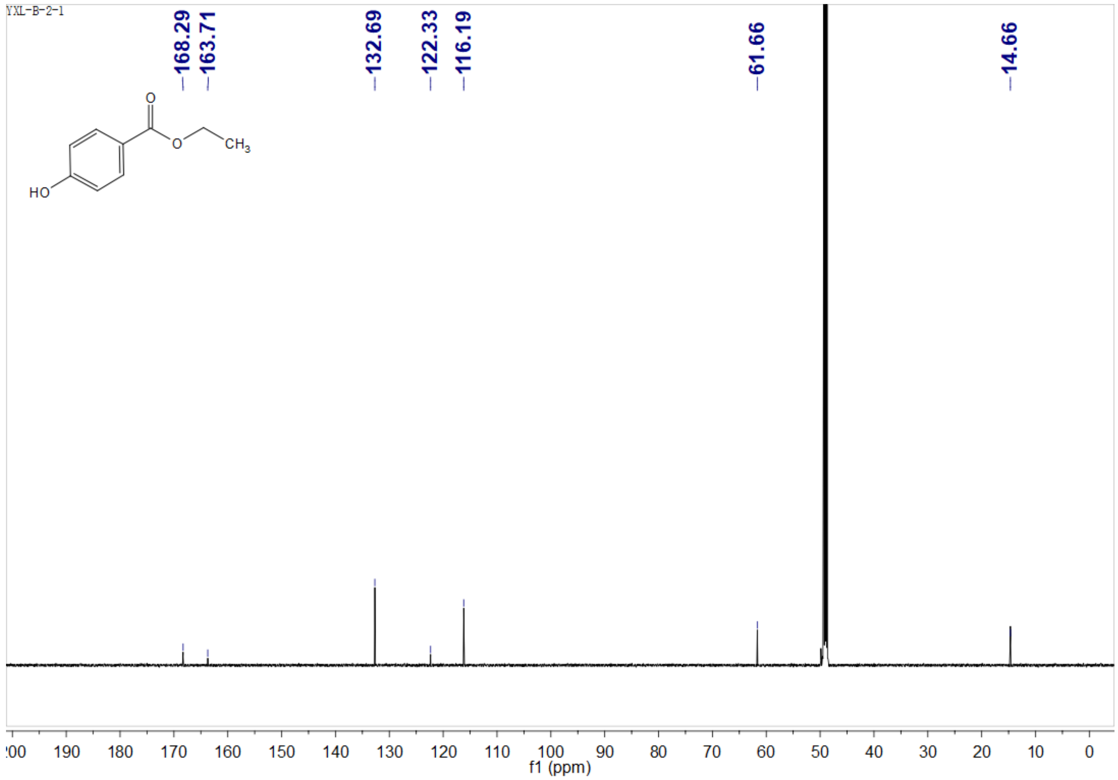

Supplementary Figure S 16. ^{13}C NMR data for compound **3** (150MHz, CD_3OD)

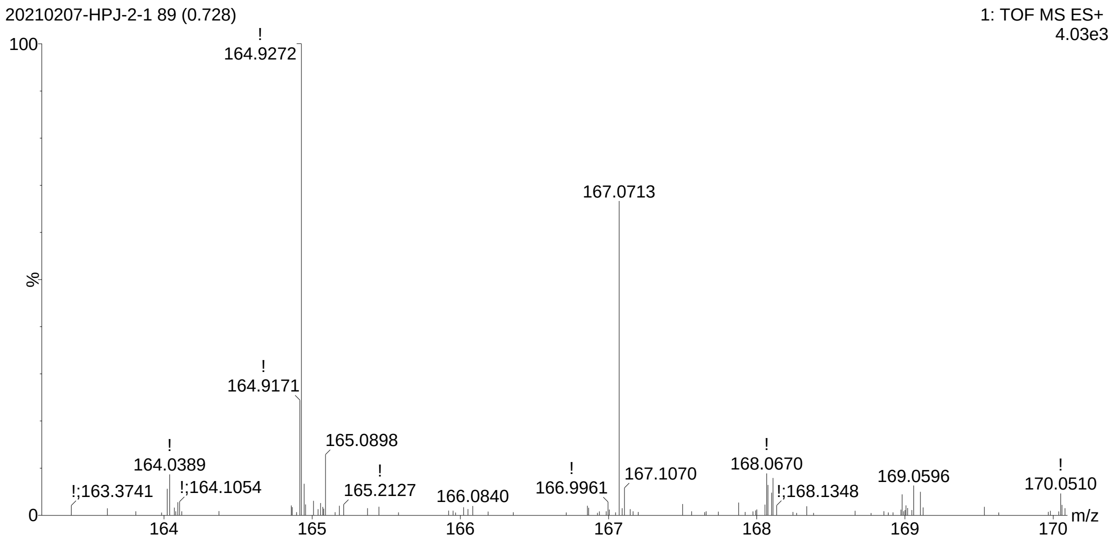

Supplementary Figure 17. HRMS data for compound **3**

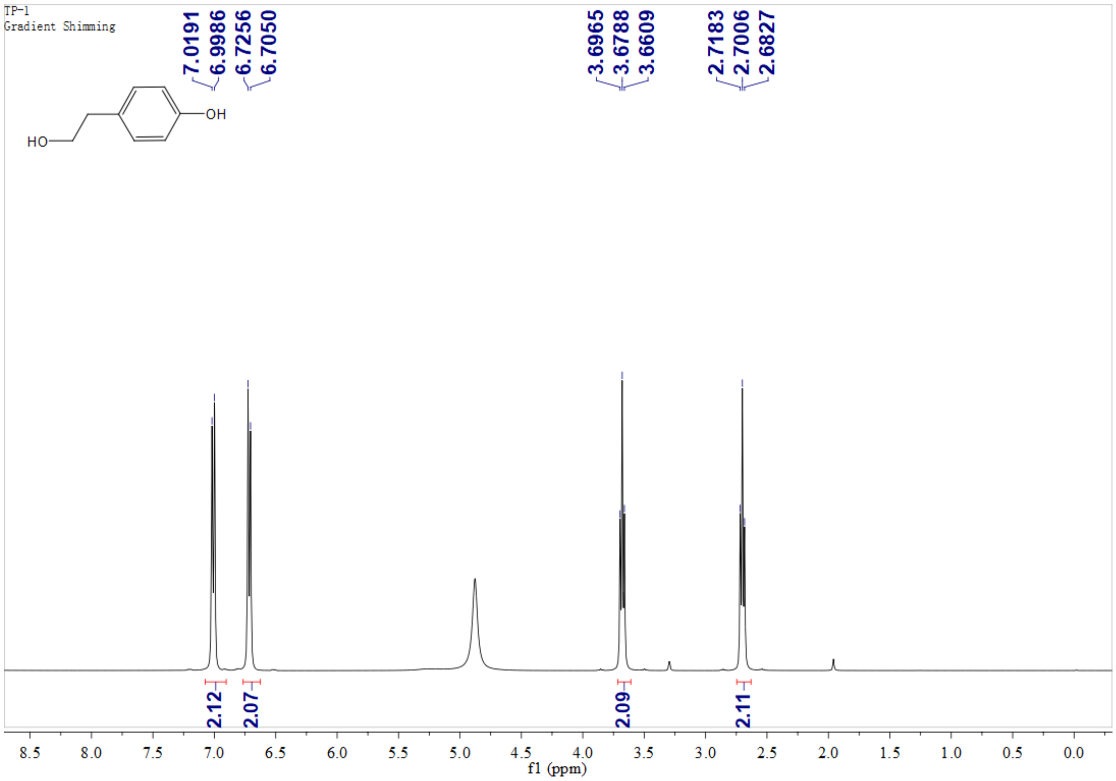

Supplementary Figure 18. ^{1}H NMR data for compound **4** (400 MHz, CD_3OD)

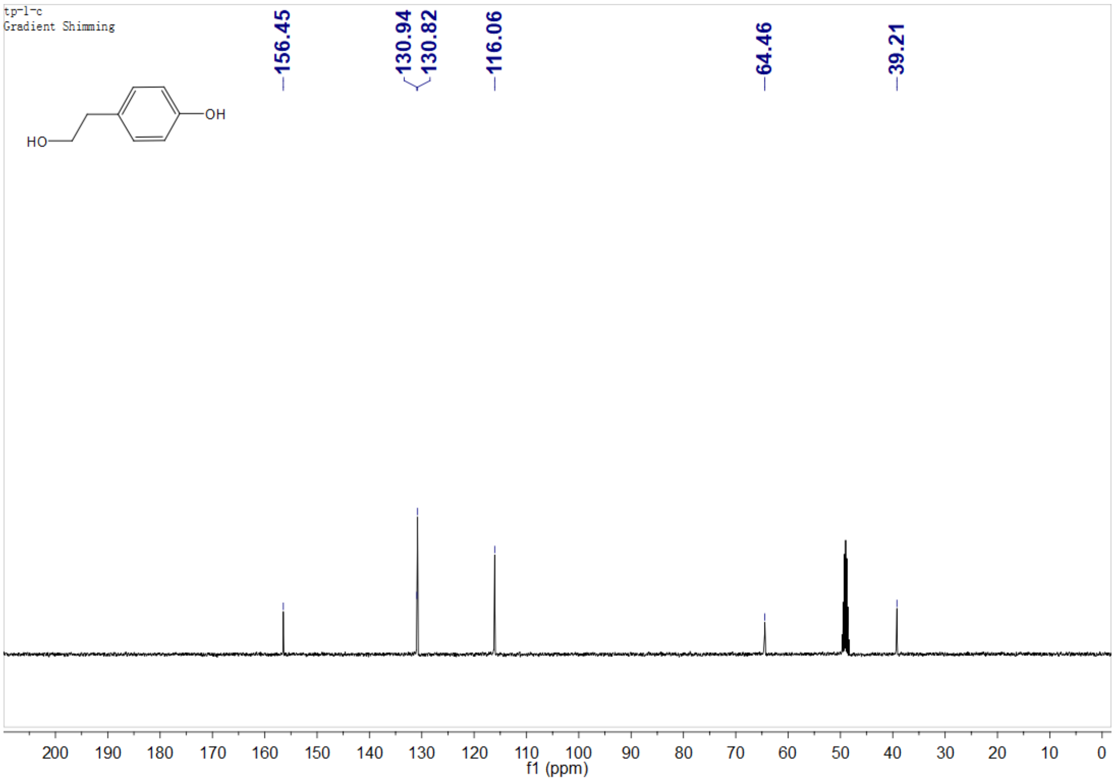

Supplementary Figure S 19. ^{13}C NMR data for compound **4** (100 MHz, CD_3OD)

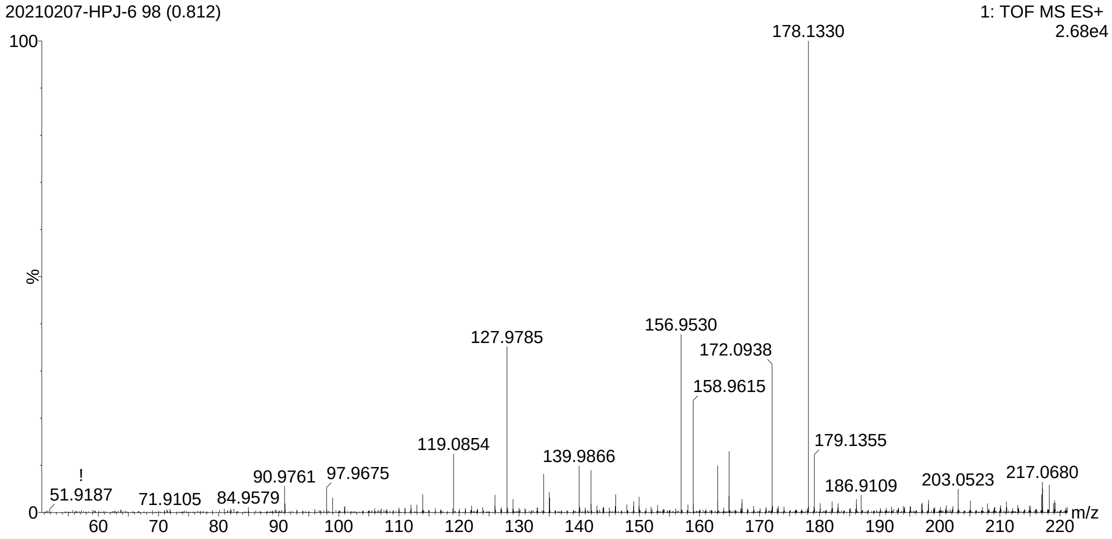

Supplementary Figure 20. HRMS data for compound **4**

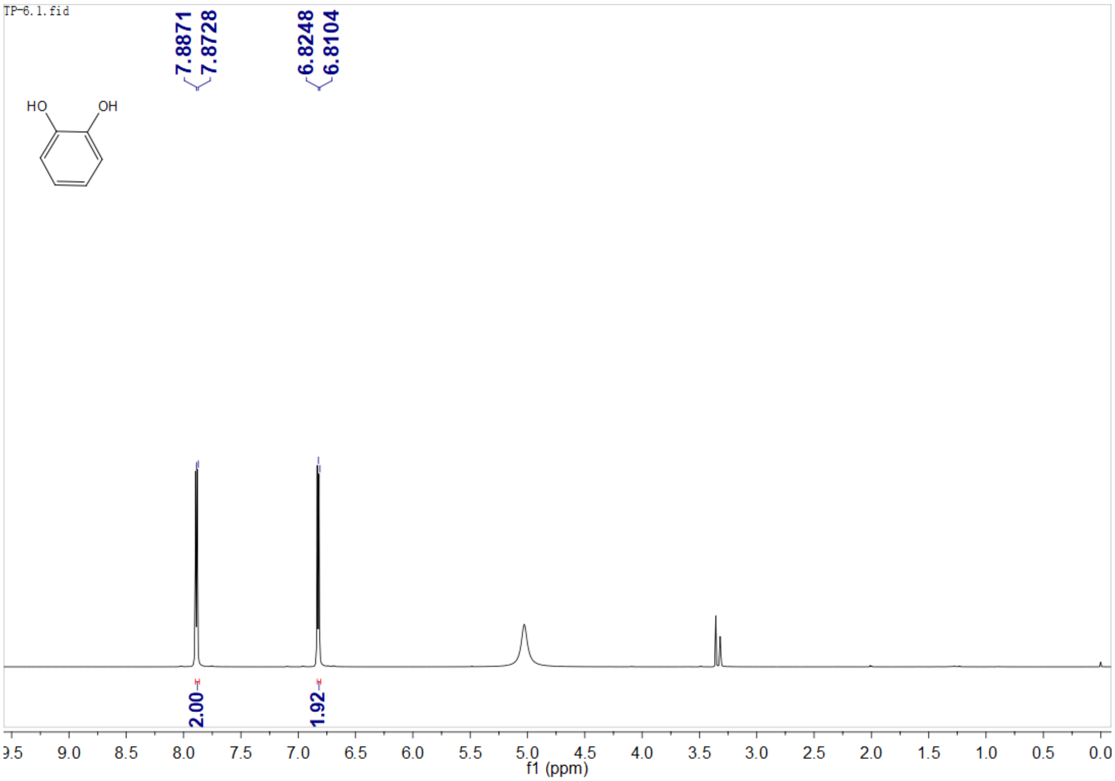

Supplementary Figure 21. ^{1}H NMR data for compound **5** (400 MHz, CD_3OD)

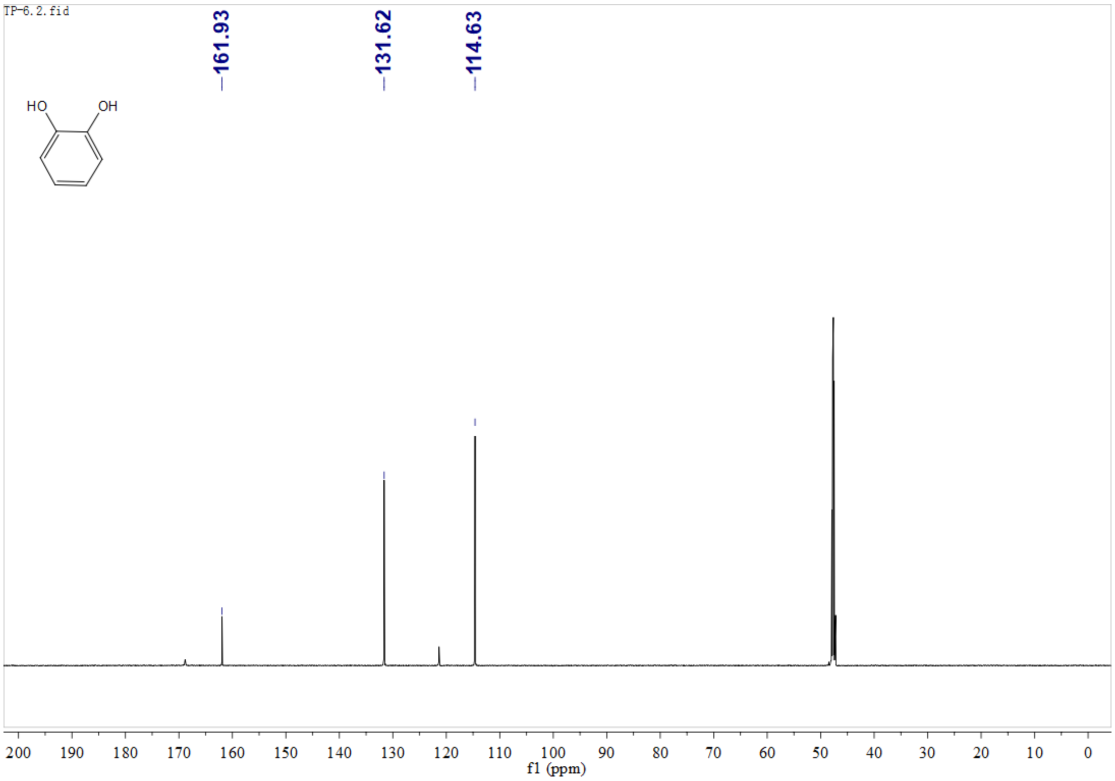

Supplementary Figure 22. ^{13}C NMR data for compound **5** (100 MHz, CD_3OD)

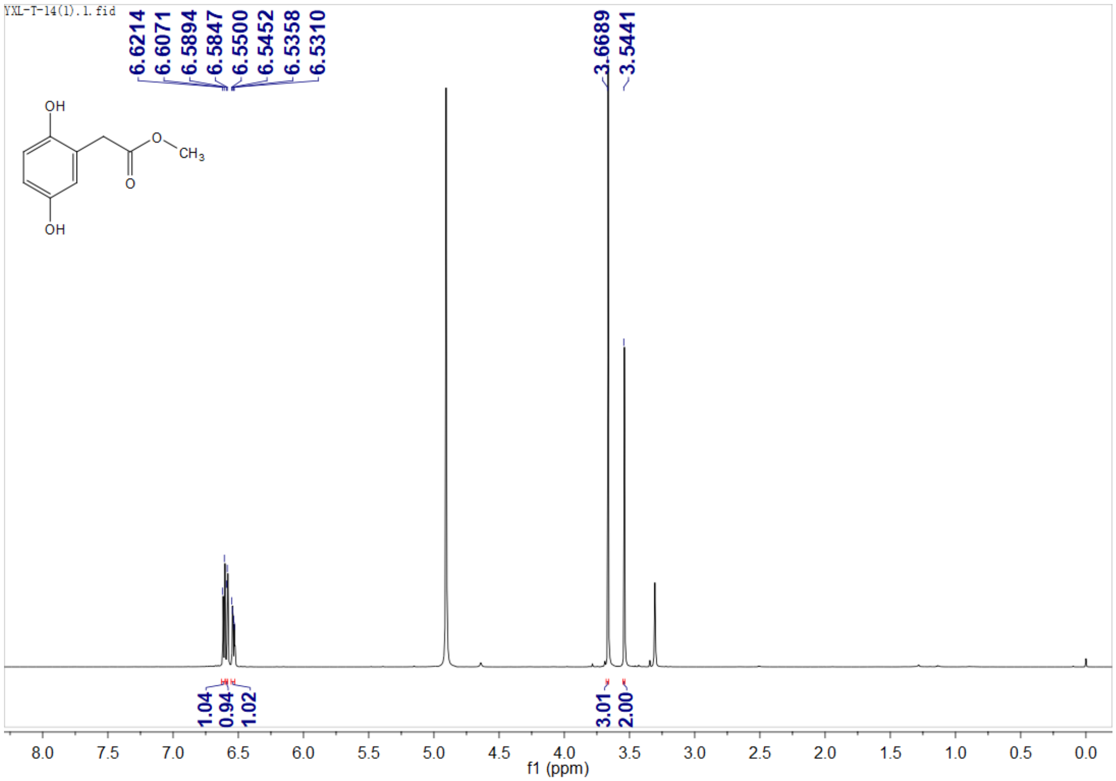

Supplementary Figure 23. ^{1}H NMR data for compound **6** (600 MHz, CD_3OD)

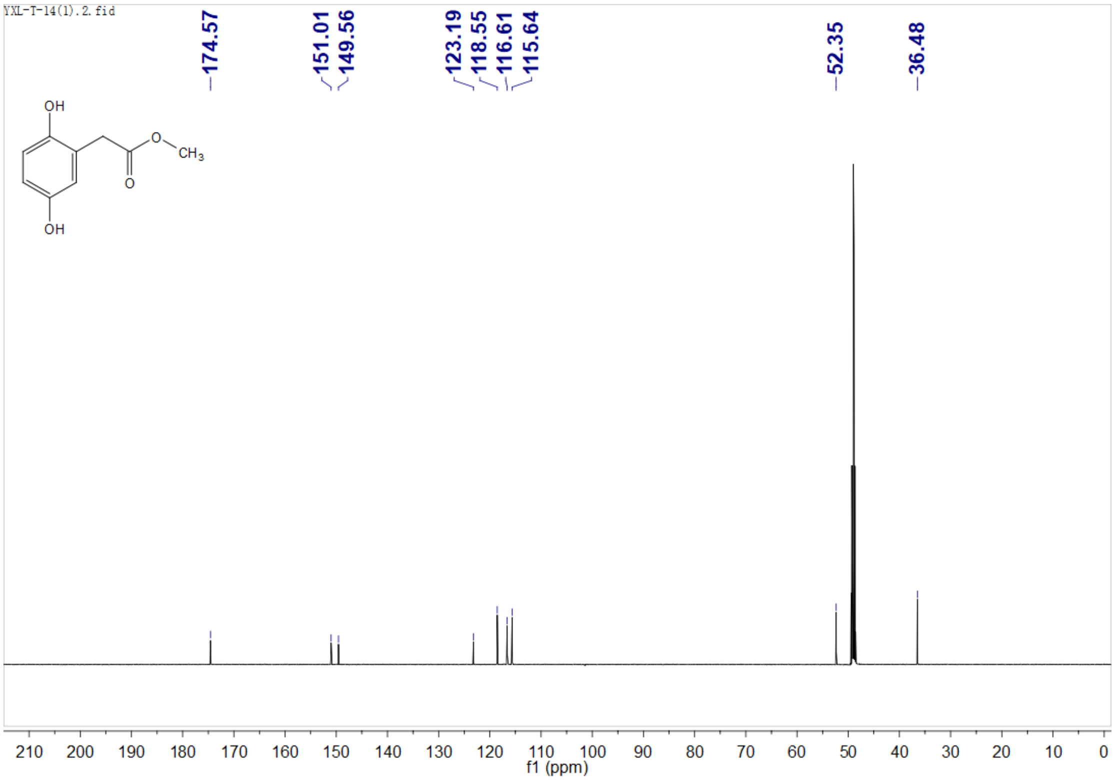

Supplementary Figure 24. ^{13}C NMR data for compound **6** (150 MHz, CD_3OD)

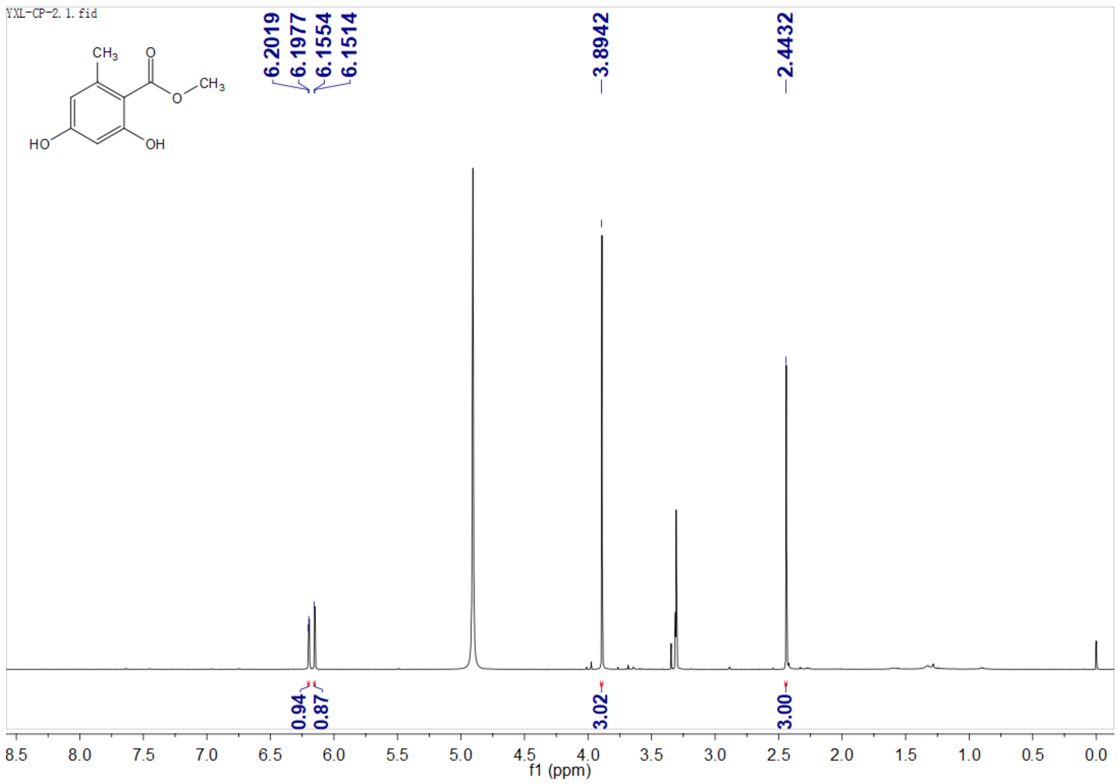

Supplementary Figure 25. ^{1}H NMR data for compound **7** (600 MHz, CD_3OD)

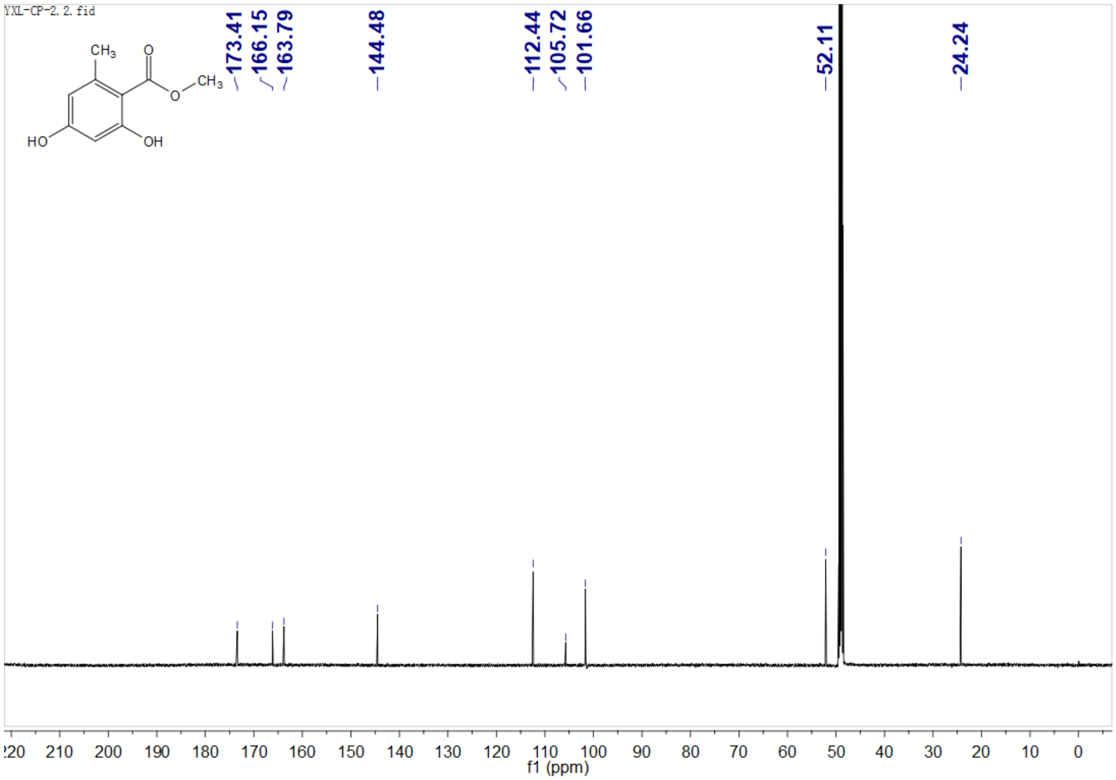

Supplementary Figure 26. ^{13}C NMR data for compound **7** (150 MHz, CD_3OD)

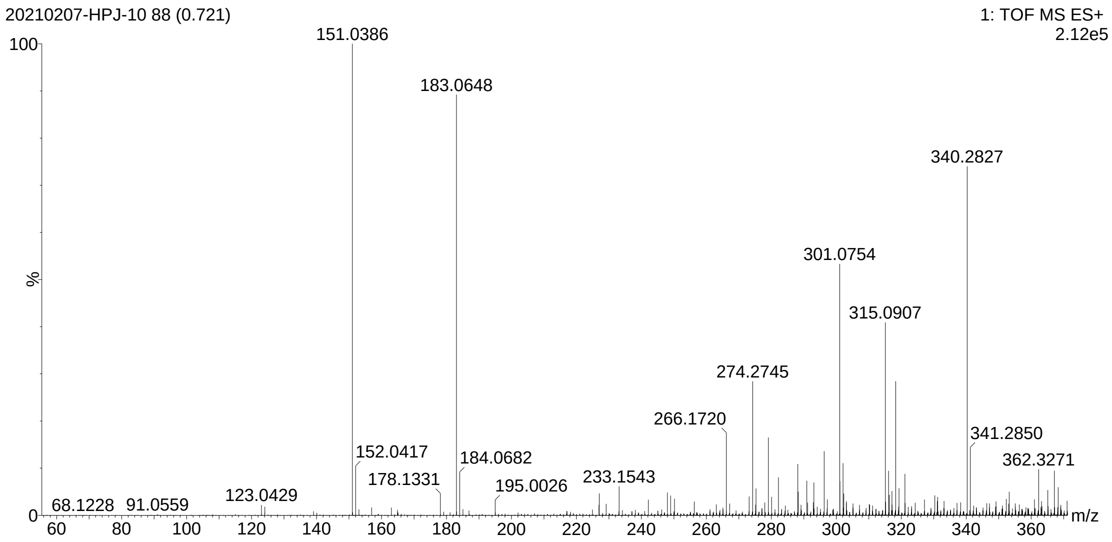

Supplementary Figure 27. HRMS data for compound **7**

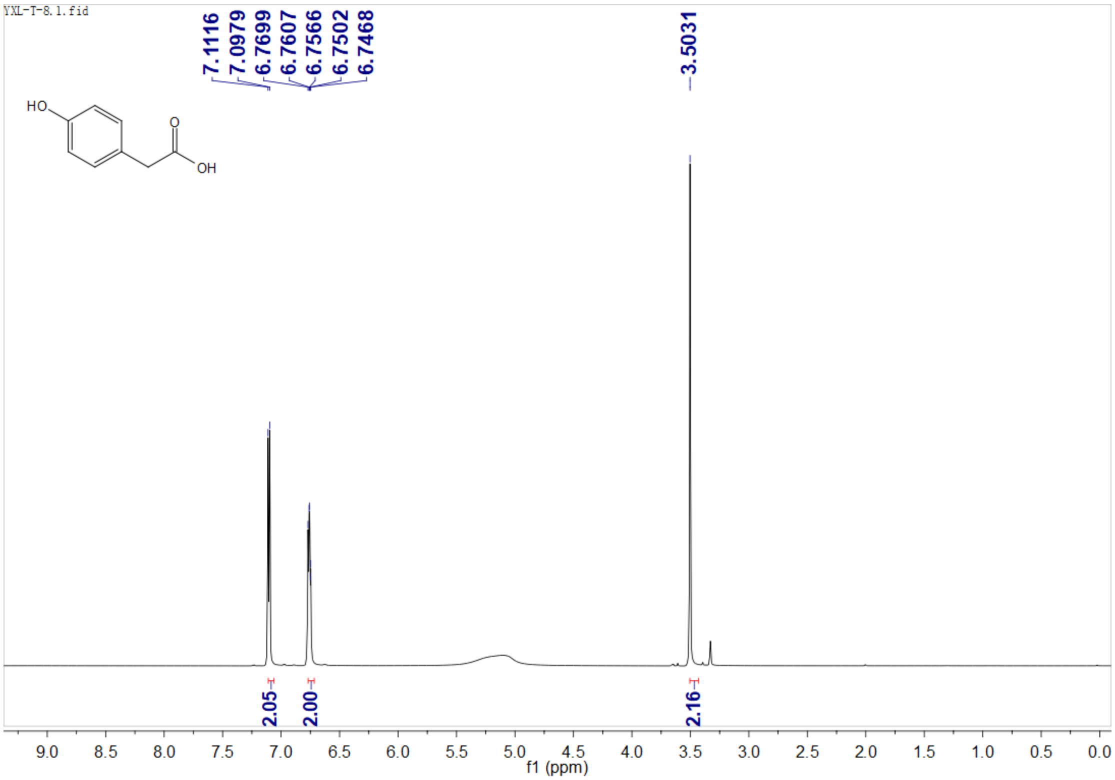

Supplementary Figure 28. ^{1}H NMR data for compound **8** (600 MHz, CD_3OD)

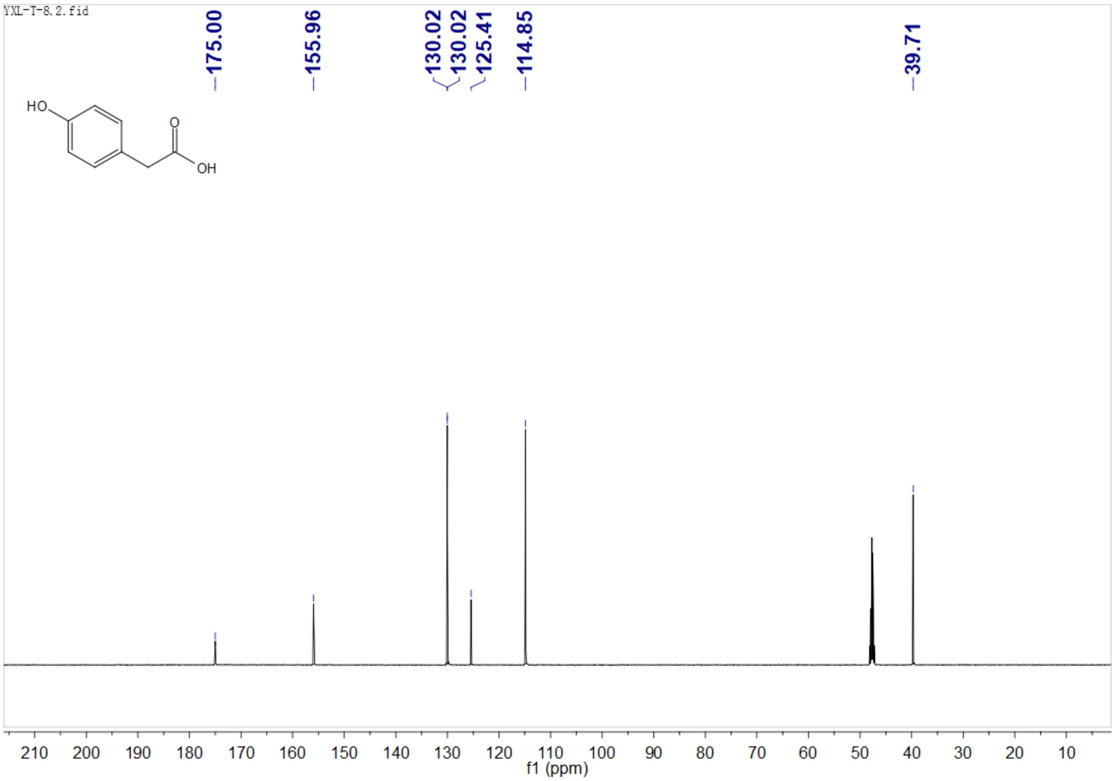

Supplementary Figure 29. ^{13}C NMR data for compound **8** (150 MHz, CD_3OD)

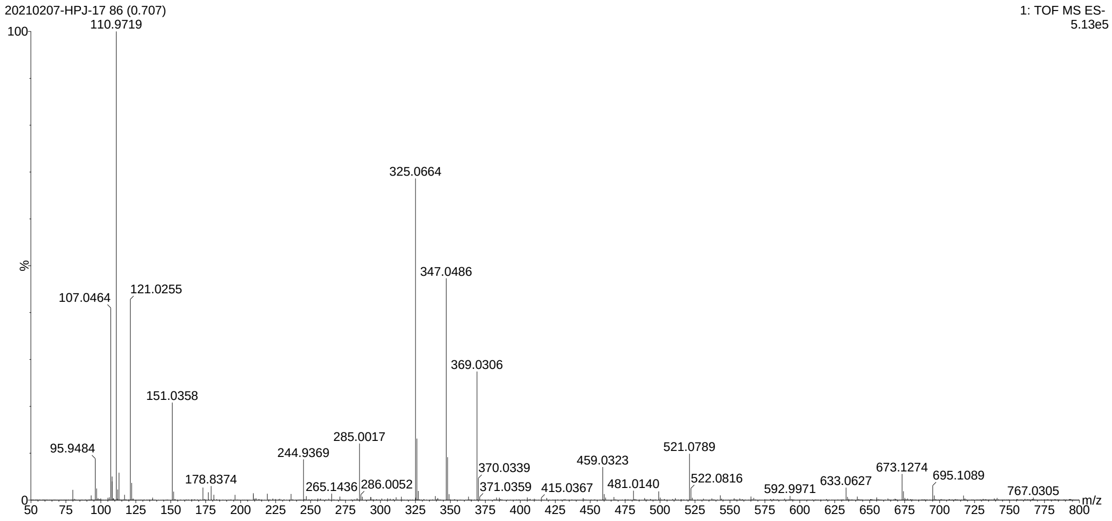

Supplementary Figure 30. HRMS data for compound **8**

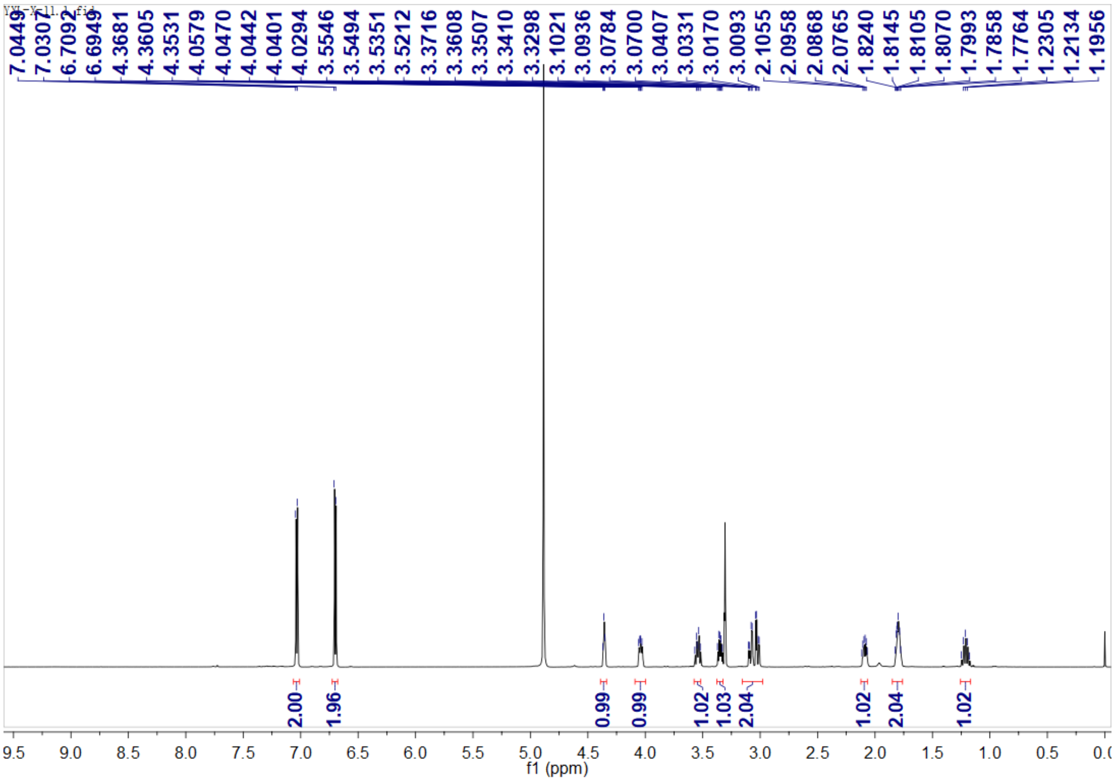

Supplementary Figure 31. ^{1}H NMR data for compound **9** (600 MHz, CD_3OD)

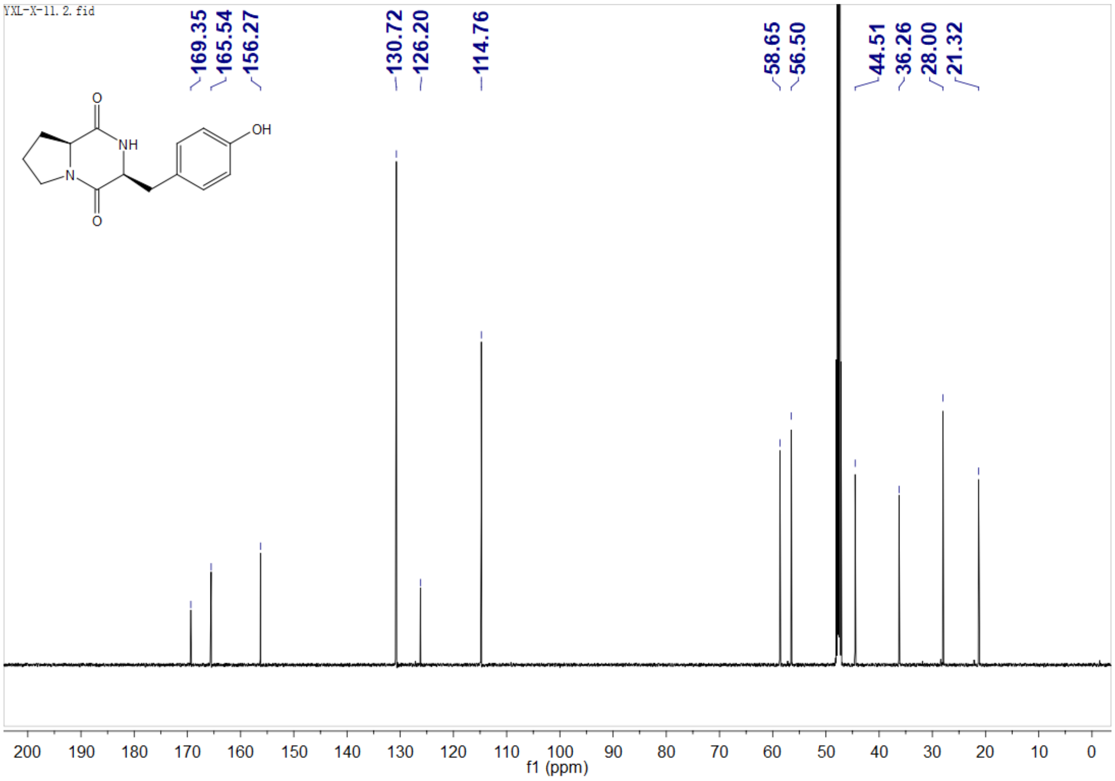

Supplementary Figure 32. ^{13}C NMR data for compound **9** (150 MHz, CD_3OD)

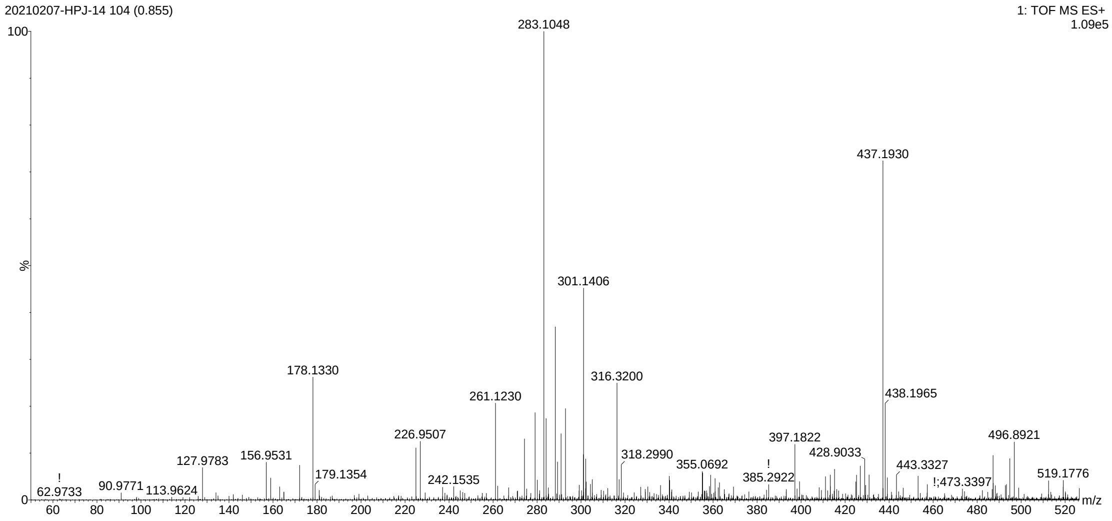

Supplementary Figure 33. HRMS data for compound **9**

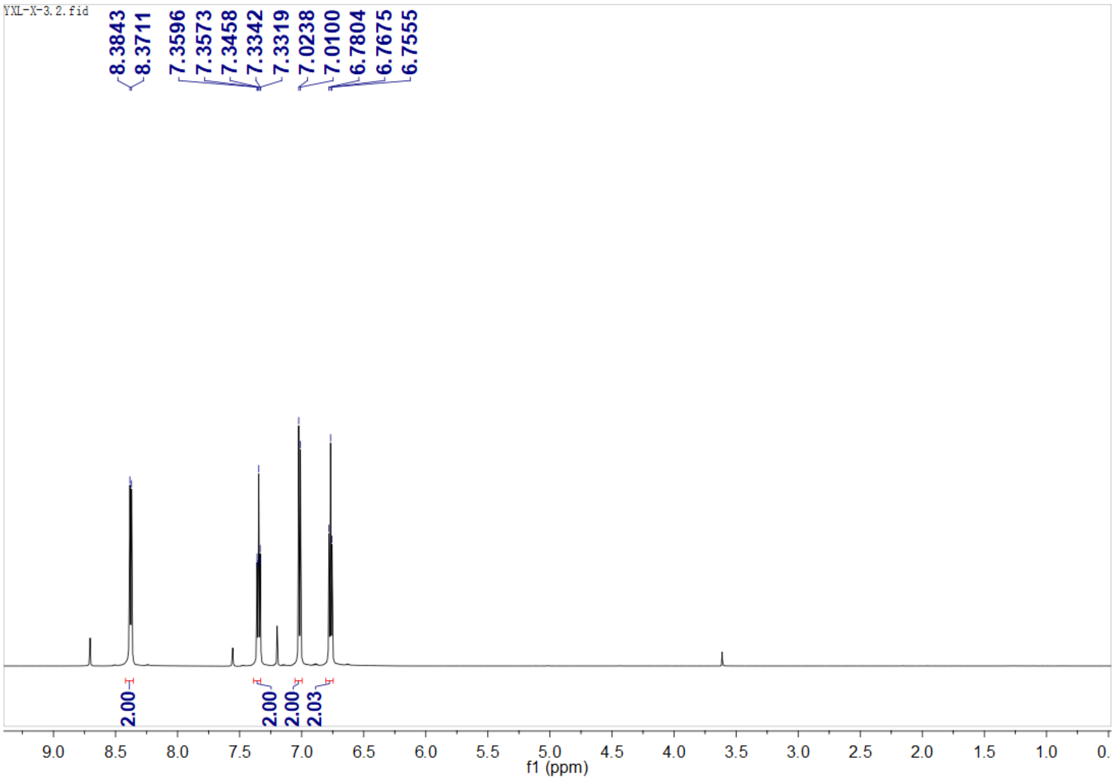

Supplementary Figure 34. ^{1}H NMR data for compound **10** (600 MHz, C_5D_5N)

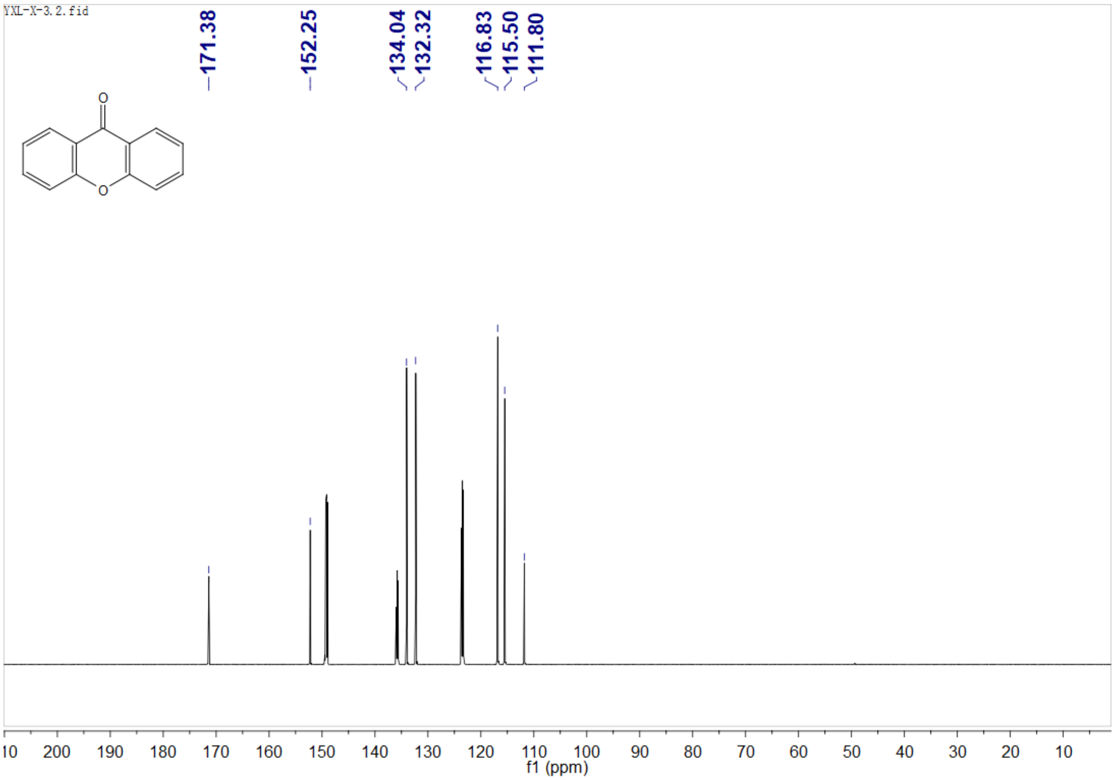

Supplementary Figure 35. ^{13}C NMR data for compound **10** (10 MHz, C_5D_5N)

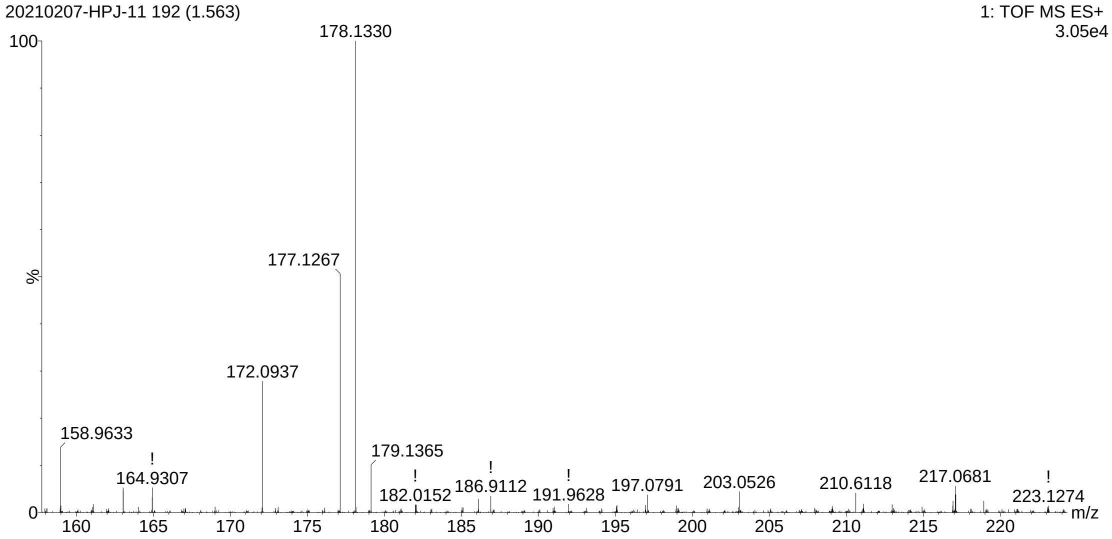

Supplementary Figure 36. HRMS data for compound **10**

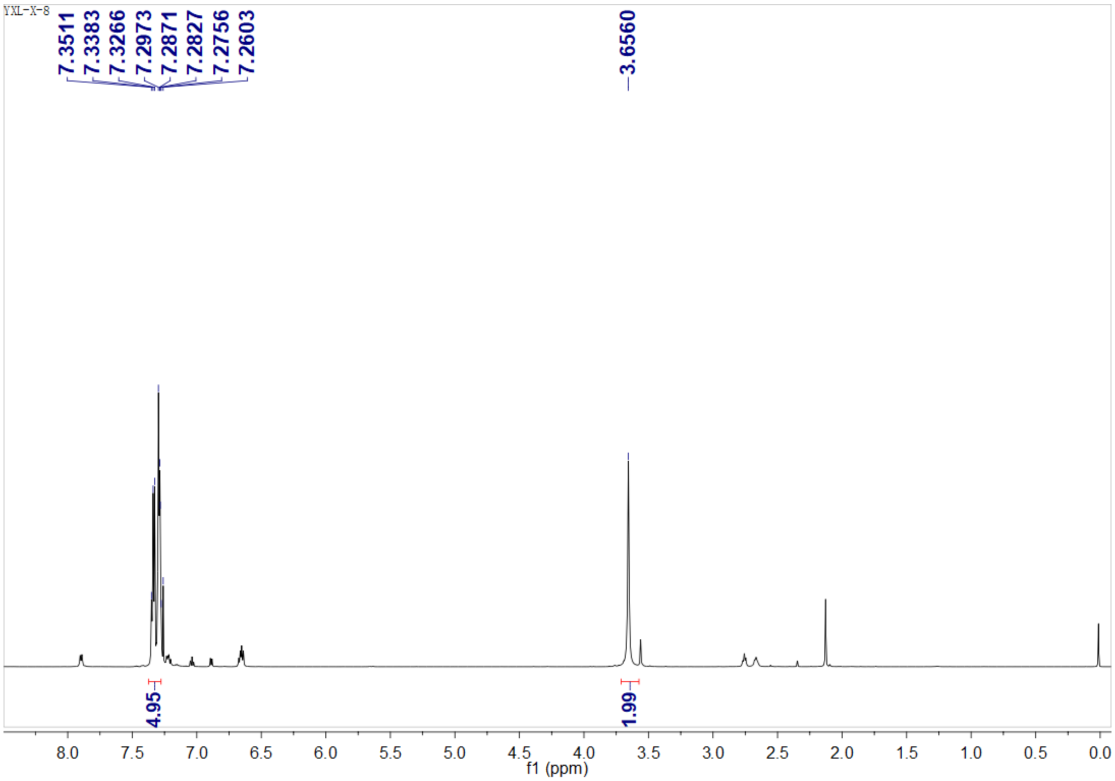

Supplementary Figure 37. ^{1}H NMR data for compound **11** (600 MHz, $CDCl_3$)

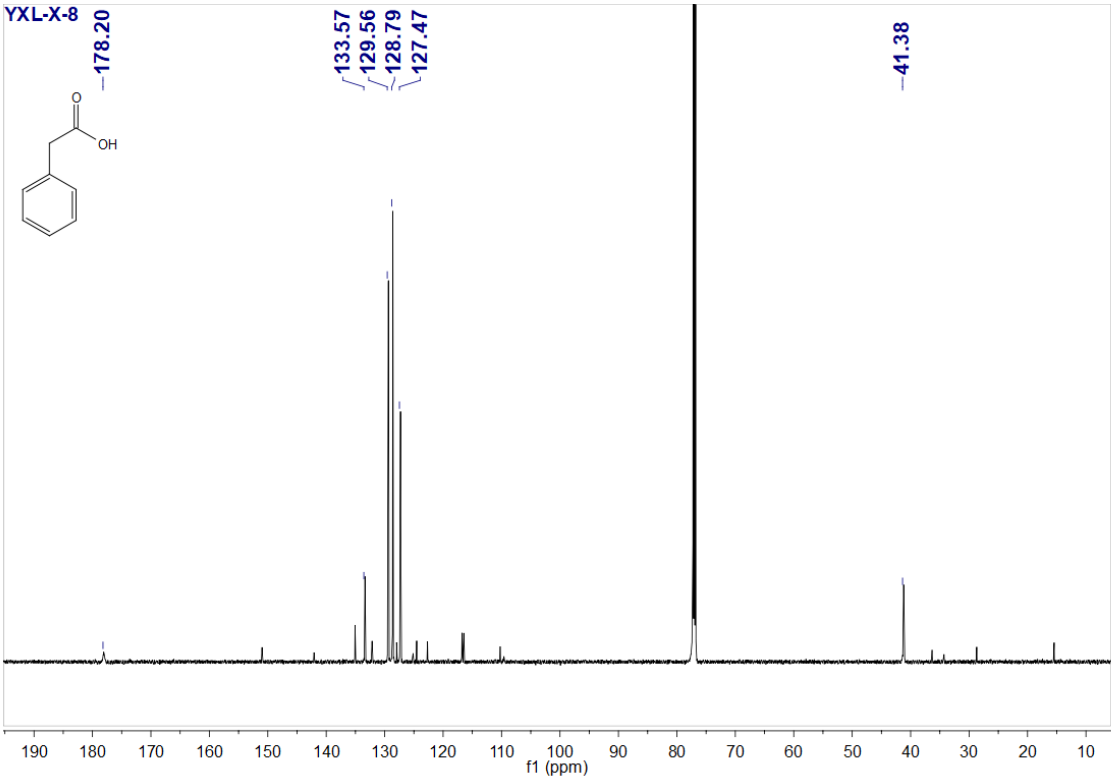

Supplementary Figure 38. ^{13}C NMR data for compound **11** (150 MHz, $CDCl_3$)

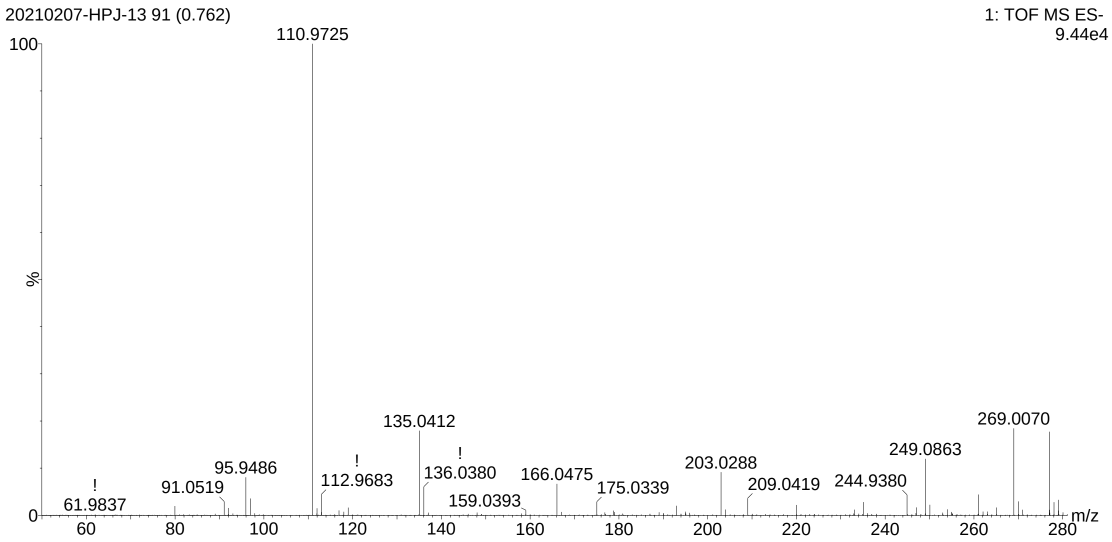

Supplementary Figure 39. HRMS data for compound **11**

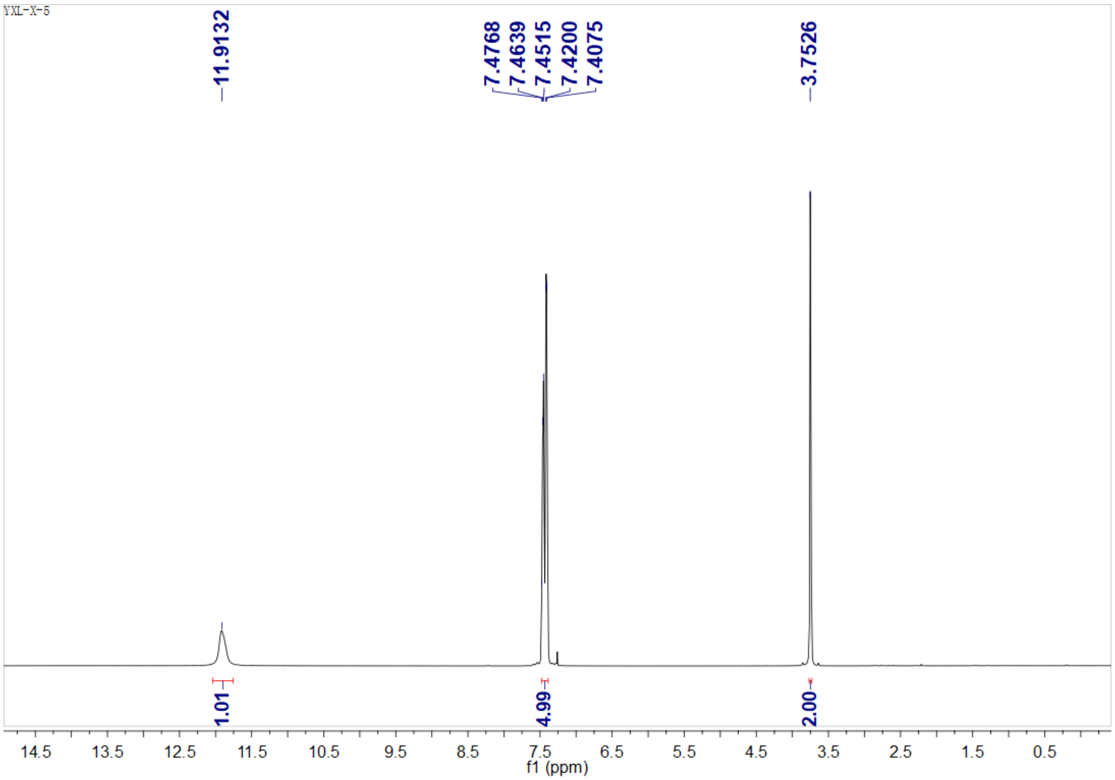

Supplementary Figure 40. ^{1}H NMR data for compound **12** (400 MHz, $CDCl_3$)

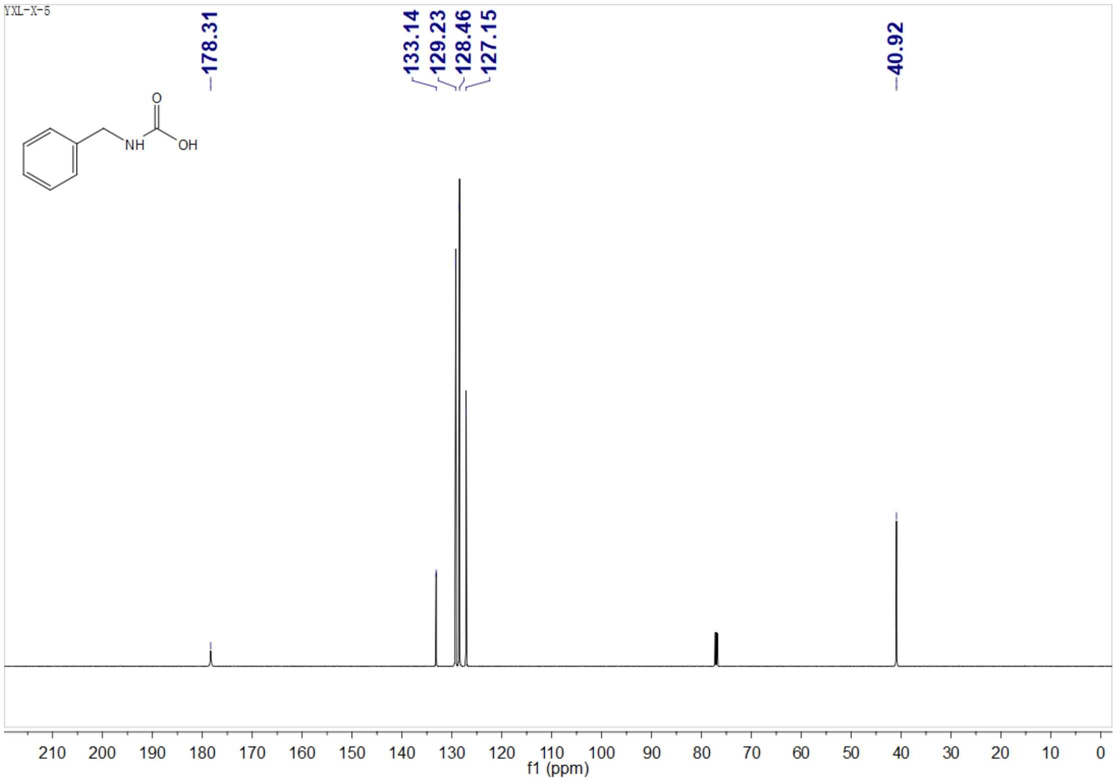

Supplementary Figure 41. ^{13}C NMR data for compound **12** (150 MHz, $CDCl_3$)

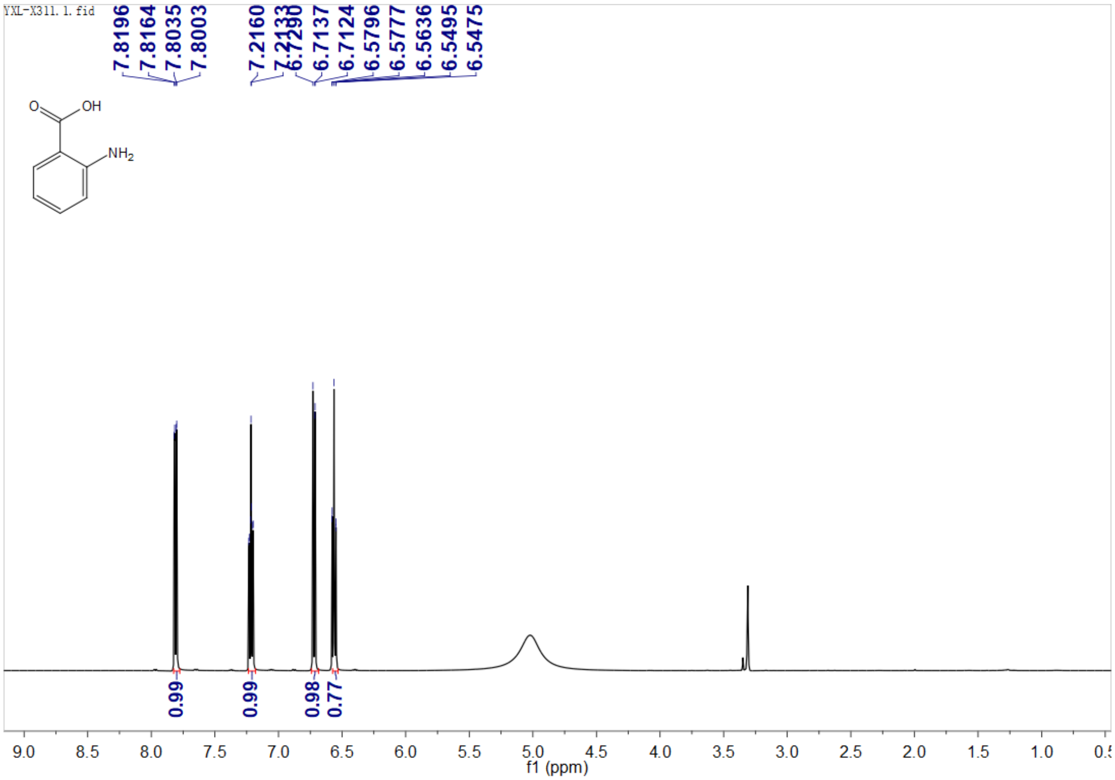

Supplementary Figure 42. ^{1}H NMR data for compound **13** (600 MHz, CD_3OD)

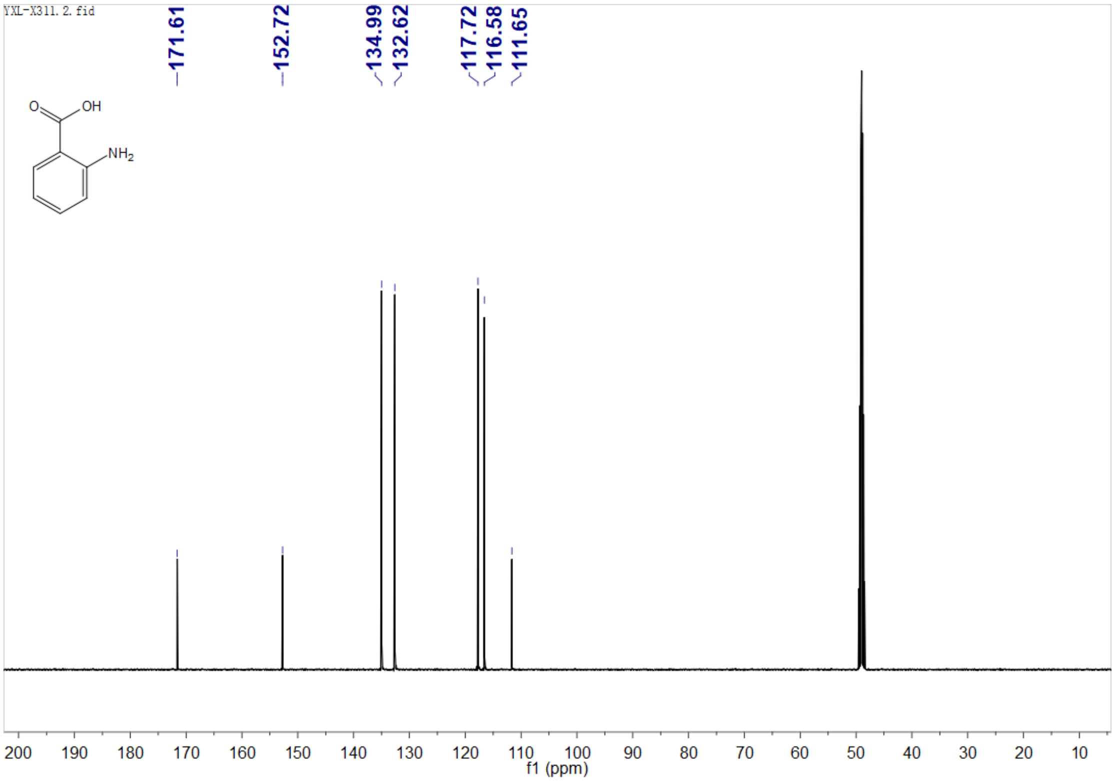

Supplementary Figure 43. ^{13}C NMR data for compound **13** (150 MHz, CD_3OD)

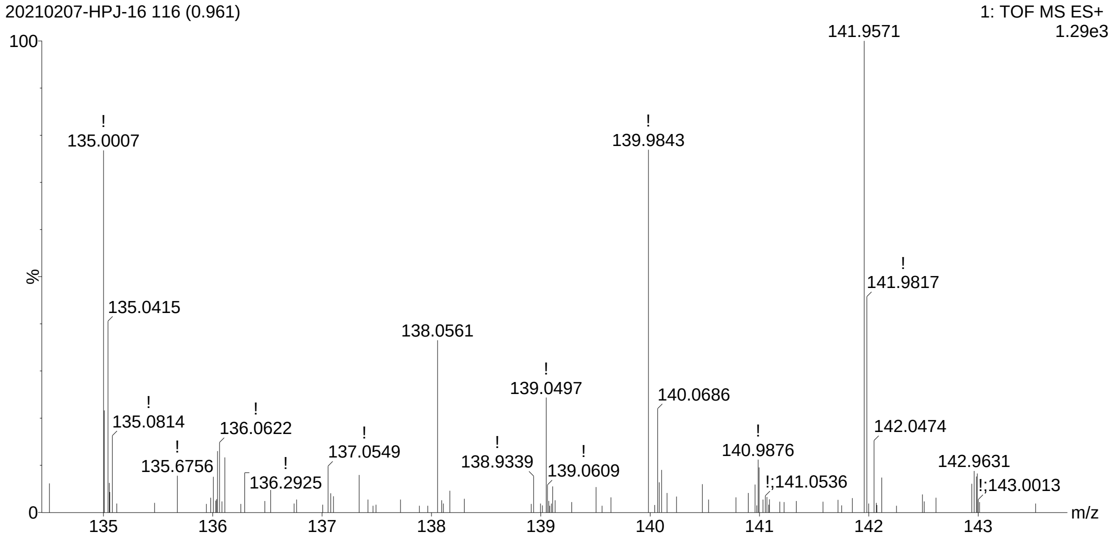

Supplementary Figure 44. HRMS data for compound **13**

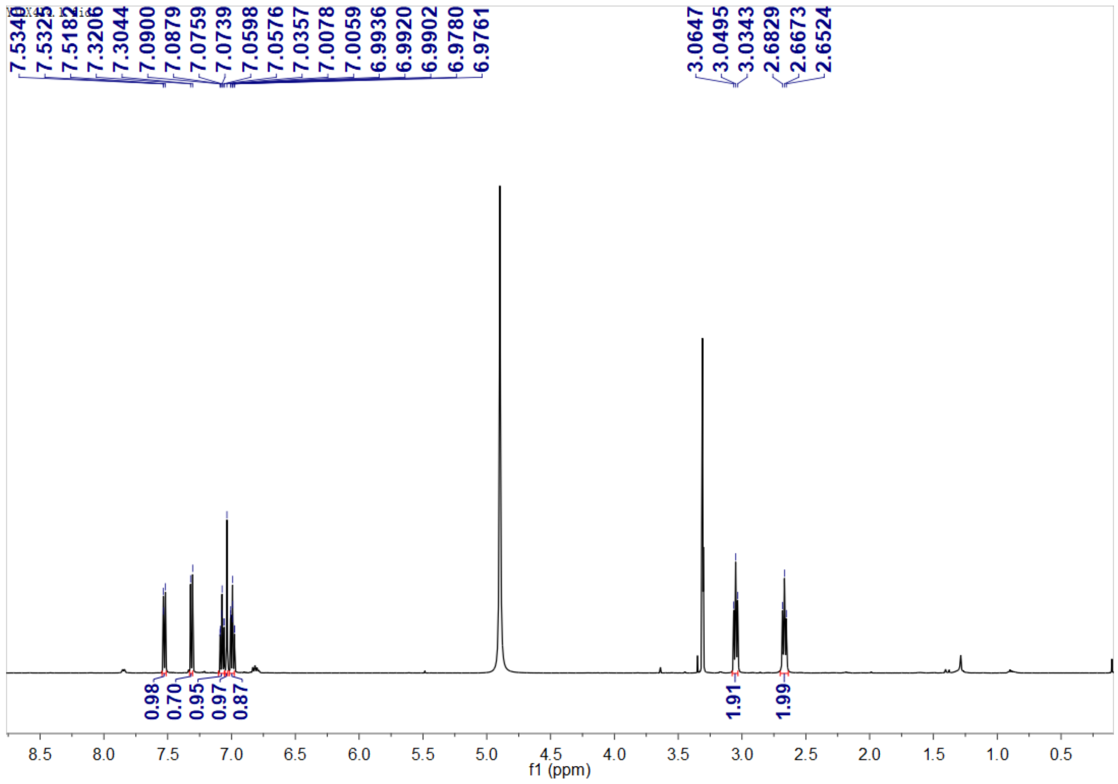

Supplementary Figure 45. ^{1}H NMR data for compound **14** (600 MHz, CD_3OD)

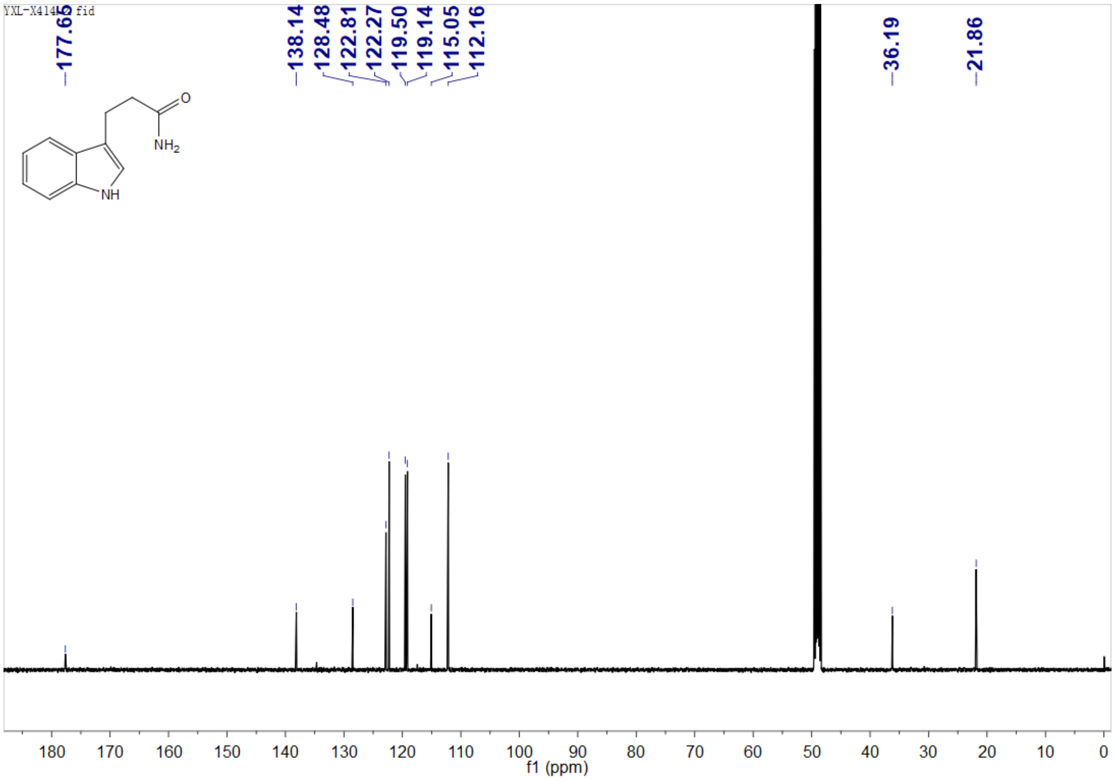

Supplementary Figure 46. ^{13}C NMR data for compound **14** (150 MHz, CD_3OD)

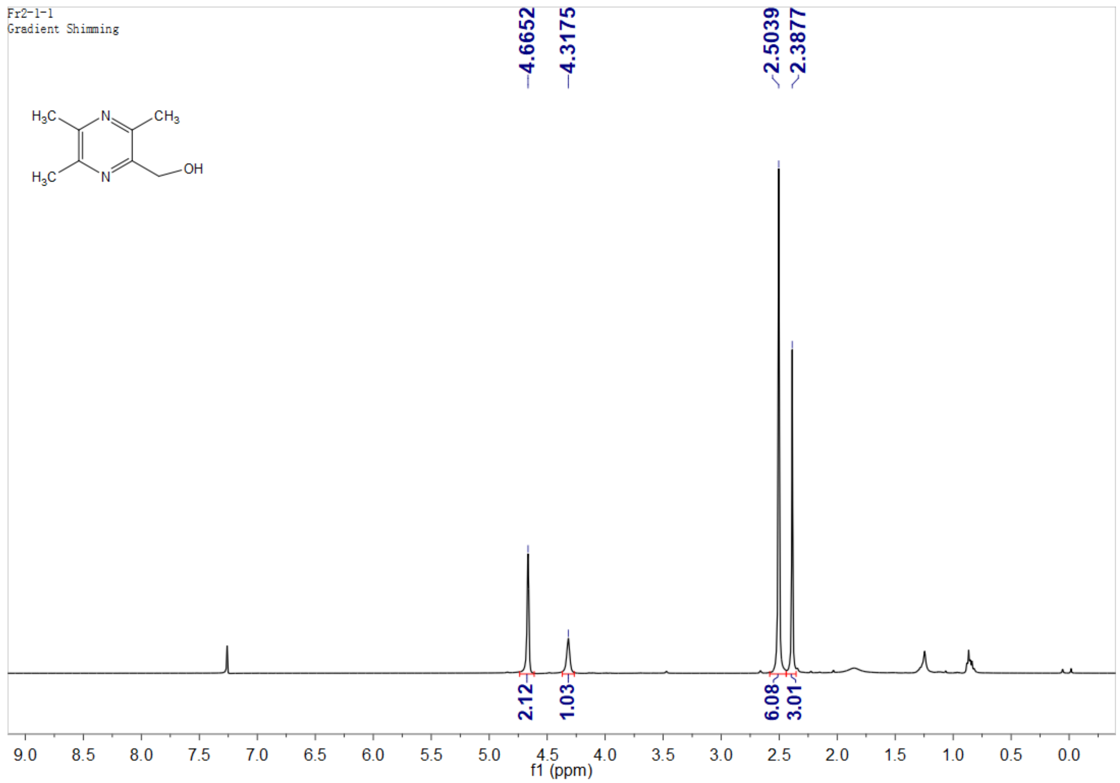

Supplementary Figure 47. ^1H NMR data for compound **15** (600 MHz, $CDCl_3$)

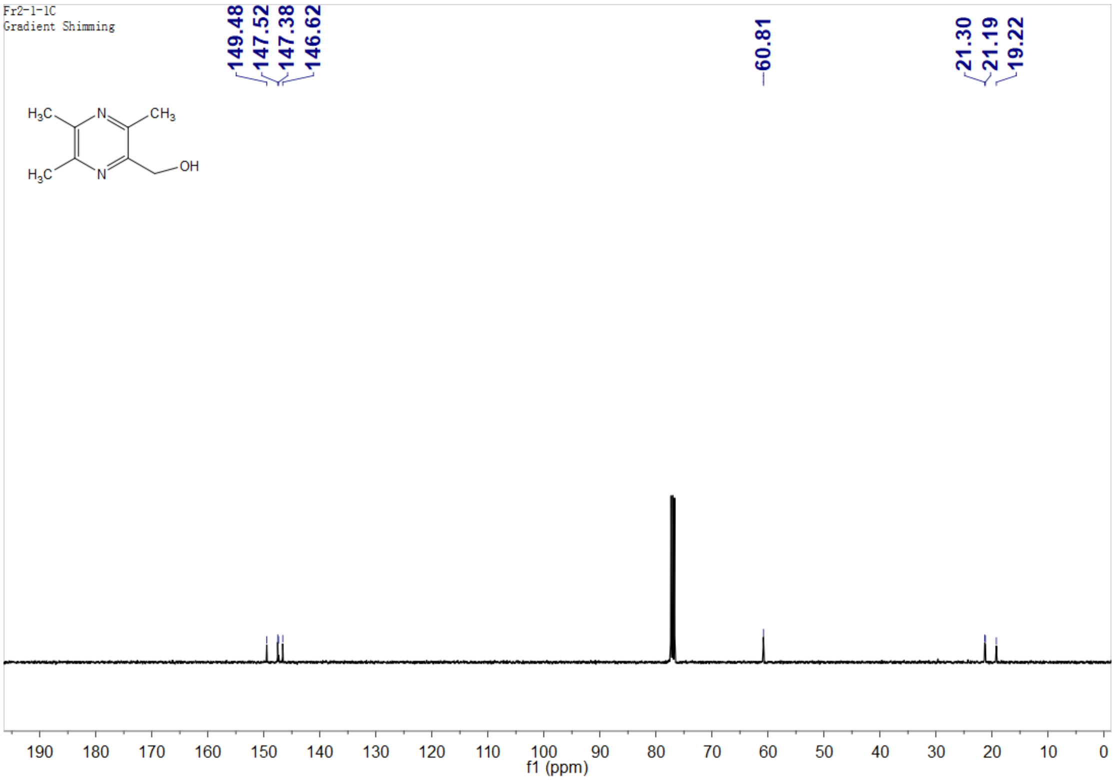

Supplementary Figure 48. ^{13}C NMR data for compound **15** (150 MHz, $CDCl_3$)

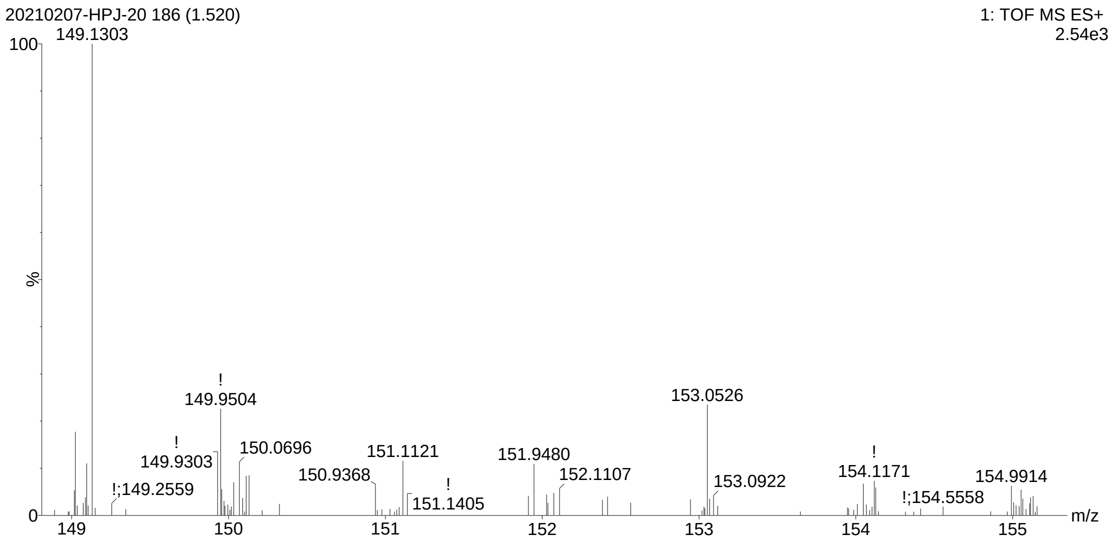

Supplementary Figure 49. HRMS data for compound **15**

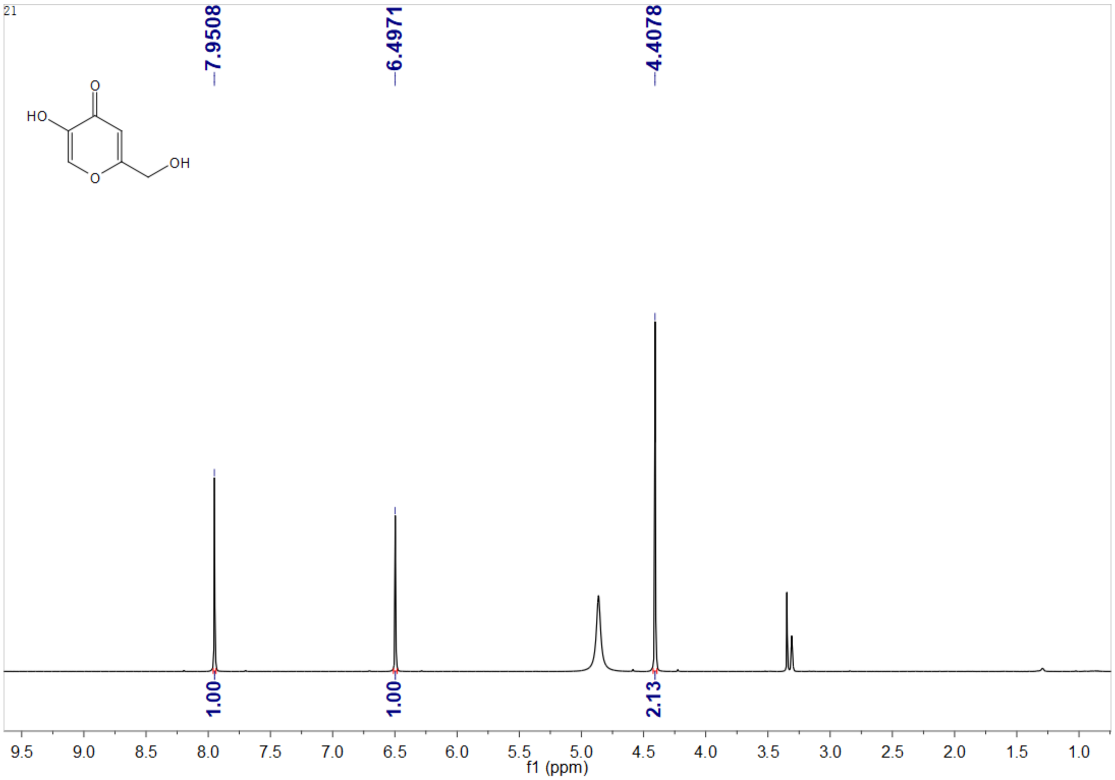

Supplementary Figure 50. ^{1}H NMR data for compound **16** (600 MHz, CD_3OD)

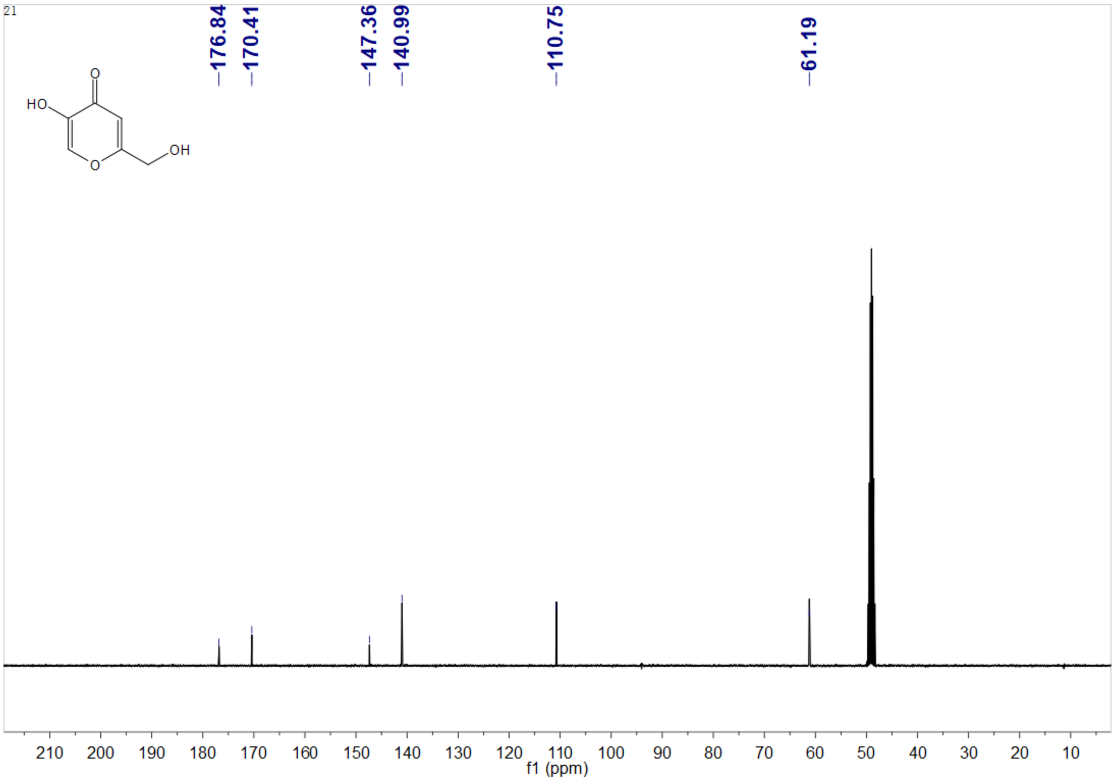

Supplementary Figure 51. ^{13}C NMR data for compound **16** (150 MHz, CD_3OD)

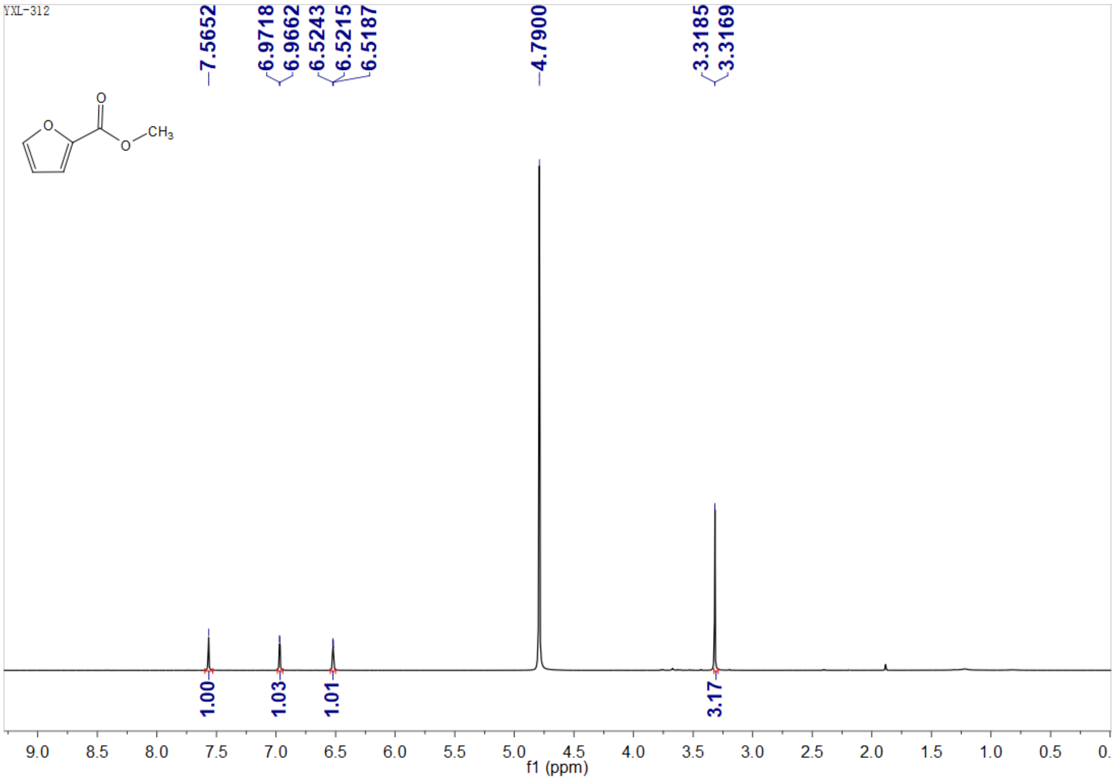

Supplementary Figure 52. ^{1}H NMR data for compound **17** (600 MHz, D_2O)

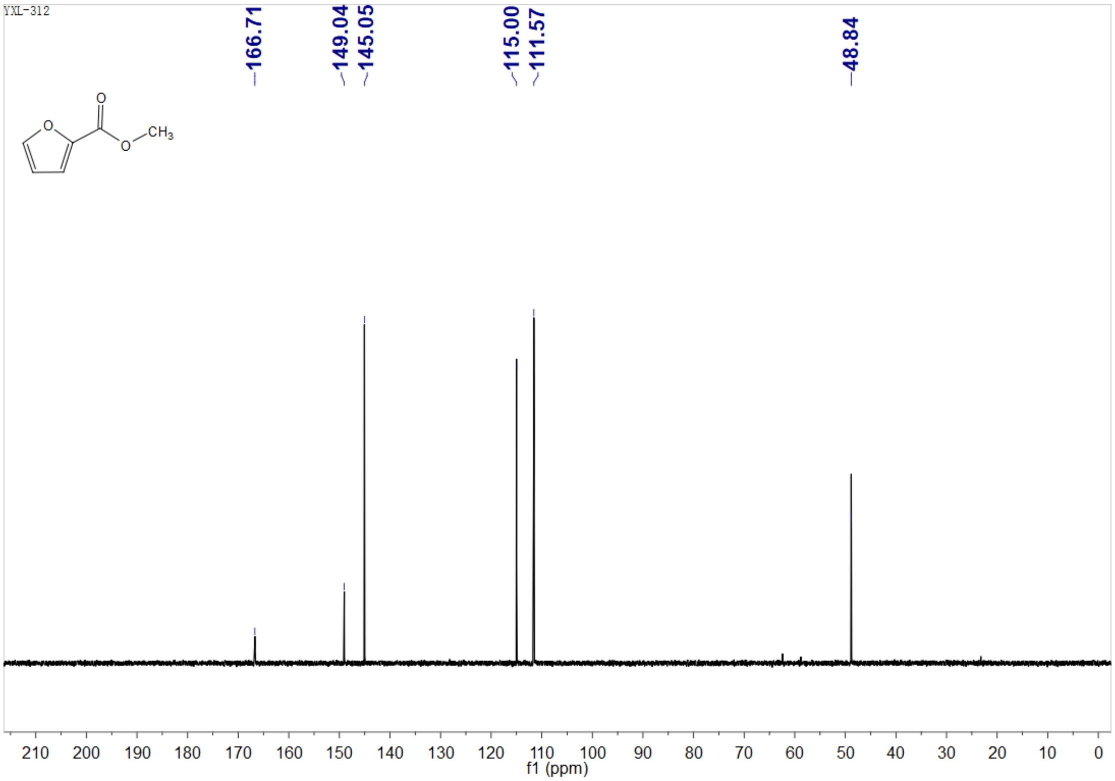

Supplementary Figure 53. ^{13}C NMR data for compound **17** (150 MHz, D_2O)

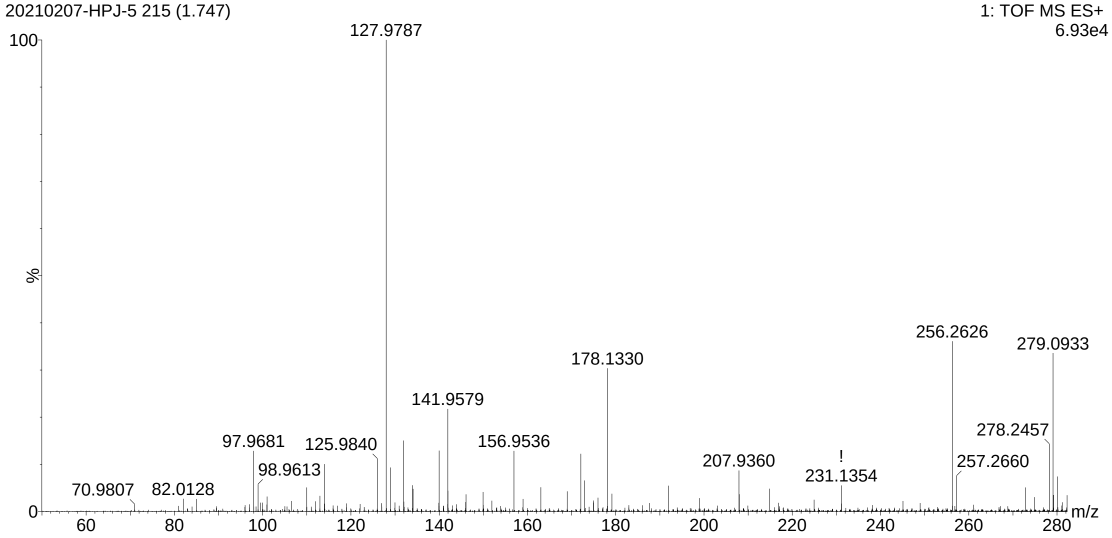

Supplementary Figure 54. HRMS data for compound **17**

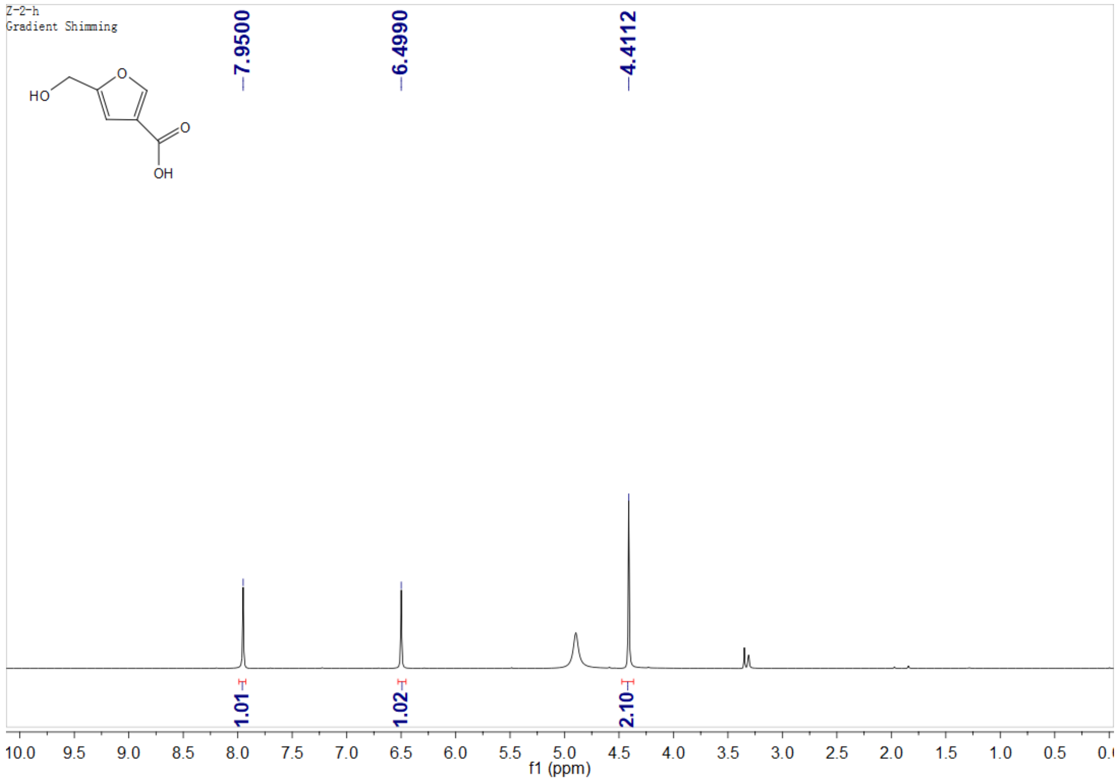

Supplementary Figure 55. ^{1}H NMR data for compound **18** (600 MHz, CD_3OD)

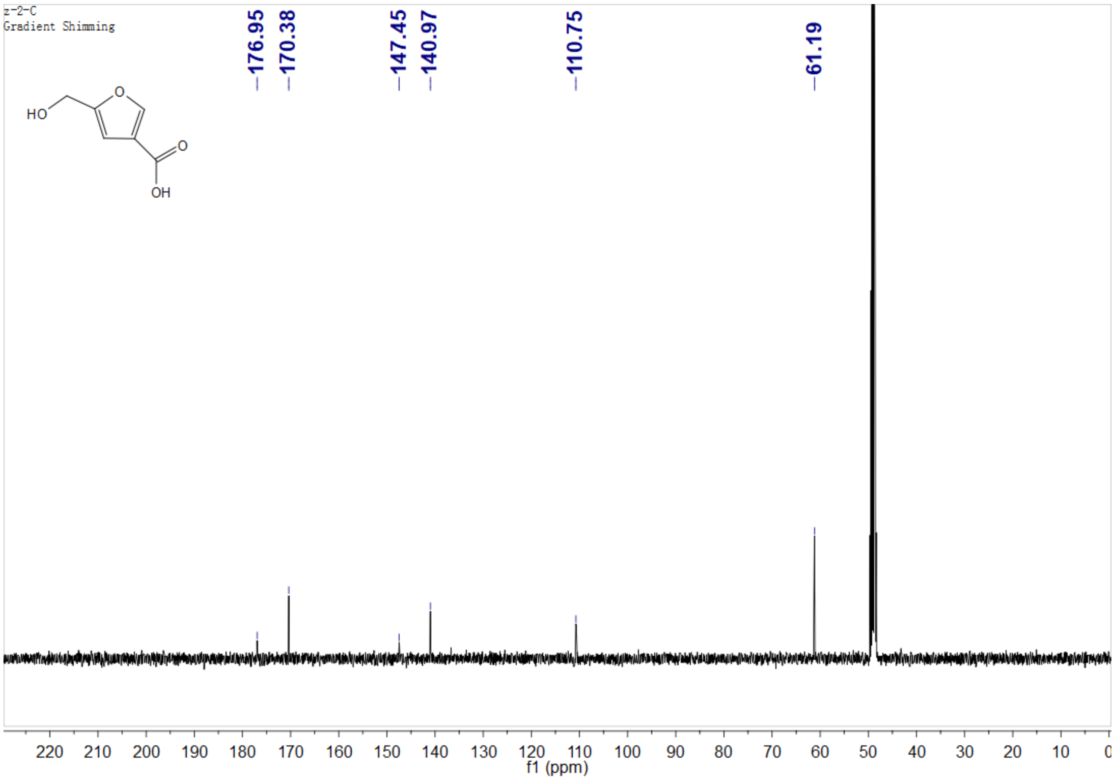

Supplementary Figure 56. ^{13}C NMR data for compound **18** (150 MHz, CD_3OD)

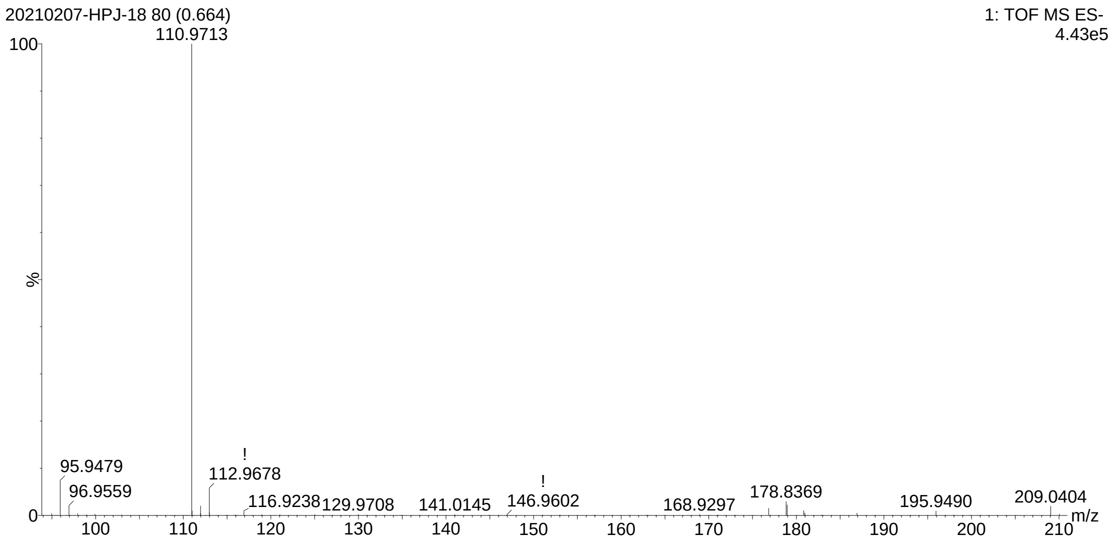

Supplementary Figure 57. HRMS data for compound **18**

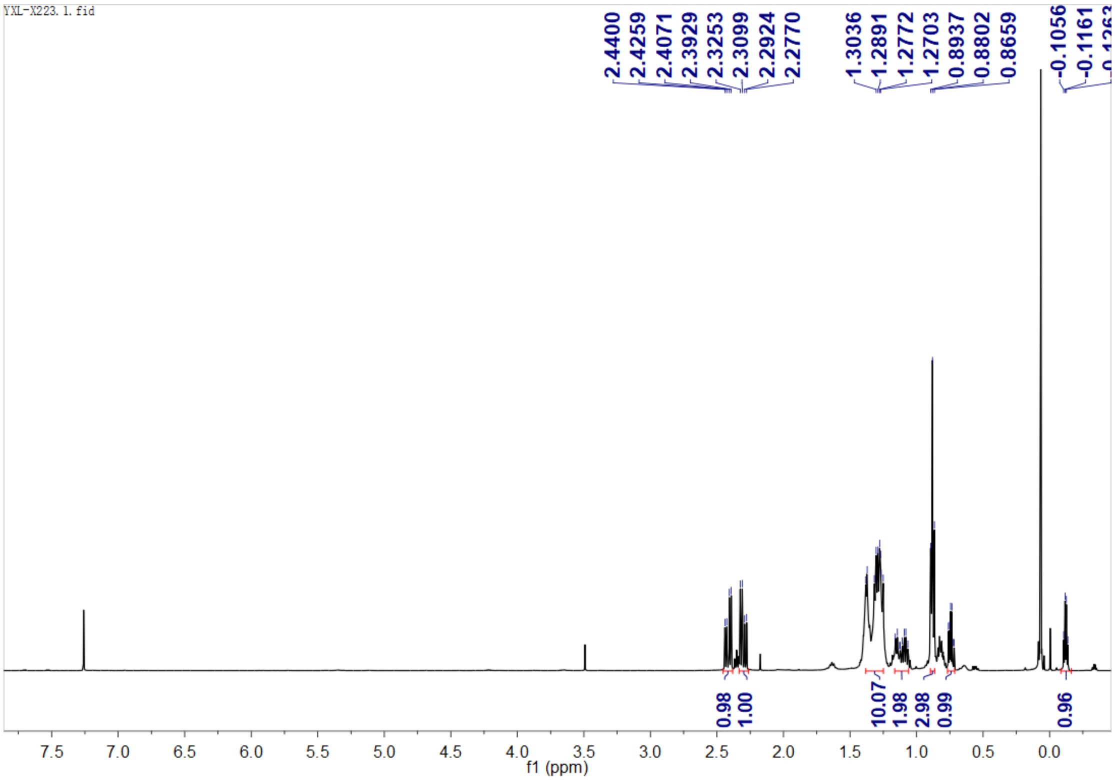

Supplementary Figure 58. ^{1}H NMR data for compound **19** (600 MHz, $CDCl_3$)

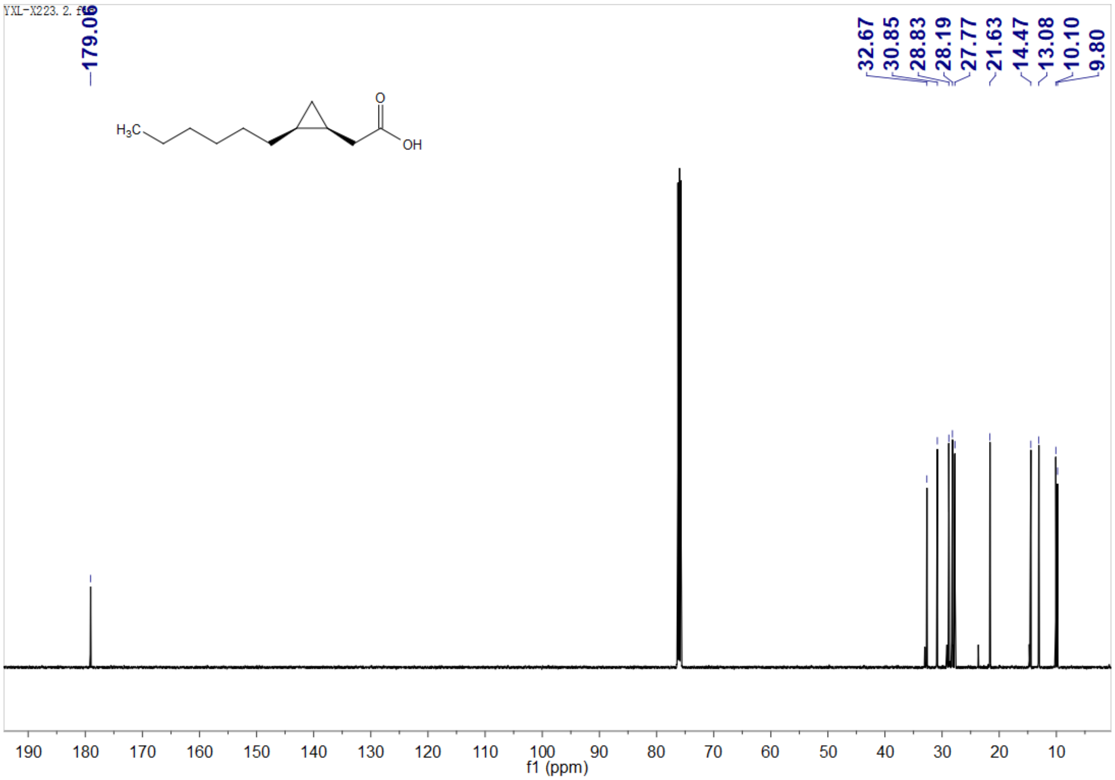

Supplementary Figure 59. ^{13}C NMR data for compound **19** (150 MHz, $CDCl_3$)

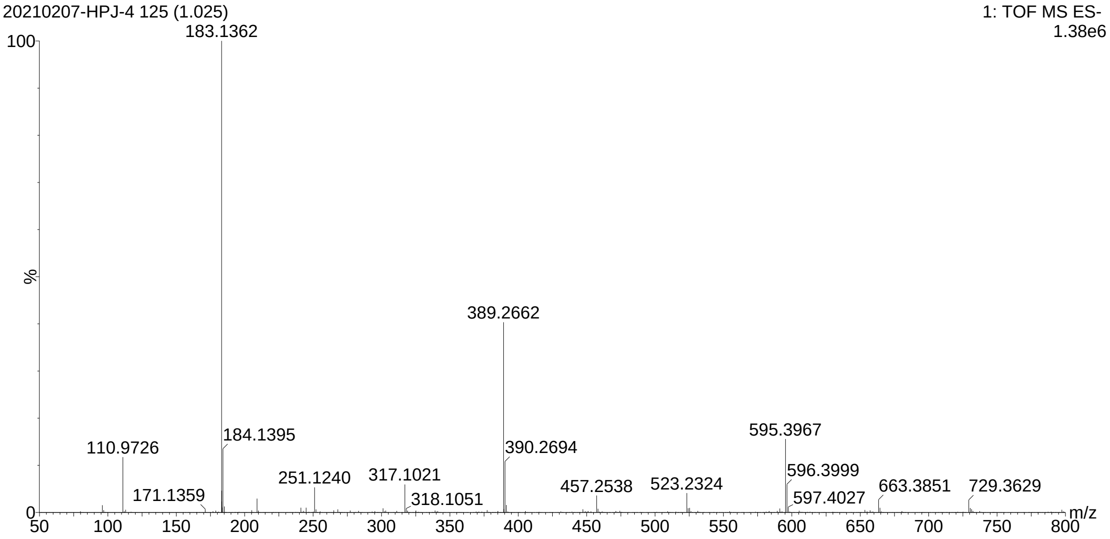

Supplementary Figure 60. HRMS data for compound **19**
